# Population-wide sampling of retrotransposon insertion polymorphisms using deep sequencing and efficient detection

**DOI:** 10.1093/gigascience/gix066

**Published:** 2017-07-31

**Authors:** Qichao Yu, Wei Zhang, Xiaolong Zhang, Yongli Zeng, Yeming Wang, Yanhui Wang, Liqin Xu, Xiaoyun Huang, Nannan Li, Xinlan Zhou, Jie Lu, Xiaosen Guo, Guibo Li, Yong Hou, Shiping Liu, Bo Li

**Affiliations:** 1 BGI Education Center, UCAS: Building 11, Beishan Industrial Zone, Yantian District, Shenzhen, 518083, China; 2 BGI-Shenzhen: Building 11, Beishan Industrial Zone, Yantian District, Shenzhen, 518083, China; 3 BGI College: Building 11, Beishan Industrial Zone, Yantian District, Shenzhen, 518083, China; 4 Department of Biology, University of Copenhagen: Nørregade 10, Copenhagen 1165, Denmark; 5 School of Biology and Biological Engineering, SCUT: Postdoctoral Apartment Building, South China University of Technology, Wushan RD., TianHe District, Guangzhou, 510640, China; 6 BGI-Forensics: Building 11, Beishan Industrial Zone, Yantian District, Shenzhen, 518083, China

**Keywords:** transposable element, retrotransposon insertion polymorphism, next-generation sequencing, whole-genome sequencing

## Abstract

Active retrotransposons play important roles during evolution and continue to shape our genomes today, especially in genetic polymorphisms underlying a diverse set of diseases. However, studies of human retrotransposon insertion polymorphisms (RIPs) based on whole-genome deep sequencing at the population level have not been sufficiently undertaken, despite the obvious need for a thorough characterization of RIPs in the general population. Herein, we present a novel and efficient computational tool called Specific Insertions Detector (SID) for the detection of non-reference RIPs. We demonstrate that SID is suitable for high-depth whole-genome sequencing data using paired-end reads obtained from simulated and real datasets. We construct a comprehensive RIP database using a large population of 90 Han Chinese individuals with a mean ×68 depth per individual. In total, we identify 9342 recent RIPs, and 8433 of these RIPs are novel compared with dbRIP, including 5826 Alu, 2169 long interspersed nuclear element 1 (L1), 383 SVA, and 55 long terminal repeats. Among the 9342 RIPs, 4828 were located in gene regions and 5 were located in protein-coding regions. We demonstrate that RIPs can, in principle, be an informative resource to perform population evolution and phylogenetic analyses. Taking the demographic effects into account, we identify a weak negative selection on SVA and L1 but an approximately neutral selection for Alu elements based on the frequency spectrum of RIPs. SID is a powerful open-source program for the detection of non-reference RIPs. We built a non-reference RIP dataset that greatly enhanced the diversity of RIPs detected in the general population, and it should be invaluable to researchers interested in many aspects of human evolution, genetics, and disease. As a proof of concept, we demonstrate that the RIPs can be used as biomarkers in a similar way as single nucleotide polymorphisms.

## Findings

### Introduction

Transposable elements (TEs) are genomic sequences that can replicate within the genome either autonomously or in conjunction with other TEs, resulting in insertion polymorphisms. Over the evolutionary timescale, this process leads to drastic changes in genomic structure. Current estimates suggest that approximately half of the human genome is derived from TEs [1]. Retrotransposons, which constitute ∼93% of TEs [2], can be subdivided into those sequences that contain long terminal repeats (LTRs) and those that do not (non-LTR). The majority of human TEs result from the activity of non-LTR retrotransposons, including long interspersed nuclear element 1 (L1), Alu, and SVA elements, which collectively account for approximately one-third of the human genome [1]. Although most retrotransposons are inactive remnants prevalent among the human population, younger retrotransposons account for much of the structural variation among individual genomes [3]. Only a small proportion of total L1s are highly active [4]. The current rate of retrotransposition in humans has been approximately estimated as 1 for every 20 births for Alu, 1 for every 200 births for L1, and 1 for every 900 births for SVA [5, 6].

Retrotransposon insertion is a disease-causing mechanism [7], and next-generation sequencing (NGS) technology has been widely used to explore the association between retrotransposon insertions and disease, such as cancer [8–10]. In this respect, a comprehensive retrotransposon insertion polymorphism (RIP) dataset of a healthy population is necessary to serve as a reference for the identification of disease-related RIPs. Using the database of the 1000 Genomes Project (1000GP), researchers performed RIP detection on an unprecedented scale and detected thousands of novel RIPs [11–14]. This finding implies that an insertion allele present in multiple individuals would effectively receive high coverage across the pooled dataset, leading to a detection bias toward common insertions. It was previously estimated that at least ×30 coverage of sequencing is needed to detect heterozygous RIPs with high sensitivity using whole-genome sequencing (WGS) [15].

Here, we developed the software Specific Insertions Detector (SID) to detect RIPs, which fulfilled our needs regarding detection efficiency, accuracy, and sensitivity. We also generated a non-reference TE insertion polymorphism database by employing SID to analyze the whole-genome sequences of 90 Han Chinese individuals (YH90) acquired at a mean depth of ×68.

## Materials and Methods

### Samples and whole-genome sequencing

We obtained B-lymphocyte cell lines from 90 Han Chinese individuals at the Coriell Institute (Camden, NJ, USA). These individuals were selected from Beijing, Hunan province and Fujian province, respectively. We broadly separated the samples into a “Northern group” (45 samples) and a “Southern group” (45 samples). DNA was extracted from the B-lymphocyte cells of each individual, and libraries were then constructed following the manufacturer's instructions. High-coverage paired-end 100 bp WGS libraries were sequenced on the Illumina HiSeq 2000 Platform. For more on this dataset, see the Data Note describing its production published alongside this paper [16]. In addition, we also used a Chinese sample [17] for which the data were previously released in the European Nucleotide Archive (ENA) repository (Additional file 1: Table S1). The Institutional Review Board on Bioethics and Biosafety at BGI (BGI-IRB) approved the study.

### Processing of the WGS data

Reads were aligned to the human genome reference (HG19, Build37) using *BWA* (BWA, RRID:SCR_010910) [18]. Duplications were removed using Picard tools, and the quality values of each read were recalibrated using the Genome Analysis Toolkit (GATK, RRID:SCR_001876) [19]. The resulting Binary Alignment/Map (BAM) files were used as input for SID (Additional file 2: Text S1).

### The specific insertion detector pipeline

SID is compiled in Perl and includes the following 2 steps: discordant reads detection and reads clustering. Generally, the first step collects informative reads and generates other necessary files, whereas the second step discovers the specific insertion sites and exports the final results into plain text.

#### Detection of discordant reads

The “discordant reads” were extracted for the subsequent clustering step. Paired-end reads were determined as “discordant reads” if they met 1 of the following criteria: (i) 1 read mapped to HG19 uniquely and the other read mapped to the retrotransposon library (multi-mapped or unmapped to HG19); (ii) 1 read mapped to HG19 uniquely and the other soft-clipped read mapped to HG19, and the clipped sequence could be mapped to the retrotransposon library; (iii) 1 soft-clipped read mapped to HG19, and the clipped sequence could be mapped to the retrotransposon library. The other read mapped to the retrotransposon library (multi-mapped or unmapped to HG19). The retrotransposon library includes objective TE classes, such as L1, Alu, and SVA. In this study, the TE reference database contains known TE sequences collected from RepBase v. 17.07 [20], dbRIP [21], and Hot L1s [4]. To reduce the long processing time due to large volumes of WGS data, we implemented a parallel approach to process all BAM files of samples simultaneously in the discordant reads detection step.

#### Reads clustering and detection of breakpoints

First, the “discordant reads” were scanned and clustered into blocks that supported potential RIPs based on the Maximal Valid Clusters algorithm [22]. Second, we extracted all reads located within the cluster regions and determined the breakpoints. Although high-depth, data-enabled RIP detection with high sensitivity was possible given that more soft-clipped reads neighboring target site duplication (TSD) could be detected, alignments neighboring the TSDs apparently had lower depth compared with the mean sequencing depth of the whole genome due to occasional sequencing and system errors. This feature made breakpoint detection difficult and increased the false discovery rate (FDR). Thus, we added the recalibration process of clipped points to determine breakpoints. Each read located within the cluster regions flanking potential breakpoints was used to confirm the precise location of the breakpoints. Small deletions were extracted to perform breakpoint recalibration, and the mismatched bases were removed from the deletion sequences.

The clipped sequences were realigned to local regions on HG19 to determine the actual breakpoints. Breakpoints were assigned as “clips” if greater than half of the new clipped sequences were discordant with the reference sequence and the length of the gap within the new clipped sequence was less than 30%. The point would not be a candidate unless it was a “clip” and the mismatch was less than 5 bp or contained poly-A/T.

Some terminals of reads containing mismatched bases may be the clipped parts because these bases were treated as mismatches rather than clips. The breakpoint candidates were re-estimated by SID if mismatches accounted for more than half of the read terminals.

Notably, we implemented the Asynchronous Scanning algorithm (Additional file 2: Text S2). Using this algorithm, once the program clustered 1 possible insertion region by scanning unique reads, the process of breakpoint detection in this region was immediately performed, rendering it possible to detect TE insertions in 1 chromosome in only a few minutes. The detailed algorithm for RIP candidate determination is provided in Additional file 2: Text S2.

### Annotation of TE insertions

#### Orientation annotation for the TE insertions

We annotated the orientation of TE insertions based on the BLAST results [23]. First, we extracted the discordant repeat anchored mate (RAM) reads and clipped reads that supported the TE insertion and made the reads’ orientations the same as HG19. Then, we realigned the supporting reads against the consensus sequences of known active retrotransposons to identify the mapped orientation in known active retrotransposons. The orientations of TE insertions were judged by the reads’ orientation (for details, see Additional file 2: Text S3). The accuracy of orientation annotation was assessed by comparing 396 matched insertions from dbRIP and 21 fully sequenced insertions from polymerase chain reaction (PCR) validation experiments (Additional file 1: Table S2). In total, 326 insertions were verified, and the FDR of orientation annotation was 21.82%.

#### Subfamily annotation for RIPs

The subfamily annotation of RIPs was performed according to known active retrotransposons. We first constructed a comprehensive retrotransposons sequence library. Alu subfamily consensus sequences were acquired from RepBase 17.07 [20]. L1 subfamily consensus sequences were acquired from Eunjung Lee [10]. SVA and LTR consensus sequences were acquired from Baillie [24]. Next, we performed multiple subfamily sequence alignment for each type of retrotransposon and discovered the diagnostic nucleotide for each subfamily (for details, see Additional file 1: Tables S3–S5). Specifically, we discovered the diagnostic nucleotide of L1 from previous studies [25–28]. We then assembled the “discordant reads” of each RIP into contigs using CAP3 [29] and realigned them against all of the subfamily sequences using BLAST (NCBI BLAST, RRID:SCR_004870) (Additional file 2: Text S3–S4) [30].

#### Length annotation for RIPs

While mapping the contigs to subfamily sequences, we identified the first mapped site of the 5’ and 3’ ends of the subfamily sequence and accordingly counted the lengths from the initial site (*L*_min_ and *L*_max_). The length of the inserted retrotransposon (*L*_retro_) was calculated as the difference between the maximum and minimum length of the aligned sequence, as follows:

\[
{L_{retro}} = {L_{\max}}\hbox{--}{L_{\min}} + 1.
\]

### Simulation of RIP data

In total, 761 TEs were randomly selected from our reference TE database (see the “Annotation of TE insertions” section) and inserted into HG19 autosomes randomly to generate a new human genome (for details, see Additional file 1: Table S6). The pIRS [31] software was used to generate approximately ×60 paired-end 100-bp reads; then, we mapped these reads to the HG19 genome using BWA. Then, we used SID to detect these RIPs in the simulated genome. By repeating this process, we obtained results from simulated data with different depths to assess the sensitivity and specificity of RIP detection in the sequence data with distinct depth using SID.

### Reference RIP detection

The reference RIPs were detected as a subset of deletions of the samples relative to the HG19 reference (Additional file 2: Fig. S1). These deletions were selected from the results of structural variation (SV) detection of YH90 samples, and the RIPs were annotated based on matched deletion coordinates to HG19 annotation of RepeatMasker (more than 90% of them overlap with each other) [32].

The reference RIPs should be absent in the chimpanzee genome. The alignments of chimpanzee mapped to the human genome were downloaded from UCSC [33]. One reference RIP candidate should correspond to a gap with an overlap of greater than 90% to each other, and no gaps were present in the chimpanzee genome at this locus. The RIP candidates were filtered if no polymorphisms were present in the YH90 samples (i.e., the allele frequency was equal to 180).

## Results

### Establishment of SID

**Figure 1:**
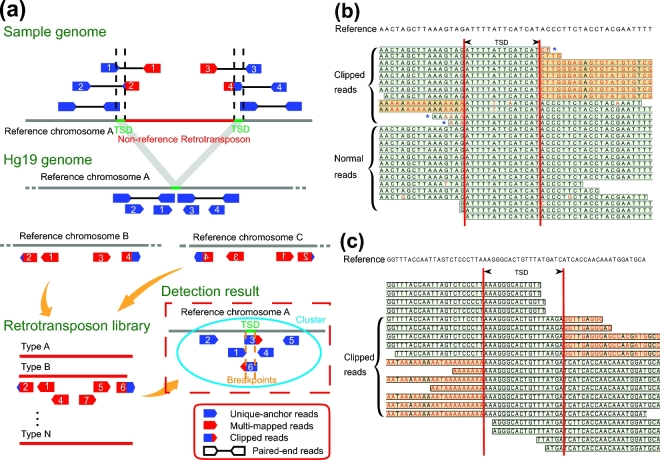
The principle of retrotransposon insertion detection. (**a**) Schematic diagram of using SID for RIP detection in the genome. SID: Specific Insertions Detector; TSD: target site duplication. (**b**) An example of reads mapping for predicted homozygous insertions. (**c**) An example of reads mapping for predicted heterozygous insertions. In (**b**) and (**c**), the red bases indicate the mismatches, and the sequences with an orange background represent the clipped part of the reads. The clipped reads are derived from 1 allele with inserted retrotransposons, and the normal reads are derived from the other allele with the same reference. The 3 reads with asterisks indicate no clipped part but the presence of terminal mismatches, which can also support the breakpoint and exhibit consistency with the clipped reads.

To detect non-reference RIPs from WGS data accurately and in a time-efficient manner, we developed SID, which can detect non-reference RIPs easily and quickly through discordant reads detection and reads clustering. In the first step, 3 types of informative discordant reads were selected for further analysis (Fig. 1a). Then, the reads that had mismatched bases at the terminals (Fig. 1b and c) were used for judging heterozygosity. The clipped reads were used to confirm the sequence of TSD and the precise insertion site of certain TEs.

### Non-reference retrotransposon insertion calling

**Figure 2:**
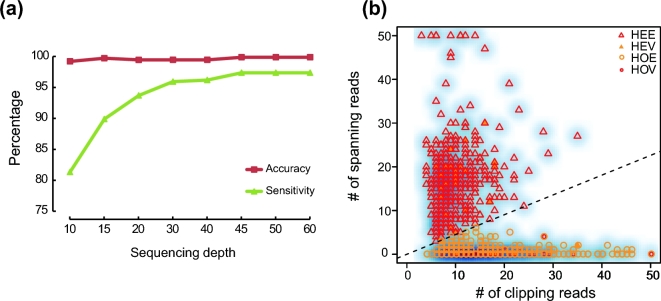
Assessing the SID results. (**a**) Detecting accuracy and sensitivity estimation along cumulating sequencing depth of simulated data. (**b**) RIP genotyping of YH_CL. PCR validation results are marked. HEE: estimated heterozygous site; HEV: validated heterozygous site; HOE: estimated homozygous site; HOV: validated homozygous site. The dashed line indicates the estimated boundary between heterozygous and heterozygous sites. Note that some of the validated RIPs are present in the same locus in the plot figure.

To investigate the influence of sequencing depth on RIP detection sensitivity and accuracy, we simulated sequence data at different depths. Detection sensitivity dramatically increased with increasing sequencing depth and achieved 95% (730/761) when the sequencing depth was greater than ×30. By contrast, detection accuracy slightly changed with increasing sequencing depth (Fig. 2a).

We next estimated the RIP detection sensitivity using 2 real sequencing datasets. One dataset was the CEU trio data, which were deep-sequenced (>×75) Illumina HiSeq data generated by the Broad Institute (father NA12891, mother NA12892, and the female offspring NA12878) from the 1000GP. We first used SID to detect the RIPs of each individual in the CEU dataset and evaluated the sensitivity by comparing the detection results with the PCR-validated datasets from Stewart et al. [12]. For Alu, the mean sensitivity reached 96.3% among individuals. We also obtained a mean sensitivity of 80.3% and 83.3% for L1 and SVA, respectively (Additional file 1: Table S7).

The other dataset, including NA18571, NA18572, and NA18537, was also recruited in 1000GP. The RIP datasets of these 3 individuals detected by SID were larger and covered 70.08% of the same sample's results in 1000GP on average (Additional file 2: Fig. S2). We estimated RIP detection accuracy using the sequencing data from a lymphocytic cell line (YH_CL, ∼×52) obtained from an Asian individual. These data represent the first Asian diploid genome dataset, and we performed PCR validation. We randomly selected 103 detected RIPs, and 93/96 (7 loci were removed because of the poor primer specificity) loci were successfully validated, indicating that SID had an accuracy of 90.29–96.88% (Additional file 1: Table S8 and Additional file 2: Fig. S3 and Text S5). We also used the PCR validation result to access the accuracy of genotyping, which was approximately 93.55% (87/93) (Fig. 2b; Additional file 2: Text S6).

We next compared the RIP detection efficiency of different methods (SID, RetroSeq [11], and TEA [10]) using YH_CL and 3 samples (NA18571, NA18572, and NA18537) from YH90 (Additional file 2: Text S7). The run time of SID was approximately 3-fold reduced compared with the other 2 methods, suggesting that SID was the most time-saving method among the 3 methods (Additional file 2: Table S9). SID and TEA had comparable sensitivities that were increased compared with RetroSeq (Additional file 2: Fig. S4). We also validated the uniquely detected RIPs by PCR (Additional file 1: Table S10) with an accuracy of 75.86% (22/29) and 77.78% (7/9) for Alu and L1, respectively, revealing a higher RIP detection accuracy (Alu: 42.10% (8/19) and 82.61% (19/23) and L1: 66.67% (2/3) and 66.67% (2/3) for RetroSeq and TEA, respectively).

### A comprehensive RIP landscape of the Han Chinese population

We then performed RIP detection on a much larger scale. We sequenced 90 Han Chinese individuals and generated Illumina paired-end sequence data at an average depth of ×68 for each sample (Additional file 1: Table S1). Using SID, the high depth of the dataset (much more than ×30) allowed us to build a comprehensive non-reference RIP landscape with high confidence [16].

**Figure 3:**
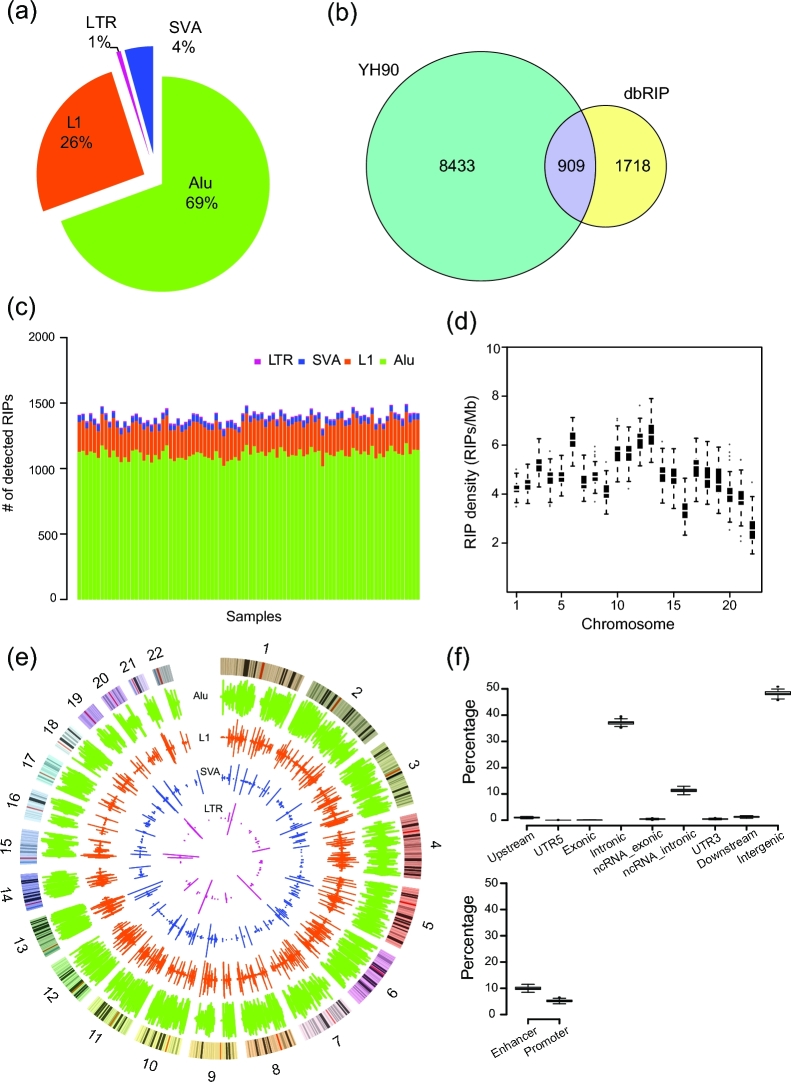
Comprehensive landscape of non-reference RIPs of YH90. (**a**) Proportions of novel insertions identified for each type of retrotransposon. (**b**) Comparison of YH90 non-reference RIP results with dbRIP. Adjacent 100-bp regions of RIPs were taken into consideration. (**c**) TE distribution of each YH90 sample. (**d**) Box plots of non-reference RIP distribution among autosomes. (**e**) TE frequency distribution among YH90 samples. Rings from outer to inner indicate Alu insertion frequency, L1 insertion frequency, SVA insertion frequency, LTR insertion frequency, and cytoband structure. The inside frequency of the rings indicates the insertion frequency for the Northern Chinese group, and the outside frequency represents that of the Southern Chinese group. (**f**) RIP distribution in different functional regions of the genome.

In total, we identified 9342 non-reference RIPs in autosome regions, including 6483 Alu elements, 2398 L1s, 61 LTRs, and 400 SVAs (Fig. 3a; for details, see Additional file 1: Table S11 and Additional file 2: Text S8). Of this dataset, 8433 RIPs, including 5826 Alu elements, 2169 L1s, 383 SVAs, and 55 LTRs, were novel compared with dbRIP (Fig. 3b). The average number of non-reference RIPs per individual was 1394 (ranging from 1304 to 1493) (Fig. 3c), including 1110.80 Alu elements, 231.34 L1s, 43.14 SVAs, and 9.01 LTRs, and each type of RIP had a similar proportion (*P* = 0.6364, *P* = 0.2711, *P* = 0.2128, *P* = 0.5582, respectively, Wilcoxon signed-rank test). We compared pair-wise individuals of all 90 samples, and the average specific loci number was 672.79, which is approximately half (48.25%) the non-reference RIPs of 1 individual.

We next compared our results with the 1000GP SV dataset. In total, 34.94% (3264/9342) of the RIPs in YH90 were also found in the 1000GP dataset. The Pearson correlation coefficient was 0.7998 (*P* < 2.2 × 10^−16^) between YH90 and all the 26 populations in the 1000GP SV dataset. The Pearson correlation coefficient was 0.8856 between YH90 and the East Asian (EAS) population in 1000GP, which was higher than other populations (*r* = 0.7662, *r* = 0.5741, *r* = 0.7025, and *r* = 0.7627 for American [AMR], African [AFR], European [EUR], and South Asian [SAS] populations, respectively) (Additional file 2: Text S9) [14].

Specific insert location information enabled us to investigate genome-wide sequence patterns of these non-reference RIPs. We observed that the non-reference RIPs varied among chromosomes (Fig. 3d and e). Notably, we found that the 2 different subpopulations (from southern and northern China) had similar patterns of RIP distribution (*r* = 0.782) (Fig. 3e; for details, see Additional file 2: Fig. S5). However, the distribution of non-reference RIPs was not obviously correlated with GC content, fixed RIPs, or single nucleotide polymorphisms (SNPs) of the same sample within 10M non-N bins (Additional file 2: Fig. S6).

To further investigate the distribution of non-reference RIPs in the functional region, we annotated all the inserted loci (Fig. 3f). More than half of the RIPs (4828/9342) were located in gene regions, and the majority of these were located in introns. Only 5/9342 RIPs were located in protein-coding regions, including 3 genes, C1orf66 (Alu-inserted), SNX31 (Alu-inserted), and APH1B (SVA-inserted), with low frequency (1/90) and 2 genes, ADORA3 (Alu-inserted) and Slco1b3 (L1-inserted), with higher frequency (44/90 and 12/90, respectively). In addition to gene regions, we also found that on average 9.78% and 4.93% of RIPs were located in enhancer regions and promoter regions per sample, respectively (Fig. 3f).

Furthermore, we annotated the subfamily, orientation, and sequence length of all detected inserted retrotransposons based on regional sequence assembly and remapping to the retrotransposon library. The AluY sub-family constituted essentially all non-reference Alu insertions, in which AluYa5 and AluYb8 were mostly active (Additional file 1: Table S11), supporting conclusions from previous studies [26, 34, 35].

The orientation of 1 RIP is determined from the mapping orientation of contigs to a retrotransposon reference and the existence of poly-A or poly-T tails of the inserted sequence (Additional file 1: Table S11). Previous studies have reported that the gene-inserted RIP had a greater influence on gene expression if it was inserted on the same orientation as the target gene [2, 36]. However, we detected a comparable number of direct and reverse events (0.475 and 0.525, respectively), arguing against an obvious natural selection on the RIPs with consistent orientation with the inserted gene.

Along with subfamily and orientation annotation, we also calculated the length of each insertion sequence. We found that different types of TE insertions had different length distributions (Additional file 2: Fig. S7). More than half of Alu elements (∼70%) were full length, whereas the length of the L1 was distributed more discretely. Most L1s (>80%) were fractured during the process of retrotransposon, which is consistent with a previous study [13].

### RIPs of a healthy population

The pure and comprehensive RIP dataset can be used as a baseline of healthy people for other disease-related research, especially single-gene diseases. The candidate disease-related retrotransposon insertions found in this dataset were filtered. We explicitly measured the overlap between our dataset and the disease-related retrotransposon insertion data in dbRIP [37, 38]. None of the insertion sites existed in our dataset, indicating the accuracy of the database. We also tested some cancer research data. We tested the dataset of candidate cancer-related somatic retrotransposon insertions that were strictly generated from data of The Cancer Genome Atlas (TCGA) Pan-Cancer Project for 11 tumor types. No overlapping RIPs were detected, whereas 43.36% germline retrotransposons were detected. According to the comparison of colon cancer–specific data [9], we identified 2 L1 insertions consistent with our dataset with frequency of 51/90 and 50/90. These 2 L1 insertions were germline retrotransposon insertions that were further validated by PCR validation in Solyom's research. We also tested the candidate hepatocellular carcinoma-specific insertions [8] and identified 1 L1 insertion that was also present in our dataset with a frequency of 9/90. This site was finally validated as a germline insertion by PCR in that research. In conclusion, our data provide a reference panel to exclude false positive insertions related to cancer.

### Population evolution analysis

To perform the population evolution analysis of RIPs, we first merged the non-reference RIP dataset with the “reference” retrotransposon insertions that were polymorphic in YH90 samples (Additional file 2: Fig. S1) to obtain all RIPs from our samples. The retrotransposon insertions with a frequency equal to 1 were removed from our non-reference RIPs. The “reference” RIPs were defined as the reference genome-specific retrotransposon insertions compared with each individual of the YH90 group. These reference RIPs were selected from the dataset of YH90 deletions, and only the RIPs absent in chimpanzees were retained.

Allele frequency spectrum (AFS) was not only influenced by natural selection but also by demographic history. For example, a low-frequency bias for the majority of mutations can also be obtained if the population recently experienced a bottleneck [39].

**Figure 4:**
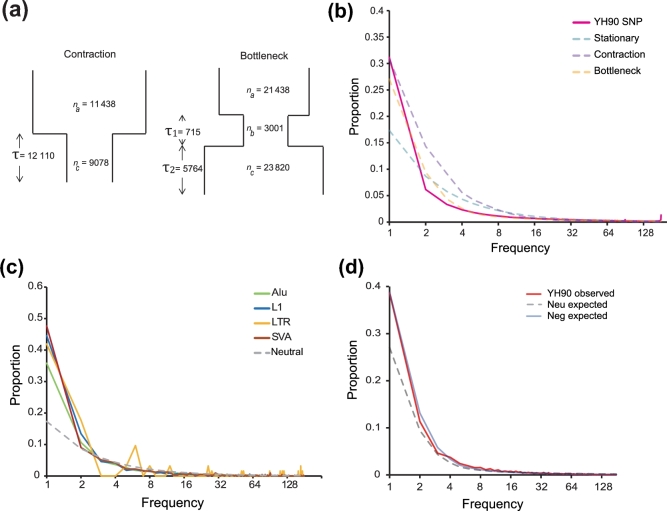
Population genetics analysis based on YH90. (**a**) A 2-epoch population with a recent contraction; a 3-epoch bottleneck-shaped history, which contained a reduction of the effective population size in the past followed by a recent phase of size recovery. Details of the parameters for all models are provided in Additional file 2: Table S12. (**b**) The observed SNP frequency spectra and expected neutral SNP frequency spectra under different demographic models. (**c**) Observed and expected RIP site frequency spectra before demographic correction of each subfamily. (**d**) Assessing the evolutionary impact of RIPs in the human genome. The allele frequency distribution of RIPs was compared among observed neutral models and negative models after demographic correction.

To perform the neutral test more accurately, we took demographic history into consideration (Additional file 2: Text S10). We simulated the following 2 different demographic scenarios: a 2-epoch population with a recent contraction and a 3-epoch bottleneck-shaped history containing a reduction of effective population size in the past followed by a recent phase of size recovery (Fig. 4a). We tested the different assumptions with the SNP dataset (Fig. 4b; Additional file 2: Table S12), which supported that the 3-epoch model was the best model.

Next, we explored the possibility of using RIP information to perform population evolution analysis. Based on the genotyping result of the merged RIP dataset, we described the RIP AFS (Fig. 4c; Additional file 2: Text S11). The neutral model expectation can be calculated using the formula θ/*i*, where θ is the insertion diversity parameter and *i* (180) is the allele count in a fixed number of samples *n* (90) [39]. The spectrum was skewed toward low-allele frequency compared with the distribution of the expected neutral model, indicating possible negative selection pressure on retrotransposon insertions.

To investigate the influence of the demographic history on RIP AFS, we performed demographic correction and re-analyzed the RIP AFS under different selection models (Fig. 4d; Additional file 2: Figs S8–S9). The classification of neutral with negative and positive selection indicates that a proportion of RIPs were neutral, and a proportion of RIPs were under negative selection. In addition, other RIPs were under positive selection (m1), neutral with negative selection (m2), neutral with positive selection (m3), negative selection (m4), positive selection (m5), and neutral selection (m6). We further calculated the selection coefficient (*S*') under each best-fit model with the determination of an approximately neutral selection effect threshold (*S*' < 0.01%) [40]. Models m1 and m2 were the best-fitted models with the observed RIP AFS (Additional file 2: Table S13). The best-fit result of model m1 demonstrated that approximately 75% RIPs were under negative selection, with s = 0.0290%, which indicates that these RIPs are weakly deleterious. In addition, 10% were under positive selection, whereas 15% were neutral. Under model m2, the best-fit result demonstrated that 70% of RIPs were under negative selection, with s = 0.0396%. In addition, 30% of RIPs were neutral. The selection coefficient was 0.0079% under the all negative selection models, indicating an approximately neutral selection effect.

The distribution of fitness effects of retrotransposon subfamilies (L1, SVA, and Alu) was also estimated under the same demographic model. Assuming that all RIPs of different subfamilies were under negative selection (model m1), the selection coefficient models were various among 3 subfamilies of RIPs (*S^΄^* = –0.0143%, *S^΄^* = –0.0172%, *S^΄^* = –0.0068% for L1, SVA, and Alu, respectively), suggesting that there is more natural selection pressure on L1 and SVA (weakly negative selection) compared with Alu (nearly neutral selection).

### Phylogenetic analysis

**Figure 5:**
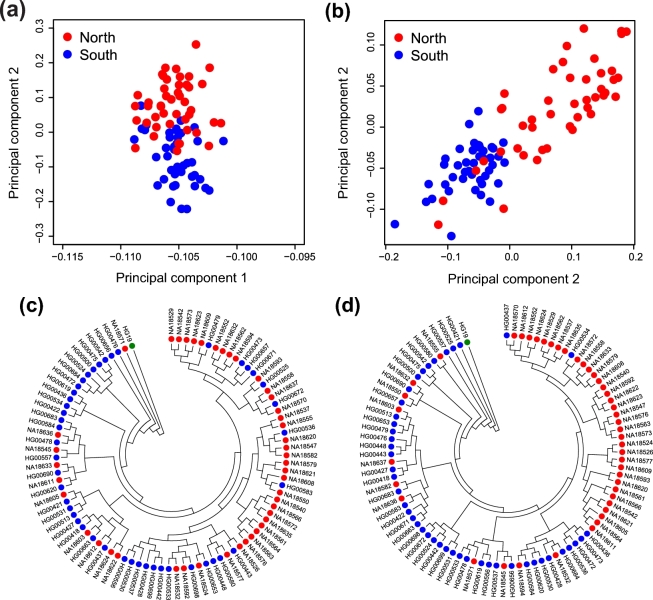
Phylogenetic analysis using RIPs and SNPs. (**a**) The detected RIPs were used for PCA. Each dot represents a sample from YH90 and is plotted in a scatterplot using PC1 and PC2. Red indicates samples from individuals from northern China, and blue indicates individuals from southern China. (**b**) The detected SNPs were used for PCA. The plot layout and legend are the same as those presented in (**a**). (**c**) Phylogenetic tree constructed using the detected RIPs. HG19 (green) is used as a control. Red indicates samples from individuals from northern China, and blue indicates samples from individuals from southern China. (**d**) Phylogenetic tree constructed using the detected SNPs. HG19 (green) is used as a control. Plot layout and legend are the same as that presented in (**c**).

To investigate whether RIP information can be used to separate the Northern and Southern Chinese groups, we performed principal component analysis (PCA) using the RIPs detected from the YH90 dataset, which provided well-resolved Northern and Southern Chinese groups (Fig. 5a; Additional file 2: Text S12). Compared with the PCA result derived from the SNPs detected from the same dataset (Fig. 5b), there seemed to be more overlapping observations, indicating that SNPs might be more informative in resolving the 2 distinctive populations. Next, we determined whether it is possible to perform phylogenetic analysis using RIP information detected from the YH90 dataset. Two phylogenetic trees were constructed using RIPs and SNPs separately (Fig. 5c and d; for details, see Additional file 2: Text S13). Similar to the PCA result, increased mixing between Northern and Southern Chinese individuals was observed for the phylogenetic tree derived from the RIP information. Interestingly, HG00534, an isolated Southern Chinese individual located in a northern cluster in the phylogenetic tree established using the SNP information, clustered largely with Southern Chinese individuals in the phylogenetic tree derived from the RIP information. Future studies are warranted to explore whether combining SNPs with RIP results in the construction of a more accurate phylogenetic tree.

## Conclusions

In this paper, we developed the computer program SID to detect the non-reference RIPs of 90 healthy Han Chinese individuals using high-depth WGS. We described the landscape of RIP distribution on population genomes and annotated the subfamily, orientation, and length of RIPs. We demonstrated that the RIPs could be used as a normal baseline for retrotransposon-related disease research.

To our knowledge, this is the largest Han Chinese genomics dataset to date. Compared with 1000GP results from the same samples, approximately half (mean = 48.05%) (Additional file 2: Fig. S2) of the RIPs in our dataset were previously observed, suggesting that our deep-sequenced data exhibited increased detection sensitivity compared with low-coverage data. For example, serum ACE levels were determined by the Alu insertion/deletion (I/D) polymorphism in the following order: DD > ID > II [41]. The D allele of the ACE gene was associated with essential hypertension in different populations [42–45]. We found that the ACE gene harbored an Alu insertion in the 15th intron, with a frequency of 81/90 in our 90 Chinese genomes, compared with a considerably reduced frequency (7/63) in CEPH individuals [12], which was supported by a previous study [46]. To our surprise, no RIP ACEs were present in Han Chinese samples from the 1000GP dataset, which is a high-frequency inserted gene in our RIP data. ACE-specific PCR validation (Additional file 2: Fig. S10) and a previous ACE study [47] indicated that our results were consistent with the real values. This finding suggests that adequate sequencing depth is important in investigating RIP frequency and that our data present a result that is consistent with the actual situation. The highly sensitive and accurate RIP dataset provided a perfect opportunity to perform RIP fitness analysis. This study evaluates the natural selection effect on retrotransposon insertions at the population level. As a type of long fragment insertion, RIPs are under approximately neutral selection. This finding is consistent with our result that retrotransposon insertions are mostly relatively inconsequential because the harbored genes are always relatively unimportant. Regarding different types of RIPs in addition to Alu, the longer insertion elements L1 and SVA exhibit weakly positive selection pressure.

This dataset can be compared with others to provide guidance in research of the disease-causing mechanisms in certain populations and to successfully determine the insertion time of a specific locus. This dataset can also be used as a standard for other RIP research and can serve as a baseline to filter irrelevant RIPs in disease-causing retrotransposon research. Genome-wide association studies (GWAS) have proven their utility in identifying genomic variants associated with the risk for numerous diseases. Unlike SNPs and copy number variations (CNVs) that are widely used in GWAS, RIPs have generally been overlooked as a major contributor to human variation. Significantly, this dataset provides a valuable resource to perform GWAS and identify more markers related to complex diseases.

The high cost of WGS at high depth is still a major limitation, preventing it from being widely used in TE research. Furthermore, the large amount of data yielded by high-depth WGS makes it difficult to undertake bioinformatic analysis. With the development of biotechnology and IT, this situation should improve soon.

The next step is to research RIPs at the transcriptome level. The impact of RIPs on gene expression remains unclear. Combining the genome and transcriptome would provide a comprehensive picture about the regulation of RIPs. Thus, we can further expound the position of the retrotransposon in the course of human evolution.

## Availability and requirements

- Project name: Specific Insertions Detector (SID)
- Project home page: https://github.com/Jonathanyu2014/SID
- Operating system(s): Linux
- Programming language: Perl
- Other requirements: Perl 5.14 or later, BLAST v. 2.2.25 or later, Samtools v. 1.0 or later
- License: Apache License 2.0
- Any restrictions to use by non-academics: none

## Availability of data and materials

The source code of SID is available from the GitHub and Zenodo repositories [48]. The human (*Homo sapiens*) reference genome sequence (HG19) and its annotation files were downloaded from UCSC Genome Bioinformatics (http://genome.ucsc.edu/). The raw sequence data of the CEU trio is available from www.internationalgenome.org/data-portal/sample. [49]. All the YH90 raw sequences have been released to the ENA repository (bioproject number PRJEB11005), and the processed data are also available from the *GigaScience Giga*DB repository [50]. Snapshots of the code, alignments, and results files are also hosted in *Giga*DB [51]. Protocols used for simulating reads for SNP Indel calling and detection of transportable element insertions are also hosted in the protocols.io repository [52, 53].

## Additional files

Additional file 1: Supplementary tables. Data description and the results of RIP calling (XLSX 1992 kb).

Additional file 2: Supplementary texts, figures, and tables (PDF 1120 kb).

## Abbreviations

CNV: copy number variation; ENA: European Nucleotide Archive; GWAS: genome-wide association study; L1: long interspersed nuclear element 1; LTR: long terminal repeat; NGS: next-generation sequencing; PCA: principal component analysis; RIP: retrotransposon insertion polymorphism; SID: Specific Insertions Detector; SNP: single nucleotide polymorphism; TCGA: The Cancer Genome Atlas; TE: transposable element; TSD: target site duplication; WGS: whole-genome sequencing.

## Ethics, consent, and permissions

This study was approved by BGI-IRB (No. 16101).

## Consent to publish

Both BGI-IRB and the involved participants consented to the publication of this research.

## Competing interests

The authors declare that they have no competing interests.

## Author contributions

B.L., S.L., and Y.H. initiated this project and reviewed the manuscript. Q.Y., X.Z., Y.Z., and X.H. drafted the manuscript. X.H. and J.L. edited the manuscript. Q.Y., W.Z., X.Z., and Y.W. performed the data analysis and drew the pictures. Y.Z. and Y.W. designed and developed the SID program. N.L., X.Z., and G.L. conducted the experiment for sequencing. L.X. designed the primers and performed PCR validation. Y.H., B.L., S.L., X.Z., X.G., and X.H. provided fruitful discussions.

## Supplementary Material

GIGA-D-16-00169_Original_Submission.pdf

GIGA-D-16-00169_Revision-1.pdf

GIGA-D-16-00169_Revision-2.pdf

Response-to-Reviewer-Comments_Original-Submission.pdf

Response-to-Reviewer-Comments_Revision-1.pdf

Reviewer-1-Report-(Original-Submission).pdf

Reviewer-1-Report-(Revision-1).pdf

Reviewer-2-Report-(Original-Submission).pdf

Reviewer-2-Report-(Revision-1).pdf

Additional file 1: Supplementary tables. Data description and the results of RIP calling (XLSX 1992 kb).

Additional file 2: Supplementary texts, figures, and tables (PDF 1120 kb).
