# Correction to: Population-wide sampling of retrotransposon insertion polymorphisms using deep sequencing and efficient detection

**DOI:** 10.1093/gigascience/giz008

**Published:** 2018-12-11

**Authors:** Qichao Yu, Wei Zhang, Xiaolong Zhang, Yongli Zeng, Yeming Wang, Yanhui Wang, Liqin Xu, Xiaoyun Huang, Nannan Li, Xinlan Zhou, Jie Lu, Xiaosen Guo, Guibo Li, Yong Hou, Shiping Liu, Bo Li

**Affiliations:** 1 BGI Education Center, UCAS: Building 11, Beishan Industrial Zone, Yantian District, Shenzhen, 518083, China; 2 BGI-Shenzhen: Building 11, Beishan Industrial Zone, Yantian District, Shenzhen, 518083, China; 3 BGI College: Building 11, Beishan Industrial Zone, Yantian District, Shenzhen, 518083, China; 4 Department of Biology, University of Copenhagen: Noregade 10, Copenhagen 1165, Denmark; 5 School of Biology and Biological Engineering, SCUT: Postdoctoral Apartment Building, South China University of Technology, Wushan RD., TianHe District, Guangzhou, 510640, China; 6 BGI-Forensics: Building 11, Beishan Industrial Zone, Yantian District, Shenzhen, 518083, China

This is a correction to:

GigaScience, Volume 6, Issue 9, 1 September 2017, gix066, https://doi.org/10.1093/gigascience/gix066

In the original version of the article “Population-wide sampling of retrotransposon insertion polymorphisms using deep sequencing and efficient detection,” by Qichao Yu et al. [1], the author names for reference 16 (Tellier LC) [2] and 50 [3] were incorrect.

Furthermore, the protocol DOIs for reference 52 [4] and 53 [5] were incorrect and did not resolve. The names and DOIs have been corrected, and the authors apologize for the error.
