# Supplementary material for: Population-wide sampling of retrotransposon insertion polymorphisms using deep sequencing and efficient detection
Source: Gigascience. 2017 Jul 31;6(9):1–11. doi: 10.1093/gigascience/gix066 (PMC5603766; doi:10.1093/gigascience/gix066)
Supplement: GIGA-D-16-00169_Original_Submission.pdf [file gix066_GIGA-D-16-00169_Original_Submission.pdf]

Manuscript Click here to download Manuscript Manuscript_SID.docx

Population-wide Sampling of Retroposon Insertion Polymorphisms Using Deep Sequencing and Efficient Detection

Qichao Yu[1,2,†], Wei Zhang[1,2,†], Xiaolong Zhang[2], Yongli Zeng[2], Yeming Wang[2], Yanhui Wang[2], Liqin Xu[2], Nannan Li[2], Xinlan Zhou[2], Xiaoyun Huang[2], Jie Lu[3], Xiaosen Guo[2], Guibo Li[2,4], Yong Hou[2,4], Shiping Liu[2,5,*] and Bo Li[2,6,*]

[1] BGI Education Center, University of Chinese Academy of Sciences, Shenzhen 518083, China

* Correspondence: libo@genomics.cn; liushiping@genomics.cn

[†] Equal contributors

Full list of author information is available at the end of the article.

Emails of all authors:

Qichao Yu: yuqichao@genomics.cn; Wei Zhang: zhangwei7@genomics.cn; Xiaolong Zhang: 13528497060@163.com; Yongli Zeng: zeoly100@163.com; Yeming Wang: 1398738509@qq.com; Yanhui Wang: 839584901@qq.com; Liqin Xu: xuliqin@genomics.cn; Nannan Li: linannan@genomics.cn; Xinlan Zhou: zhouxinlan@genomics.cn; Xiaoyun Huang: huangxiaoyun@genomics.cn; Jie Lu: lujie1@genomics.cn; Xiaosen Guo: guoxs@genomics.cn; Guibo Li: liguibo@genomics.cn; Yong Hou: houyong@genomics.cn; Bo Li: libo@genomics.cn; Shiping Liu: liushiping@genomics.cn.

Abstract

Background: Current conservative estimates suggest that almost half of the human genome is derived from transposable elements. In particular, active retrotransposons including Alu, L1, SVA and LTR have been known to be important during evolution and for shaping our genomes today, especially in genetic polymorphisms underlying a diverse set of diseases. Despite the obvious need for a thorough characterization of retrotransposon insertion polymorphisms (RIPs) in the general population, studies of human RIPs based on whole genome deep sequencing at the population level have not been undertaken sufficiently.

Findings: Herein we present a novel and efficient computational tool named SID for the detection of non-reference RIPs. We show that SID is suitable for high depth whole-genome sequencing data using paired-end reads through simulated and real datasets. We demonstrate that the time-efficiency and the accuracy of SID are superior to existing methods

Conclusions: The SID is a powerful and open-source program for detection of non-reference RIPs. Through our research on 90 Han Chinese individuals, we built a non-reference RIPs dataset that greatly enhanced the diversity of RIPs detected in the general population and, as such, should be invaluable to researchers interested in many aspects of human evolution, genetics, and disease.

Keywords: Transposable element, retrotransposon insertion polymorphism, next-generation sequencing, whole genome sequencing

Findings

Introduction

Transposable elements (TEs) are genomic sequences that can, either autonomously or in conjunction with other transposable elements, replicate within the genome, resulting in insertion polymorphisms. Over the evolutionary timescale, this process leads to drastic changes in genomic structure. Current estimates indicate that almost half of the human genome is derived from transposable elements [1]. Retrotransposons, which constitute ~93% of TEs [2], can be subdivided into those sequences containing Long Terminal Repeats (LTRs)

and those not (non-LTR). The majority of human TEs result from the present and past activity of non-LTR retrotransposons, including the L1 (long interspersed nuclear element 1), Alu and SVA elements, which collectively account for approximately one-third of the human genome [1].While most retrotransposons are inactive remnants prevalent among the human population, younger retrotransposons account for much of the structural variation among individual genomes [3]. There exist only a small proportion of total L1s which are highly active [4]. The current rate of retrotransposition in humans has been estimated as approximately 1 for every 20 births for Alu, approximately 1 for every 200 births for L1 and approximately 1 for every 900 births for SVA [5, 6].

Retrotransposon insertion is known as a disease causing mechanism [7], and the Next-generation sequencing (NGS) technology has been widely used to explore the association between retrotransposon insertions and disease, such as cancer [8-10]. In this respect, a comprehensive RIPs dataset of healthy population is necessary to serve as a reference to identify disease related RIPs. Based on the database of the 1000 Genomes Project (1000GP), researchers were able to carry out RIPs detection on an unprecedented scale through whole-genome sequencing and detect thousands of novel RIPs [11-13]. However, the 1000GP relied mainly on pooled low-coverage sequencing data (1~3× per individual) from many individuals for RIPs analysis. Because an insertion allele present in multiple individuals would effectively receive high coverage across the pooled dataset, this approach was biased towards common insertions. According to previous calculation, to detect heterozygous RIPs with high sensitivity using whole-genome sequencing, at least 30× coverage of sequencing was needed [14].

In addition, the current post-sequencing bioinformatic methods such as RetroSeq [11, 15] , TEA [10], PTEMD [16], Jitterbug [17], T-lex2 [18], Mobster[19] are challenged to deal with deep whole genome sequencing data especially at the population level because of time consumption. A fast and efficient method is required to detect RIPs in order to satisfy the increasing amount of whole genome sequencing (WGS) data.

Here we developed a new computer program named Specific Insertions Detector (SID) to detect RIPs, which has much higher detection efficiency but comparable detection accuracy and sensitivity compared with TEA and Retroseq, two of the most-cited algorithms of RIPs

calling. We next presented a non-reference TEs insertion polymorphism database by employing SID to analyze whole genome sequences of 90 Han Chinese individuals (YH90), acquired at a mean depth of 68×.

Materials and methods

Samples and whole genome sequencing

We obtained B-lymphocyte cell lines of 90 Han Chinese individuals from Coriell institute (Camden, New Jersey, USA). These samples were selected in Beijing, Hunan province and Fujian province respectively, and we broadly separated them into 'Northern group' (45 samples) and 'Southern group' (45 samples). DNA was extracted from the B-lymphocyte cell line of each individual and libraries were then constructed following the manufacturer's instructions, and high-coverage paired-end 100 bp WGS libraries were sequenced on the Illumina HiSeq 2000 Platform. In addition, we also used the Chinese sample [20] whose data has already been released in the European Nucleotide Archive (ENA) repository (for details see Additional file 1: Table S1). The study was approved by the Institutional Review Board on Bioethics and Biosafety of BGI (BGI-IRB).

Processing of the WGS data

Reads were aligned to human genome reference (HG19, Build37) using *BWA-0.6.1* [21] with parameters '-n 3 -o 1 -e 50'. Duplications were removed and the quality values of each reads were recalibrated using the Genome Analysis Toolkit (GATK) [22] and Samtools [23]. The resulting Binary Alignment/Map (BAM) files were required by SID.

The specific insertions detector pipeline

SID is compiled in Perl and includes two steps, discordant reads detection and reads clustering. Generally, the first step collects informative reads and generates other necessary files, while the second step discovers the specific insertion site and outputs the final results into a plain text.

Discordant reads detection. The ‘discordant reads’ are extracted for the subsequent clustering step. Paired-end reads were determined as ‘discordant reads’ if they met one of the following criteria: a. one read mapped to HG19 uniquely and the other read mapped to retrotransposons library (multi-mapped or unmapped to HG19); b. one read mapped to HG19 uniquely and the other soft-clipped read mapped to HG19, and the clipped sequence could be mapped to retrotransposons library; c. one soft-clipped read mapped to HG19, and the clipped sequence could be mapped to retrotransposons library, while the other read mapped to retrotransposons library (multi-mapped or unmapped to HG19) (Fig. 1a). This retrotransposons library includes the objective TE classes, such as L1, Alu, SVA, etc. In this study, the TE reference database contains known TE sequences collected from RepBase 17.07 [24], dbRIP [25] and Hot L1s [4]. In order to reduce the long processing time due to the large whole genome sequencing data, we implemented a parallel approach to process each bam files of samples simultaneously in the discordant reads detection step.

Reads clustering and breakpoints detection. First, the ‘discordant reads’ would be scanned and clustered into blocks which support potential RIPs based on Maximal Valid Clusters algorithm. Second, we exacted all reads located within the cluster regions and determined the breakpoints. Although high depth data enabled RIPs detection with high sensitivity because more soft-clipped reads neighboring target site duplication (TSD) could be detected, alignments neighboring the TSDs had apparently lower depth than the mean sequencing depth of whole genome due to some sequencing and system errors. This made the breakpoints detection difficult and increased the false positive rate inevitably. Thus, we added the recalibration process of clipped points to determine breakpoints. For each read that

located within the cluster regions around potential breakpoints was used to confirm the precise location of the breakpoints. Small deletions were extracted to perform breakpoints recalibration, and the mismatched bases were removed from the deletion sequences.

The clipped sequences were realigned to local regions on HG19 to gain the actual breakpoints. Breakpoints were taken as ‘clips’ if more than half of new clipped sequences were discordant to the reference sequence and the length of gap within new clipped sequence was less than 30%. The point would not be candidate unless it’s a ‘clip’ and the mismatch is less than 5 bp or contains polyA/T.

Some terminals of reads (Fig. 1b, c) that contain mismatched bases may be the clipped parts because the alignment software usually treats these bases as mismatches rather than clips. SID re-estimates the breakpoints candidates if mismatches were more than half of the read terminals.

Of note, we implemented ‘Asynchronism Scanning’ algorithm. Using this algorithm, once the program clustered one possible insertion region by scanning unique reads, the process of breakpoints detection in this region was carried out immediately, rendering it possible to detect RIPs in one chromosome in just few minutes. The detailed algorithm for RIP candidate determination is provided in Additional file 2: Supplementary Methods.

RIPs data simulation

In total 761 TEs were randomly selected from a retrotransposon database (homebrew from dbRIP) and inserted into HG19 autosomes randomly to generate a new human genome. The pIRS [26] software was used to generate about 60× paired-end reads and then we mapped these reads to HG19 genome by BWA. After that we used SID to detect these RIPs in the

simulated genome. By repeating this process, we got results in different depth simulated data to assess the sensitivity and specificity RIPs detection in sequence data with distinct depth using SID.

Subfamily, length and orientation annotation of the inserted retrotransposons

Subfamily annotation of RIPs was performed according to known active retrotransposons. We first constructed a comprehensive retrotransposons sequence library: Alu subfamily consensus sequences were acquired from RepBase 17.07 [24], L1 subfamily consensus sequences were acquired from Eunjung Lee [10], SVA and LTR consensus sequences were acquired from Baillie [27]. Next, we did the multiple subfamily sequence alignment of each type of retrotransposon and discovered the diagnosis position of each subfamily (for details see Additional file 1: Table S2-4). To ensure the complete diagnosis positions in each subfamily sequence, we used Ns to fill the gaps of each subfamily sequence that did not harbor the diagnosis positions sites. Specially, we discovered the diagnosis position of L1 from previous studies [28-31].

We then assembled the ‘discordant reads’ of each RIP into contigs using CAP3 [32] and realigned them against all of the subfamily sequences by BLAST [33]. We determined the maximum similarity score (S_{MS}) for each subfamily based on a simple penalty algorithm as following:

$$S_{MS} = \sum S_i$$

where S_i indicates the score of the specific diagnosis position i. $S_i = 1$ when the query genotype was same as the diagnosis position i of this subfamily; $S_i = -0.5$ when the position of query contigs were a gap while the diagnosis position i was not ‘N’, or the query

contigs were mismatch against the diagnosis position i.

We also determined divergence value (V_D) for each subfamily as following:

$$V_D = \frac{N_{mis} + N_{gap}}{L_{map}}$$

where N_{mis} and N_{gap} indicated mismatched base number and gaps number of query contigs, respectively. The L_{map} stood for the mapped length of the certain subfamily.

Subfamily with the maximum similarity based on the genotype of diagnosis position would be reported. If two or more subfamilies harbor the same maximum similarity, the subfamily with the smallest divergence value would be reported.

We treated the retrotransposon subfamily classification in dbRIP as 'golden control', and compared the classification result of 909 overlapped RIPs of our result and golden control, to evaluate the accuracy of the subfamily classification.

During the contigs mapping to subfamily sequences, we identified the first mapped site of 5' and 3' ends of the subfamily sequence, and accordingly counted the lengths from the initial site (L_{min} and L_{max}). The length of inserted retrotransposon (L_{retro}) was calculated as the difference between the maximum and the minimum length of aligned sequence:

$$L_{retro} = L_{max} – L_{min} + 1$$

The assembled contigs of both 5' and 3' ends of insertions had the same orientation of HG19 sequence, which we defined as 'positive orientation'. If the mapping orientations of the contigs were different, the orientation of RIPs was judged as the mapping orientation which most contigs supported. Also, the poly-A tail of retrotransposon would be annotated if the RIPs is 'positive' and there were more than four 'A' bases in the first 6 bases at 3' end of the contigs. And the poly-T tail the insertion orientation is 'negtive' and there were more than four 'T' bases

in the first 6 bases at 5’ end of the contigs.

Reference RIPs detection

The reference RIPs can be detected as a subset of deletions of the samples relative to the reference of HG19 (for details see Additional file 2: Figure S1). These deletions were selected from the structural variation (SV) detection result of YH90 samples (data not shown) and the RIPs can be annotated based on matching deletion coordinates to HG19 annotation of RepeatMasker (more than 90% overlap with each other) [34].

The reference RIPs should be absent in the chimpanzee genome. The alignments of chimpanzee mapped to human genome was downloaded from UCSC (http://hgdownload.cse.ucsc.edu). One reference RIP candidate should correspond to a gap with an overlap of more than 90% to each other, and there would be no gaps in the chimpanzee genome on this locus. The RIPs canditates would be filtered if there was no polymorphism in YH90 samples (allele frequecy equal to 180).

Results

Non-reference retrotransposon insertions calling

To detect non-reference RIPs from WGS data accurately and time-efficiently, we developed a computer program called Specific Insertions Detector (SID). Through discordant reads detection and reads clustering, it could detect non-reference RIPs easily and quickly (see Materials and Methods).

To investigate the influence of sequencing depth on RIPs detection sensitivity and accuracy, we simulated sequence data at different depth. It was observed that the detection sensitivity dramatically increased along with rising sequencing depth, and achieved 95% when

the sequencing depth was more than 30×. By contrast, the accuracy of detected RIPs had slight changes along with increasing sequencing depth (Fig. 2a).

We next estimated the RIPs detection sensitivity using two real sequencing datasets: One dataset was CEU trio data, which was deep-sequenced (>75×) Illumina HiSeq data generated at the Broad Institute (father NA12891, mother NA12892 and the female offspring NA12878) from the 1000GP. We first used SID to detect RIPs of each individual in CEU dataset (for details see Additional file 1: Table S5), and evaluated the sensitivity by comparing the detection results with the PCR-validated datasets from Stewart et al. [12]. For Alu, the mean sensitivity reached 96.3% among individuals. We also obtained mean sensitivity of 80.3% and 83.3% for L1 and SVA, respectively.

The other dataset including the NA18571, NA18572 and NA18537 were also recruited in 1000GP. The RIPs datasets of these three individuals detected by SID were much larger and covered 89.59% of the same sample's results in 1000GP on average (for details see Additional file 2: Figure S2).

We estimated the RIPs detection accuracy using the sequencing data of Asian individual lymphocytic cell line (YH_CL, ~52×) that was the first Asian diploid genome dataset, and performed the PCR validation straightly. We randomly selected 103 detected RIPs and 93/96 (7 loci were removed because of the poor primer specificity) loci were successfully validated, indicating that SID had an accuracy of 90.29% - 96.88% (for details see Additional file 1: Table S6). We also used the PCR validation result to access our genotyping accuracy. It was about 93.55% (87/93, Fig. 2b, for details see Additional file 2: Supplementary Methods).

Table 1. Run time for three different RIPs-detection programs.

Sample	Data size (GB)	Run time (h)		
		SID	TEA	RetroSeq
YH_CL	85	16.4	36.9	31.2
NA18571	127	19.9	90.6	110.3
NA18537	124	21.9	95.2	94.8
NA18572	117	31.9	82	99.8

We next compared the RIPs detection efficiency of different methods (SID, RetroSeq [11] and TEA [35]). In addition to YH_CL (Fig. 2c), we also selected three samples (NA18571, NA18572 and NA18537) from YH90, which were sequenced at an average depth of 67.91×. The run time of SID was about 3 times shorter than the other two methods, showing that the SID was the most time-saving method of these three (Table 1). SID and TEA had comparable sensitivity that was higher than RetroSeq, and the majority of SID detected RIPs (66.33% in average) existed in TEA's result, and an average of 16.87% SID detected RIPs could be generally detected by all three methods (Fig. 2c and Additional file 2: Figure S2). We also validated the uniquely detected RIPs by PCR, and gained an accuracy of 75.86% and 77.78% for Alu and L1, respectively, revealing a higher RIPs detection accuracy (Alu: 42.10% and 82.61%, L1: 66.67% and 66.67%, for RetroSeq and TEA, respectively).

A comprehensive RIPs landscape of human population

We then performed RIPs detection on a much larger scale. We sequenced 90 Han Chinese individuals and generated Illumina paired-end sequence data at an average depth of 68× for each sample (for details see Additional file 1: Table S1). The dataset included two groups in different regions of China, 45 samples from Northern China and 45 samples from Southern

China. Using the SID, the high depth of the dataset (much more than 30×) allowed us to build a comprehensive non-reference RIPs landscape with high confidence.

In total we identified 9342 non-reference RIPs in autosome regions, including 6483 Alu elements, 2398 L1s, 61 LTRs and 400 SVAs (Fig. 3a and for details see Additional file 1: Table S7). Of this dataset, 8433 RIPs including 5826 Alu elements, 2169 L1s, 383 SVAs, 55 LTRs were novel compared with dbRIP (Fig. 3b). The average number of non-reference RIPs per individual was 1394 (ranging from 1304 to 1493, Fig. 3c), including 1110.80 Alu elements, 231.34 L1s, 43.14 SVAs and 9.01 LTRs, respectively, and each type of RIPs had similar proportion (P = 0.6364, P = 0.2711, P = 0.2128, P = 0.5582, respectively, Wilcoxon signed-rank test). We compared pair-wise individuals of all 90 samples, and the average specific loci number was 672.79, almost a half (48.25%) of non-reference RIPs of one individual.

The specific inserted location information enabled us to investigate genome-wide sequence patterns of these non-reference RIPs. We observed that the non-reference RIPs varied between chromosomes (Fig. 3d, e). Of note, we found that the different two subpopulations (from Southern and Northern China respectively) had a similar pattern of RIPs distribution (r = 0.782, Fig. 3e and for details see Additional file 2: Figure S3). However, we did not find obvious correlation between the distribution of non-reference RIPs and GC content, fixed RIPs, as well as single nucleotide polymorphisms (SNPs) of the same sample within 10M non-N bins (Additional file 2: Figure S4).

To further investigate the distribution of non-reference RIPs in functional region, we annotated all of the inserted loci (Fig. 3f). More than half of RIPs (4828/9324) were located in

gene regions, and the majority of these in introns. Only 5/9324 RIPs were located in protein coding regions, including three genes C1orf66 (Alu inserted), SNX31 (Alu inserted) and APH1B (SVA inserted) with rare frequency (1/90), and two genes ADORA3 (Alu inserted) and Slco1b3 (L1 inserted) with higher frequency (44/90 and 12/90, respectively). Compared with all known genes, we noticed that RIPs inserted genes generally had a relatively lower expression level, later replication time in the cell cycle, lower GC content and lower conservation (higher Ka/Ks) than the average level of all genes, respectively ($P < 0.001$, Wilcoxon Test; Fig. 4). Besides the gene regions，we also found that at average 9.78% and 4.93% RIPs were located in enhancer regions and promoter regions per sample, respectively (Fig. 3f).

Furthermore, we annotated the subfamily, the orientation and the sequence length of all detected inserted retrotransposons based on regional sequence assembly and remapping to the retrotransposon library. AluY sub-family constituted essentially all non-reference Alu insertions, in which AluYa5 and AluYb8 were mostly active (for details see Additional file 1: Table S7), supporting conclusions from previous studies[28, 36, 37]. L1 insertions were dominated by the sub-family of L1-Pre, which was also in line with previous report [28].

The orientation of one RIP is judged from the mapping orientation of contigs to retrotransposon reference and the existing of poly-A or poly-T tails of inserted sequence (for details see Additional file 1: Table S7). Previous studies reported that the gene-inserted RIP had a greater influence on gene expression if it was inserted on the same orientation with the target gene [2, 38]. However, we detected a comparable number of direct and reverse events (0.475 and 0.525, respectively), arguing against an obvious natural selection on the RIPs with consistent orientation with the inserted gene.

Along with subfamily and orientation annotation, we also calculated the length of each insertion sequence. We found that different types of RIP had different length distributions (Additional file 2: Figure S5). More than half of Alu elements (~70%) were full-length while the length of the L1 distributed more discretely. Most of L1s (> 80%) were fractured during the process of retrotransposon, which verified previous study [13].

RIPs reference of healthy people

The pure and comprehensive dataset of RIPs can be used as a baseline of healthy people for other disease-related research, especially in single-gene disease. The candidate disease-related retrotransposon insertions that were found in this dataset would be filtered. We explicitly measured the overlap between our dataset and the disease-related retrotransposon insertions data in dbRIP (http://dbrip.org) [39]. None of the insertion sites existed in our dataset, indicating the accuracy of the database. We also tested some data of cancer research. We tested the dataset of candidate cancer related somatic retrotransposon insertions which was strictly generated from 11 tumor types data of The Cancer Genome Atlas (TCGA) Pan-Cancer Project. None of overlapped RIPs were detected, whereas 43.36% germline retrotransposons were detected. According to the comparison of colon cancer specific data [9], we found two L1 insertions consistent with our dataset with frequency of 51/90 and 50/90. These two L1 insertions were germline retrotransposon insertions that were further validated by PCR validation in Solyom's research. We also tested the candidate of Hepatocellular Carcinoma specific insertions [8] and found one L1 insertion was also present in our dataset with frequency of 9/90. This site was finally validated as a germline insertion by PCR in that research. All of these indicated that our data provided a reference panel to wipe off

false positive insertions related to cancer.

Conclusions

In this paper, we developed a computer program SID to detect non-reference RIPs of 90 healthy Han Chinese individuals through high depth whole-genome sequencing. Compared with TEA and RetroSeq, the SID has the fastest detection speed as well as high sensitivity and accuracy. We described the landscape of RIPs distribution on population genomes, and annotated the subfamily, orientation, and length of RIPs. We demonstrated that the RIPs could be used as a normal baseline for retrotransposon related disease research.

To our knowledge, this dataset is the largest dataset for human by now. Compared with 1000GP result of the same samples, the majority (mean 69.68%) of RIPs in our dataset has not been previously observed, suggesting that our deep-sequenced data had much higher detection sensitivity than the low coverage ones. For example, it was reported that the serum ACE level was determined by the Alu insertion/deletion (I/D) polymorphism in the following order: DD > ID > II [40], and the D allele of ACE gene was found to be associated with essential hypertension in different populations [41-44]. We found that ACE gene harbored Alu insertion in the 15th intron with a frequency of 81/90 in our 90 Chinese genomes, compared with the much lower frequency (7/63) in CEPH individuals [12], which was supported by previous study [45]. To our surprise, during the analysis of retrotransposon insertions of ACE, we found that there was no RIPs of ACE in Han Chinese samples of 1000GP dataset, which was a high-frequency inserted gene in our RIPs data. ACE specific PCR validation (for details see Additional file 2: Figure S6) and privious study of ACE [46] indicated our result was in line

with the real situation. It can be seen that the enough depth of sequencing is very important to investigate RIPs frequency and our data is able to present a better result in line with the actual situation.The highly sensitive and accurate RIPs dataset gave us a perfect opportunity to perform fitness analysis of RIPs.

This dataset can be used to compare with others to give guidance to research the disease-causing mechanisms in particular population and successfully coalesced the insert time of a specific locus. This dataset can also be used as a standard to other RIPs research and can be a baseline to filter meaningless RIPs in the disease-causing retrotransposon research. Genome-wide Association Studies (GWAS) have proven their utility in identifying genomic variants associated with risk for many diseases. Unlike SNPs and copy number variations (CNVs) that were widely used in GWAS, RIPs, the major contributor to human variation, have always been overlooked. It is significant that this dataset provides a valuable source to do GWAS and collects more markers related to complex diseases.

The high cost of whole-genome sequencing at high depth is still the main limitation, preventing it from being widely used in TEs research. Furthermore, the large amount of data yielded by high depth WGS makes it difficult to undertake bioinformatic analysis. With the development of biotechnology (BT) and IT, this situation would be changed soon. However, it may not be solved in a short time.

The next step is to research RIPs on the transcriptome level. The impact of RIPs on gene expression is still unclear. Combining the genome and transcriptome would give us a comprehensive picture of the regulation of RIPs. In this way, we can further expound the position of the retrotransposon in the course of human evolution.

Availability and requirements

- Project name: SID
- Project home page: https://github.com/Jonathanyu2014/SID
- Operating system(s): Linux
- Programming language: Perl
- Other requirements: Perl 5.14 or higher
- License: Apache License 2.0
- Any restrictions to use by non-academics: None

Additional files

Additional file 1: Supporting data description and the results of RIPs calling. (XLSX 1797 kb)

Additional file 2: The method of RIP candidate determination and all the supplementary figures. (PDF 1095 kb)

Abbreviations

RIP, retrotransposon insertion polymorphism; TE, transposable element; LTR, long terminal repeat; L1, long interspersed nuclear element 1; WGS, whole genome sequencing; NGS, next-generation sequencing; SID, specific insertions detector; TSD, target site duplication;1000GP, 1000 Genomes Project; CNV, copy number variation; SNP, single nucleotide polymorphism; ENA, European Nucleotide Archive; GWAS, genome-wide association study.

Acknowledgments

We are grateful for Zengli Yan, Nan Li, Na Li and Runze Jiang for optimizing and testing the SID program. We thank Haoxiang Lin and Wenjuan Zhu for providing technical assistance to us. We acknowledge the support by the 1000 Genomes Project Consortium. This work was supported by the Shenzhen Municipal Government of China [JSGG20140702161347218] and [KQCX20150330171652450].

Availability of data and materials

The source code of SID is available in GitHub repository[47]. The human (Homo sapiens) reference genome sequence (HG19) and its annotation files were downloaded from UCSC Genome Bioinformatics (http://genome.ucsc.edu/). The raw sequence data of YH_CL from previous reports is available in ENA repository (accession number ERA000005) [48]. All the YH90 raw sequences have been released in ENA repository (accession number ERA496654).

Authors' contribution

BL, SL, YH initiated this project and reviewed the manuscript. QY, XZ, YZ drafted the manuscript. XH, JL polished up the manuscript. QY, WZ, XZ, YW performed the data analysis and drew the pictures. YZ, YW designed and developed the SID program. NL, XZ, GL conducted the experiment for sequencing. LX designed the primers and did the PCR validation. YH, BL, SL, XZ, XG contributed with fruitful discussions.

Competing interests

The authors declare that they have no competing interests.

Author details

[1] BGI Education Center, University of Chinese Academy of Sciences, Shenzhen 518083, China. [2] BGI-Shenzhen, Shenzhen 518083, China. [3] BGI College, Shenzhen 518083, China. [4] Department of Biology, University of Copenhagen, Copenhagen 1599, Denmark. [5] School of Life Sciences, Sun Yat-sen University, Guangzhou 510006, China. [6] BGI-Forensics, Shenzhen 518083, China.

Ethics, consent and permissions

This study was approved by BGI-IRB.

Consent to publish

Both BGI-IRB and participants involved consented to publish this research.

References

1. Lander ES, Linton LM, Birren B, Nusbaum C, Zody MC, Baldwin J, Devon K, Dewar K, Doyle M, FitzHugh W *et al*: **Initial sequencing and analysis of the human genome**. *Nature* 2001, **409**(6822):860-921.

2. Cordaux R, Batzer MA: **The impact of retrotransposons on human genome evolution**. *Nature reviews Genetics* 2009, **10**(10):691-703.

3. Kidd JM, Graves T, Newman TL, Fulton R, Hayden HS, Malig M, Kallicki J, Kaul R, Wilson RK, Eichler EE: **A human genome structural variation sequencing resource reveals**

insights into mutational mechanisms. *Cell* 2010, **143**(5):837-847.

4. Brouha B, Schustak J, Badge RM, Lutz-Prigge S, Farley AH, Moran JV, Kazazian HH, Jr.: **Hot L1s account for the bulk of retrotransposition in the human population**. *Proceedings of the National Academy of Sciences of the United States of America* 2003, **100**(9):5280-5285.

5. Xing J, Zhang Y, Han K, Salem AH, Sen SK, Huff CD, Zhou Q, Kirkness EF, Levy S, Batzer MA *et al*: **Mobile elements create structural variation: analysis of a complete human genome**. *Genome research* 2009, **19**(9):1516-1526.

6. Cordaux R, Hedges DJ, Herke SW, Batzer MA: **Estimating the retrotransposition rate of human Alu elements**. *Gene* 2006, **373**:134-137.

7. Hancks DC, Kazazian HH, Jr.: **Active human retrotransposons: variation and disease**. *Curr Opin Genet Dev* 2012, **22**(3):191-203.

8. Shukla R, Upton KR, Munoz-Lopez M, Gerhardt DJ, Fisher ME, Nguyen T, Brennan PM, Baillie JK, Collino A, Ghisletti S *et al*: **Endogenous retrotransposition activates oncogenic pathways in hepatocellular carcinoma**. *Cell* 2013, **153**(1):101-111.

9. Solyom S, Ewing AD, Rahrmann EP, Doucet T, Nelson HH, Burns MB, Harris RS, Sigmon DF, Casella A, Erlanger B *et al*: **Extensive somatic L1 retrotransposition in colorectal tumors**. *Genome research* 2012, **22**(12):2328-2338.

10. Lee E, Iskow R, Yang L, Gokcumen O, Haseley P, Luquette LJ 3rd, Lohr JG, Harris CC, Ding L, Wilson RK *et al*: **Landscape of somatic retrotransposition in human cancers**. *Science* 2012, **337**(6097):967-971.

11. Keane TM, Wong K, Adams DJ: **RetroSeq: transposable element discovery from next-generation sequencing data**. *Bioinformatics* 2013, **29**(3):389-390.

12. Stewart C, Kural D, Stromberg MP, Walker JA, Konkel MK, Stutz AM, Urban AE, Grubert F, Lam HY, Lee WP *et al*: **A comprehensive map of mobile element insertion polymorphisms in humans**. *PLoS Genet* 2011, **7**(8):e1002236.

13. Ewing AD, Kazazian HH, Jr.: **Whole-genome resequencing allows detection of many rare LINE-1 insertion alleles in humans**. *Genome research* 2011, **21**(6):985-990.

14. Xing J, Witherspoon DJ, Jorde LB: **Mobile element biology: new possibilities with high-throughput sequencing**. *Trends in genetics : TIG* 2013, **29**(5):280-289.

15. Wong K, Keane TM, Stalker J, Adams DJ: **Enhanced structural variant and breakpoint detection using SVMerge by integration of multiple detection methods and local assembly**. *Genome biology* 2010, **11**(12):R128.

16. Kang H, Zhu D, Lin R, Opiyo SO, Jiang N, Shiu SH, Wang GL: **A novel method for identifying polymorphic transposable elements via scanning of high-throughput short reads**. *DNA Res* 2016, **23**(3):241-251.

17. Henaff E, Zapata L, Casacuberta JM, Ossowski S: **Jitterbug: somatic and germline transposon insertion detection at single-nucleotide resolution**. *BMC genomics* 2015, **16**:768.

18. Fiston-Lavier AS, Barron MG, Petrov DA, Gonzalez J: **T-lex2: genotyping, frequency estimation and re-annotation of transposable elements using single or pooled next-generation sequencing data**. *Nucleic acids research* 2015, **43**(4):e22.

19. Thung DT, de Ligt J, Vissers LE, Steehouwer M, Kroon M, de Vries P, Slagboom EP, Ye K, Veltman JA, Hehir-Kwa JY: **Mobster: accurate detection of mobile element insertions in next generation sequencing data**. *Genome biology* 2014, **15**(10):488.

20. Wang J, Wang W, Li R, Li Y, Tian G, Goodman L, Fan W, Zhang J, Li J, Zhang J *et al*: **The diploid genome sequence of an Asian individual**. *Nature* 2008, **456**(7218):60-65.

21. Li H, Durbin R: **Fast and accurate short read alignment with Burrows-Wheeler transform**. *Bioinformatics* 2009, **25**(14):1754-1760.

22. McKenna A, Hanna M, Banks E, Sivachenko A, Cibulskis K, Kernytsky A, Garimella K, Altshuler D, Gabriel S, Daly M *et al*: **The Genome Analysis Toolkit: a MapReduce framework for analyzing next-generation DNA sequencing data**. *Genome research* 2010, **20**(9):1297-1303.

23. Li H, Handsaker B, Wysoker A, Fennell T, Ruan J, Homer N, Marth G, Abecasis G, Durbin R, Genome Project Data Processing S: **The Sequence Alignment/Map format and SAMtools**. *Bioinformatics* 2009, **25**(16):2078-2079.

24. Jurka J, Kapitonov VV, Pavlicek A, Klonowski P, Kohany O, Walichiewicz J: **Repbase Update, a database of eukaryotic repetitive elements**. *Cytogenet Genome Res* 2005, **110**(1-4):462-467.

25. Wang J, Song L, Grover D, Azrak S, Batzer MA, P L: **dbRIP: a highly integrated database of retrotransposon insertion polymorphisms in humans**. *Hum Mutat* 2006, **27**(4):323-329.

26. Hu X, Yuan J, Shi Y, Lu J, Liu B, Li Z, Chen Y, Mu D, Zhang H, Li N: **pIRS: Profile-based Illumina pair-end reads simulator**. *Bioinformatics* 2012, **28**(11):1533-1535.

27. Baillie JK, Barnett MW, Upton KR, Gerhardt DJ, Richmond TA, De Sapio F, Brennan PM, Rizzu P, Smith S, Fell M *et al*: **Somatic retrotransposition alters the genetic landscape of the human brain**. *Nature* 2011, **479**(7374):534-537.

28. Boissinot S, Chevret P, AV F: **L1 (LINE-1) retrotransposon evolution and amplification in**

recent human history. *Mol Biol Evol* 2000, **17**(6):915-928.

29. Dombroski BA, Mathias SL, Nanthakumar E, Scott AF, Jr KH: **Isolation of an active human transposable element**. *Science* 1991, **254**(5039):1805-1808.

30. Ovchinnikov I, Rubin A, GD S: **Tracing the LINEs of human evolution**. *Proceedings of the National Academy of Sciences of the United States of America* 2002, **99**(16):10522-10527.

31. Ovchinnikov I, Troxel AB, GD S: **Genomic characterization of recent human LINE-1 insertions: evidence supporting random insertion**. *Genome research* 2001, **11**(12):2050-2058.

32. Huang X, Madan A: **CAP3: A DNA sequence assembly program**. *Genome research* 1999, **9**(9):868-877.

33. Altschul SF, Gish W, Miller W, Myers EW, Lipman DJ: **Basic local alignment search tool**. *Journal of molecular biology* 1990, **215**(3):403-410.

34. Tarailo-Graovac M, Chen N: **Using RepeatMasker to identify repetitive elements in genomic sequences**. *Curr Protoc Bioinformatics* 2009, **Chapter 4**:Unit 4 10.

35. Lee E, Iskow R, Yang L, Gokcumen O, Haseley P, Luquette LJ, 3rd, Lohr JG, Harris CC, Ding L, Wilson RK *et al*: **Landscape of somatic retrotransposition in human cancers**. *Science* 2012, **337**(6097):967-971.

36. Hormozdiari F, Alkan C, Ventura M, Hajirasouliha I, Malig M, Hach F, Yorukoglu D, Dao P, Bakhshi M, Sahinalp SC *et al*: **Alu repeat discovery and characterization within human genomes**. *Genome research* 2011, **21**(6):840-849.

37. Batzer MA, Deininger PL: **Alu repeats and human genomic diversity**. *Nature reviews Genetics* 2002, **3**(5):370-379.

38. Burns KH, Boeke JD: **Human transposon tectonics**. *Cell* 2012, **149**(4):740-752.

39. Wang J, Song L, Grover D, Azrak S, Batzer MA, Liang P: **dbRIP: a highly integrated database of retrotransposon insertion polymorphisms in humans**. *Hum Mutat* 2006, **27**(4):323-329.

40. Rigat B, Hubert C, Alhenc-Gelas F, Cambien F, Corvol P, F S: **An insertion/deletion polymorphism in the angiotensin I-converting enzyme gene accounting for half the variance of serum enzyme levels**. *J Clin Invest* 1990, **86**(4):1343-1346.

41. Jeng JR, Harn HJ, Jeng CY, Yueh KC, SM S: **Angiotensin I converting enzyme gene polymorphism in Chinese patients with hypertension**. *Am J Hypertens* 1997, **10**(5Pt1):558-561.

42. Zee RY, Lou YK, Griffiths LR, BJ M: **Association of a polymorphism of the angiotensin I-converting enzyme gene with essential hypertension**. *Biochem Biophys Res Commun* 1992, **184**(1):9-15.

43. Asamoah A, Yanamandra K, Thurmon TF, Richter R, Green R, Lakin T, C M: **A deletion in the angiotensin converting enzyme (ACE) gene is common among African Americans with essential hypertension**. *Clin Chim Acta* 1996, **254**(1):41-46.

44. Duru K, Farrow S, Wang JM, Lockette W, T K: **Frequency of a deletion polymorphism in the gene for angiotensin converting enzyme is increased in African-Americans with hypertension**. *Am J Hypertens* 1994, **7**(8):759-762.

45. Anand SS, Yusuf S, Vuksan V, Devanesen S, Teo KK, Montague PA, Kelemen L, Yi C, Lonn E, Gerstein H *et al*: **Differences in risk factors, atherosclerosis, and cardiovascular disease between ethnic groups in Canada: the Study of Health Assessment and Risk in Ethnic**

groups (SHARE). *Lancet* 2000, **356**(9226):279-284.

46. Batzer MA, Stoneking M, Alegria-Hartman M, Bazan H, Kass DH, Shaikh TH, Novick GE, Ioannou PA, Scheer WD, RJ H: **African origin of human-specific polymorphic Alu insertions**. *Proceedings of the National Academy of Sciences of the United States of America* 1994, **91**(25):12288-12292.

47. Yu Q: **Specific Insertions Detector**. In: *Zenodo.* 2016.

48. Zong C, Lu S, Chapman AR, Xie XS: **Genome-wide detection of single-nucleotide and copy-number variations of a single human cell**. *Science* 2012, **338**(6114):1622-1626.

Figure legends

Fig. 1 The principle of retrotransposon insertions detection. (a) The SID schematic diagram for RIPs detection in genome. TSD: target site duplication. SID: Specific Insertions Detector. (b) A reads mapping example of predicted homozygous insertions. (c) A reads mapping example of predicted heterozygous insertions. In (b) and (c), the red bases indicate the mismatches and the sequences with orange background stand for the clipped part of the reads. The clipped reads come from one allele with inserted retrotransposons and the normal reads come from the other allele that same with the reference. The three reads with asterisk show no clipped part but with terminal mismatches, which also can support the breakpoint and have consistency with the clipped reads.

Fig. 2 Assessing the results of SID. (a) Detecting accuracy and sensitivity estimation along cumulating sequencing depth of simulated data. (b) RIPs genotyping of YH_CL. The validation

results by PCR were marked. HEE: estimated heterozygous site. HOE: estimated homozygous site. HEV: validated heterozygous site. HOV: validated homozygous site. The dash line shows the estimated boundary between heterozygous and heterozygous site. Note that some of the validated RIPs stand in the same locus in the plot figure (for details see Additional file 1: Table S6). (c) RIPs detection results of YH_CL by three different programs. Adjacent 100bp regions of RIPs were taken into consideration.

Fig. 3 Comprehensive landscape of non-reference RIPs of YH90. (a) Proportions of novel insertions found for each kind of retrotransposon. (b) Comparison of YH90 non-reference RIPs results with dbRIP. Adjacent 100bp regions of RIPs were taken into consideration. (c) TE distribution of each YH sample. (d) Box plots of non-reference RIPs distribution among autosomes. (e) TE frequency distribution among YH90 samples. Rings from outer to inner stand for Alu insertions frequency, L1 insertion frequency, SVA insertion frequency, LTR insertion frequency and cytobands structure, respectively. The inside frequency of rings stands for northern people’s insertion frequency and the outside ones stand for southern people’s. (f) The RIPs distribution in different functional region of genome.

Fig. 4 Nature of non-reference TE inserted genes. The retrotransposons inserted genes were compared with all annotated genes of UCSC in aspects of gene expression (a), replication time of cell cycle (b), GC content (c) and conservation (d). The conservation of genes is represented by Ka/Ks ratio, and the replication time ranges approximately from 100 (very early) to 1000 (very late).

Figure 1

Click here to download Figure Fig. 1.pdf

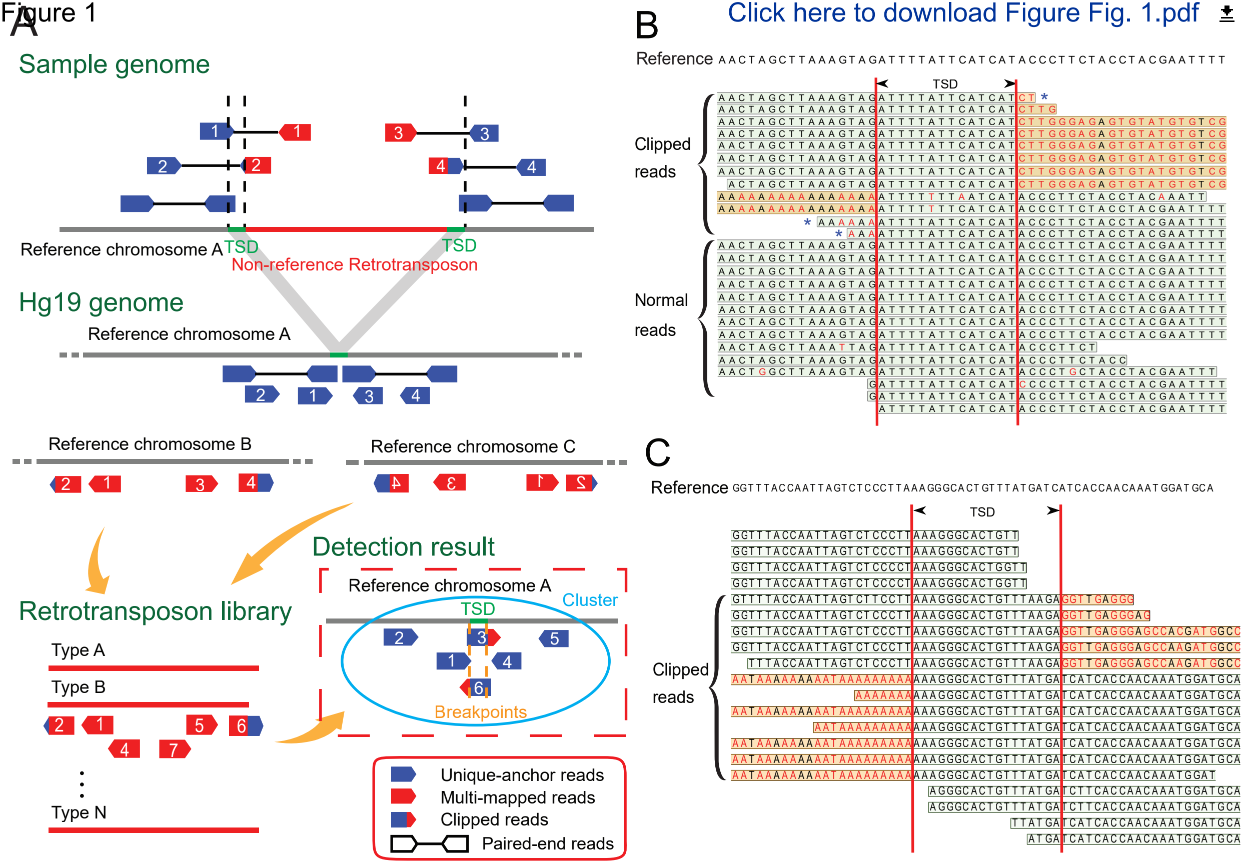

Figure 2

Click here to download Figure Fig.

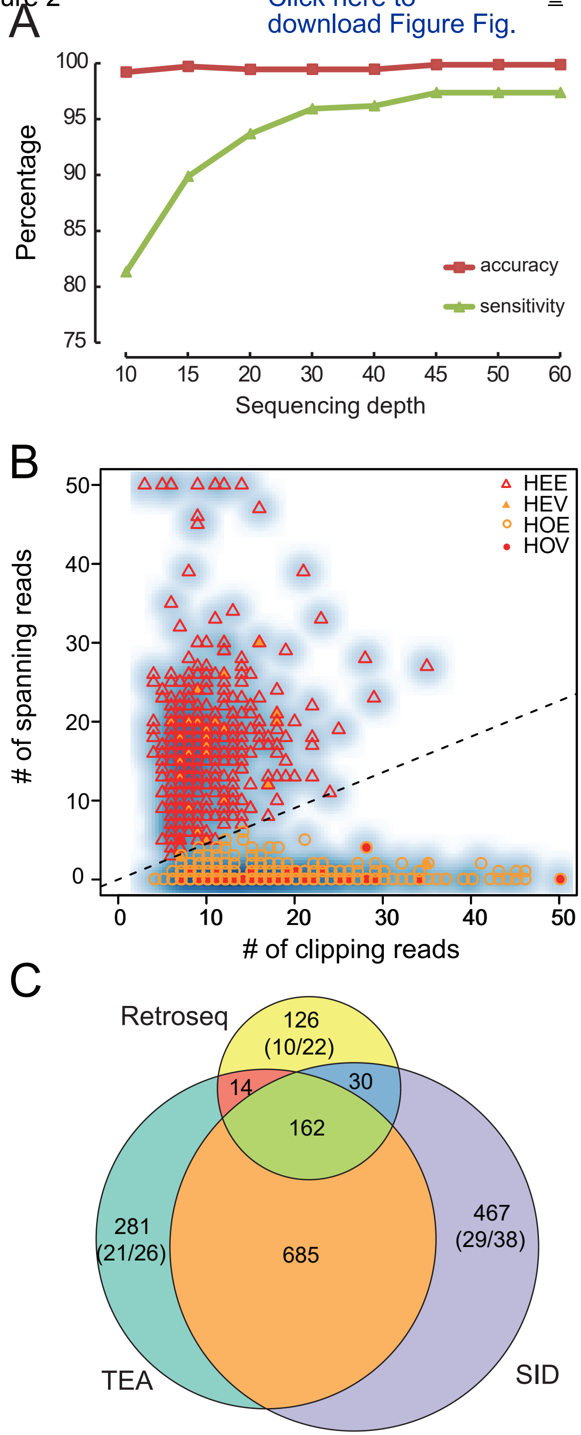

Figure 3

Click here to download Figure Fig. 3.pdf

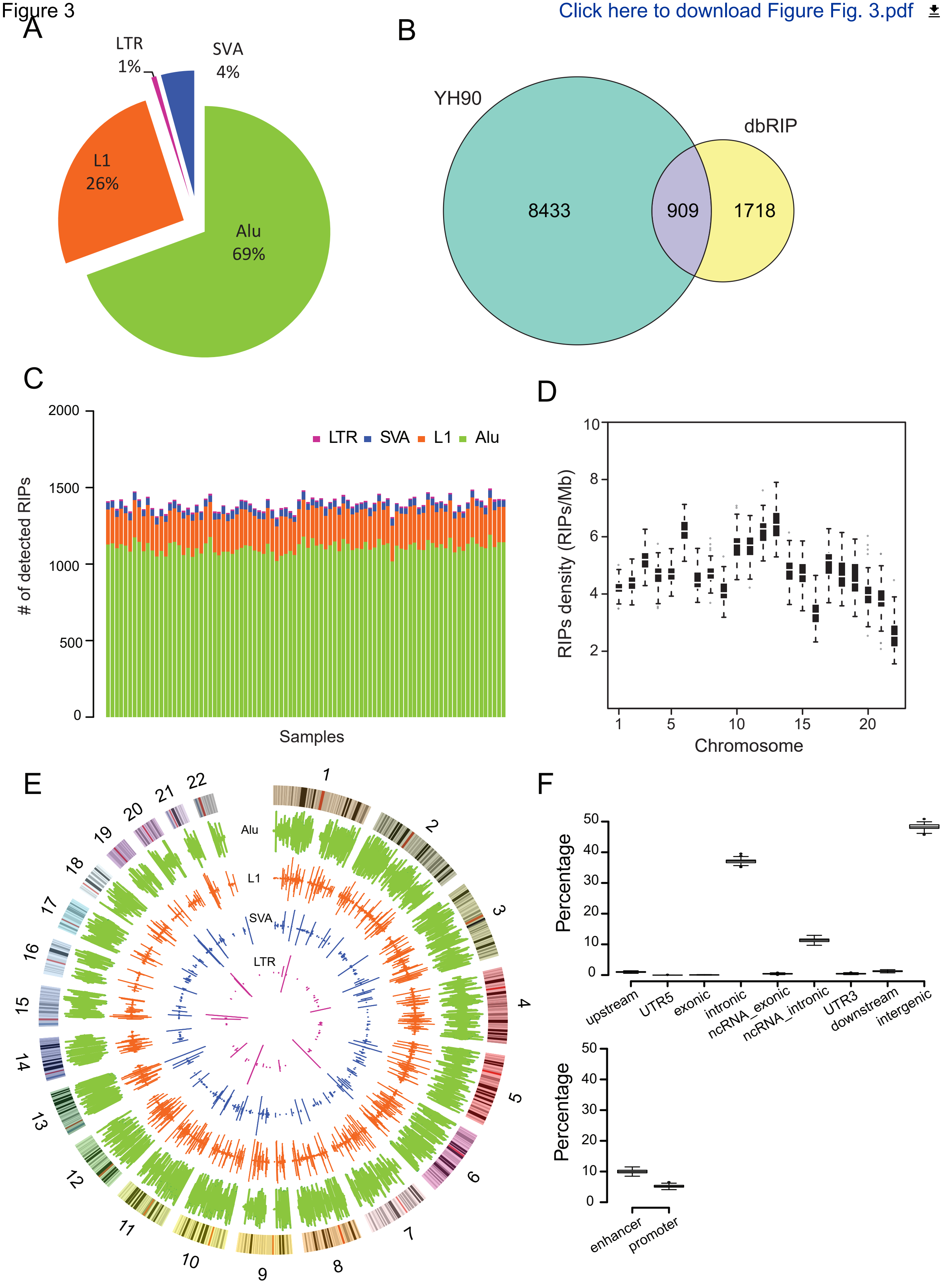

Figure 4

Click here to download Figure Fig. 4.pdf

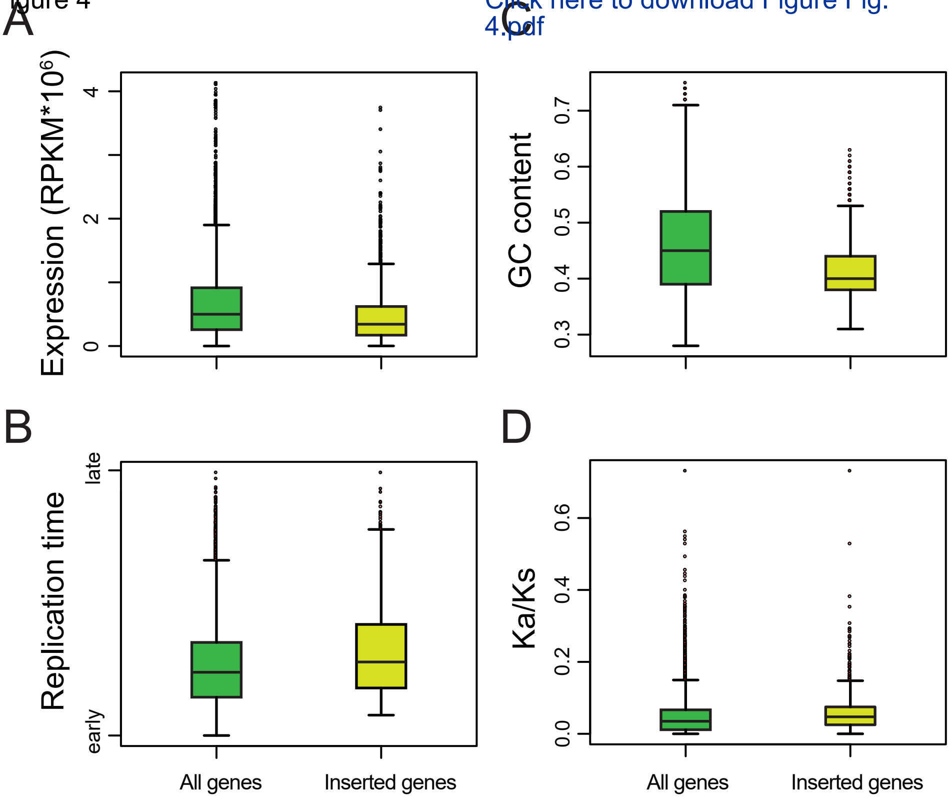

Click here to access/download
Supplementary Material
Additional file 1.xlsx

Click here to access/download
Supplementary Material
Additional file 2.pdf
