# Supplementary material for: Population-wide sampling of retrotransposon insertion polymorphisms using deep sequencing and efficient detection
Source: Gigascience. 2017 Jul 31;6(9):1–11. doi: 10.1093/gigascience/gix066 (PMC5603766; doi:10.1093/gigascience/gix066)
Supplement: GIGA-D-16-00169_Revision-2.pdf [file gix066_GIGA-D-16-00169_Revision-2.pdf]

Manuscript

Click here to download Manuscript TE manuscript 20170607_SCE.docx

Population-wide Sampling of Retrotransposon Insertion Polymorphisms Using Deep Sequencing and Efficient Detection

Qichao Yu[1,2,†], Wei Zhang[1,2,†], Xiaolong Zhang[2], Yongli Zeng[2], Yeming Wang[2], Yanhui Wang[2], Liqin Xu[2], Xiaoyun Huang[2], Nannan Li[2], Xinlan Zhou[2], Jie Lu[3], Xiaosen Guo[2], Guibo Li[2,4], Yong Hou[2,4], Shiping Liu[2,5,*] and Bo Li[2,6,*]

[1] BGI Education Center, University of Chinese Academy of Sciences, Shenzhen 518083, China

* Correspondence: libo@genomics.cn; liushiping@genomics.cn

[†] Equal contributors

Full list of author information is available at the end of the article.

Emails of all authors:

Qichao Yu: yuqichao@genomics.cn (ORCID: 0000-0003-2158-8424); Wei Zhang: zhangwei7@genomics.cn (ORCID: 0000-0002-5792-7662); Xiaolong Zhang: 13528497060@163.com; Yongli Zeng: zeoly100@163.com; Yeming Wang: 1398738509@qq.com (ORCID: 0000-0002-1521-2140); Yanhui Wang: 839584901@qq.com; Liqin Xu: xuliqin@genomics.cn; Nannan Li: linannan@genomics.cn (ORCID: 0000-0003-3632-7964); Xinlan Zhou: zhouxinlan@genomics.cn (ORCID: 0000-0001-9293-0894); Xiaoyun Huang: huangxiaoyun@genomics.cn (ORCID:

0000-0002-3389-9759); Jie Lu: lujie1@genomics.cn (ORCID: 0000-0001-7304-2023); Xiaosen Guo: guoxs@genomics.cn (ORCID: 0000-0003-1317-2760); Guibo Li: liguibo@genomics.cn (ORCID: 0000-0002-6141-4931); Yong Hou: houyong@genomics.cn (ORCID: 0000-0002-0420-0726); Bo Li: libo@genomics.cn; Shiping Liu: liushiping@genomics.cn (ORCID: 0000-0003-0019-619X).

Abstract

Background: Active retrotransposons play important roles during evolution and continue to shape our genomes today, especially in genetic polymorphisms underlying a diverse set of diseases. However, studies of human retrotransposon insertion polymorphisms (RIPs) based on whole-genome deep sequencing at the population level have not been sufficiently undertaken, despite the obvious need for a thorough characterization of RIPs in the general population.

Findings: Herein, we present a novel and efficient computational tool named Specific Insertions Detector (SID) for the detection of non-reference RIPs. We demonstrate that SID is suitable for high depth whole-genome sequencing (WGS) data using paired-end reads obtained from simulated and real datasets. We construct a comprehensive RIP database using a large population of 90 Han Chinese individuals with a mean 68× depth per individual. In total, we identify 9342 recent RIPs, and 8433 of these RIPs are novel compared with dbRIP, including 5826 Alu, 2169 long interspersed nuclear element 1 (L1), 383 SVA, and 55 long terminal repeats (LTR). Among the 9342 RIPs, 4828 were located in gene regions and five were located in protein-coding regions. We demonstrate that RIPs can, in principle, be an informative resource to perform population evolution and phylogenetic analyses. Taking the demographic effects into account, we identify a weak negative selection on SVA and L1 but approximately neutral selection for Alu elements based on the frequency spectrum of RIPs.

Conclusions: SID is a powerful open-source program for the detection of non-reference RIPs. We built a non-reference RIP dataset that greatly enhanced the diversity of RIPs detected in the general population and should be invaluable to researchers interested in many aspects of

human evolution, genetics, and disease. As a proof-of-concept, we demonstrate that the RIPs can be used as biomarkers in a similar way as single nucleotide polymorphisms (SNPs).

Keywords: Transposable element, retrotransposon insertion polymorphism, next-generation sequencing, whole-genome sequencing

Findings

Introduction

Transposable elements (TEs) are genomic sequences that can replicate within the genome either autonomously or in conjunction with other TEs, resulting in insertion polymorphisms. Over the evolutionary timescale, this process leads to drastic changes in genomic structure. Current estimates suggest that approximately half of the human genome is derived from TEs [1]. Retrotransposons, which constitute ~93% of TEs [2], can be subdivided into those sequences containing LTRs and those that do not (non-LTR). The majority of human TEs result from the activity of non-LTR retrotransposons, including the L1, Alu and SVA elements, which collectively account for approximately one-third of the human genome [1]. Although most retrotransposons are inactive remnants prevalent among the human population, younger retrotransposons account for much of the structural variation among individual genomes [3]. Only a small proportion of total L1s are highly active [4]. The current rate of retrotransposition in humans has been approximately estimated as 1 for every 20 births for Alu, 1 for every 200 births for L1 and 1 for every 900 births for SVA [5, 6].

Retrotransposon insertion is a disease-causing mechanism [7], and next-generation sequencing (NGS) technology has been widely used to explore the association between retrotransposon insertions and disease, such as cancer [8-10]. In this respect, a comprehensive RIP dataset of a healthy population is necessary to serve as a reference for the identification of disease-related RIPs. Based on the database of the 1000 Genomes Project (1000GP), researchers performed RIP detection on an unprecedented scale and detected thousands of novel RIPs [11-14]. This finding implies that an insertion allele present in multiple individuals would effectively receive high coverage across the pooled dataset,

leading to a detection bias toward common insertions. It was previously estimated that at least 30× coverage of sequencing is needed to detect heterozygous RIPs with high sensitivity using WGS [15].

Here, we developed the software SID to detect RIPs, which fulfilled our needs regarding detection efficiency, accuracy and sensitivity. We also generated a non-reference TE insertion polymorphism database by employing SID to analyze the whole-genome sequences of 90 Han Chinese individuals (YH90) acquired at a mean depth of 68×.

Materials and methods

Samples and whole genome sequencing

We obtained B-lymphocyte cell lines from 90 Han Chinese individuals at the Coriell Institute (Camden, New Jersey, USA). These individuals were selected from Beijing, Hunan province and Fujian province, respectively. We broadly separated the samples into “Northern group” (45 samples) and “Southern group” (45 samples). DNA was extracted from the B-lymphocyte cells of each individual, and libraries were then constructed following the manufacturer’s instructions. High-coverage paired-end 100 bp WGS libraries were sequenced on the Illumina HiSeq 2000 Platform. For more on this dataset see the Data Note describing its production published alongside this paper [16]. In addition, we also used a Chinese sample [17] for which the data were previously released in the European Nucleotide Archive (ENA) repository (Additional file 1: Table S1). The Institutional Review Board on Bioethics and Biosafety of BGI (BGI-IRB) approved the study.

Processing of the WGS data

Reads were aligned to the human genome reference (HG19, Build37) using *BWA* (BWA , RRID:SCR_010910)[18]. Duplications were removed using Picard tools, and the quality values of each reads were recalibrated using the Genome Analysis Toolkit (GATK)(GATK , RRID:SCR_001876)[19]. The resulting Binary Alignment/Map (BAM) files were used as input for SID (Additional file 2: Text S1).

The specific insertion detector pipeline

SID is compiled in Perl and includes the following two steps: discordant reads detection and reads clustering. Generally, the first step collects informative reads and generates other necessary files, whereas the second step discovers the specific insertion sites and exports the final results into plain text.

Detection of discordant reads. The “discordant reads” were extracted for the subsequent clustering step. Paired-end reads were determined as “discordant reads” if they met one of the following criteria: a. one read mapped to HG19 uniquely and the other read mapped to the retrotransposon library (multi-mapped or unmapped to HG19); b. one read mapped to HG19 uniquely and the other soft-clipped read mapped to HG19, and the clipped sequence could be mapped to the retrotransposon library; c. one soft-clipped read mapped to HG19, and the clipped sequence could be mapped to the retrotransposon library. The other read mapped to the retrotransposon library (multi-mapped or unmapped to HG19). The retrotransposon library includes objective TE classes, such as L1, Alu, and SVA. In this study, the TE reference database contains known TE sequences collected from RepBase version 17.07 [20], dbRIP [21] and Hot L1s [4]. To reduce the long processing time due to large volumes of WGS data, we implemented a parallel approach to process each bam files of samples simultaneously in the discordant reads detection step.

Reads clustering and detection of breakpoints. First, the “discordant reads” were scanned and clustered into blocks that supported potential RIPs based on the Maximal Valid Clusters algorithm [22]. Second, we extracted all reads located within the cluster regions and determined the breakpoints. Although high-depth, data-enabled RIP detection with high sensitivity was possible given that more soft-clipped reads neighboring target site duplication

(TSD) could be detected, alignments neighboring the TSDs had apparently lower depth compared with the mean sequencing depth of the whole genome due to occasional sequencing and system errors. This feature made breakpoint detection difficult and increased the false discovery rate (FDR). Thus, we added the recalibration process of clipped points to determine breakpoints. Each read located within the cluster regions flanking potential breakpoints was used to confirm the precise location of the breakpoints. Small deletions were extracted to perform breakpoint recalibration, and the mismatched bases were removed from the deletion sequences.

The clipped sequences were realigned to local regions on HG19 to determine the actual breakpoints. Breakpoints were assigned as “clips” if greater than half of the new clipped sequences were discordant with the reference sequence and the length of gap within the new clipped sequence was less than 30%. The point would not be a candidate unless it was a “clip” and the mismatch was less than 5 bp or contained poly-A/T.

Some terminals of reads containing mismatched bases may be the clipped parts because these bases were treated as mismatches rather than clips. The breakpoints candidates were re-estimated by SID if mismatches accounted for greater than half of the read terminals.

Notably, we implemented the “Asynchronous Scanning” algorithm (Additional file 2: Text S2). Using this algorithm, once the program clustered one possible insertion region by scanning unique reads, the process of breakpoint detection in this region was immediately performed, rendering it possible to detect TE insertions in one chromosome in only a few minutes. The detailed algorithm for RIP candidate determination is provided in Additional file 2: Text S2.

Annotation of TE insertions

Orientation annotation for the TE insertions. We annotated the orientation of TE insertions based on the BLAST results [23]. First, we extracted the discordant repeat anchored mate (RAM) reads and clipped reads that supported the TE insertion and made the reads' orientations the same as HG19. Then, we realigned the supporting reads against the consensus sequences of known active retrotransposons to identify the mapped orientation in known active retrotransposons. The orientations of TE insertions were judged by the reads' orientation (for details see Additional file 2: Text S3). The accuracy of orientation annotation was assessed by comparing 396 matched insertions from dbRIP and 21 fully sequenced insertions from PCR validation experiments (Additional file 1: Table S2). In total, 326 insertions were verified, and the FDR of orientation annotation was 21.82%.

Subfamily annotation for RIPs. The subfamily annotation of RIPs was performed according to known active retrotransposons. We first constructed a comprehensive retrotransposons sequence library. Alu subfamily consensus sequences were acquired from RepBase 17.07 [20]. L1 subfamily consensus sequences were acquired from Eunjung Lee [10]. SVA and LTR consensus sequences were acquired from Baillie [24]. Next, we performed multiple subfamily sequence alignment for each type of retrotransposon and discovered the diagnostic nucleotide for each subfamily (for details see Additional file 1: Table S3-5). Specially, we discovered the diagnostic nucleotide of L1 from previous studies [25-28]. We then assembled the "discordant reads" of each RIP into contigs using CAP3 [29] and realigned them against all of the subfamily sequences using BLAST (NCBI BLAST , RRID:SCR_004870)[30] (Additional file 2: Text S3-4).

Length annotation for RIPs. During mapping the contigs to subfamily sequences, we identified the first mapped site of the 5' and 3' ends of the subfamily sequence and accordingly counted the lengths from the initial site (L_{min} and L_{max}). The length of inserted retrotransposon (L_{retro}) was calculated as the difference between the maximum and the minimum length of the aligned sequence, as follows:

$$L_{retro} = L_{max} - L_{min} + 1.$$

Simulation of RIP data

In total, 761 TEs were randomly selected from our reference TE database (see Materials and methods: Annotation of TE insertions) and inserted into HG19 autosomes randomly to generate a new human genome (for details see Additional file 1: Table S6). The pIRS [31] software was used to generate approximately 60× paired-end 100 bp reads; then, we mapped these reads to the HG19 genome by BWA. Then, we used SID to detect these RIPs in the simulated genome. By repeating this process, we obtained results from simulated data with different depths to assess the sensitivity and specificity of RIP detection in sequence data with distinct depth using SID.

Reference RIP detection

The reference RIPs were detected as a subset of deletions of the samples relative to the HG19 reference (Additional file 2: Figure S1). These deletions were selected from the results of structural variation (SV) detection of YH90 samples, and the RIPs were annotated based on matched deletion coordinates to HG19 annotation of RepeatMasker (greater than 90% of them overlap with each other) [32].

The reference RIPs should be absent in the chimpanzee genome. The alignments of

chimpanzee mapped to the human genome were downloaded from UCSC (http://hgdownload.cse.ucsc.edu). One reference RIP candidate should correspond to a gap with an overlap of greater than 90% to each other, and no gaps were present in the chimpanzee genome at this locus. The RIP candidates were filtered if no polymorphisms were present in the YH90 samples (i.e., the allele frequency was equal to 180).

Results

Establishment of SID

To detect non-reference RIPs from WGS data accurately and in a time-efficient manner, we developed SID, which can detect non-reference RIPs easily and quickly through discordant reads detection and reads clustering. In the first step, three types of informative discordant reads were selected for further analysis (Fig. 1a). Then, the reads that had mismatched bases at the terminals (Fig. 1b, 1c) were used for judging heterozygosity. The clipped reads were used to confirm the sequence of TSD and the precise insertion site of certain TEs.

Non-reference retrotransposon insertion calling

To investigate the influence of sequencing depth on RIP detection sensitivity and accuracy, we simulated sequence data at different depths. Detection sensitivity dramatically increased with increasing sequencing depth and achieved 95% (730/761) when the sequencing depth was greater than 30×. By contrast, detection accuracy slightly changed with increasing sequencing depth (Fig. 2a).

We next estimated the RIP detection sensitivity using two real sequencing datasets. One dataset was the CEU trio data, which was deep-sequenced (> 75×) Illumina HiSeq data generated by the Broad Institute (father NA12891, mother NA12892 and the female offspring

NA12878) from the 1000GP. We first used SID to detect the RIPs of each individual in the CEU dataset and evaluated the sensitivity by comparing the detection results with the PCR-validated datasets from Stewart et al. [12]. For Alu, the mean sensitivity reached 96.3% among individuals. We also obtained a mean sensitivity of 80.3% and 83.3% for L1 and SVA, respectively (Additional file 1: Table S7).

The other dataset, including NA18571, NA18572 and NA18537, was also recruited in 1000GP. The RIP datasets of these three individuals detected by SID were larger and covered 70.08% of the same sample's results in 1000GP on average (Additional file 2: Figure S2). We estimated RIP detection accuracy using the sequencing data from a lymphocytic cell line (YH_CL, ~52×) obtained from an Asian individual. These data represent the first Asian diploid genome dataset, and we performed PCR validation. We randomly selected 103 detected RIPs, and 93/96 (7 loci were removed because of the poor primer specificity) loci were successfully validated, indicating that SID had an accuracy of 90.29% - 96.88% (Additional file 1: Table S8 and Additional file 2: Figure S3 and Text S5). We also used the PCR validation result to access the accuracy of genotyping, which was approximately 93.55% (87/93, Fig. 2b, Additional file 2: Text S6).

We next compared the RIP detection efficiency of different methods (SID, RetroSeq [11] and TEA [10]) using YH_CL and three samples (NA18571, NA18572 and NA18537) from YH90 (Additional file 2: Text S7). The run time of SID was approximately 3-fold reduced compared with the other two methods, suggesting that SID was the most time-saving method among the three methods (Additional file 2: Table S9). SID and TEA had comparable sensitivities that were increased compared with RetroSeq (Additional file 2: Figure S4). We

also validated the uniquely detected RIPs by PCR (Additional file 1: Table S10) with an accuracy of 75.86% (22/29) and 77.78% (7/9) for Alu and L1, respectively, revealing a higher RIP detection accuracy (Alu: 42.10% (8/19) and 82.61% (19/23) and L1: 66.67% (2/3) and 66.67% (2/3) for RetroSeq and TEA, respectively).

A comprehensive RIP landscape of the Han Chinese population

We then performed RIP detection on a much larger scale. We sequenced 90 Han Chinese individuals and generated Illumina paired-end sequence data at an average depth of 68× for each sample (Additional file 1: Table S1). Using SID, the high depth of the dataset (much more than 30×) allowed us to build a comprehensive non-reference RIP landscape with high confidence[16].

In total, we identified 9342 non-reference RIPs in autosome regions, including 6483 Alu elements, 2398 L1s, 61 LTRs and 400 SVAs (Fig. 3a; for details, see Additional file 1: Table S11 and Additional file 2: Text S8). Of this dataset, 8433 RIPs, including 5826 Alu elements, 2169 L1s, 383 SVAs, and 55 LTRs, were novel compared with dbRIP (Fig. 3b). The average number of non-reference RIPs per individual was 1394 (ranging from 1304 to 1493, Fig. 3c), including 1110.80 Alu elements, 231.34 L1s, 43.14 SVAs and 9.01 LTRs, and each type of RIP had a similar proportion (P = 0.6364, P = 0.2711, P = 0.2128, P = 0.5582, respectively, Wilcoxon signed-rank test). We compared pair-wise individuals of all 90 samples, and the average specific loci number was 672.79, which is approximately half (48.25%) of non-reference RIPs of one individual.

We next compared our results with the 1000GP SV dataset. In total, 34.94% (3264/9342) of RIPs in YH90 were also found in the 1000GP dataset. The Pearson correlation coefficient

was 0.7998 ($P < 2.2 \times 10^{-16}$) between YH90 and all the 26 populations in 1000GP SV dataset. The Pearson correlation coefficient was 0.8856 between YH90 and the East Asian (EAS) population in 1000GP, which was higher than other populations (r = 0.7662, r = 0.5741, r = 0.7025 and r = 0.7627 for American (AMR), African (AFR), European (EUR) and South Asian (SAS) populations, respectively. Additional file 2: Text S9)[14].

Specific insert location information enabled us to investigate genome-wide sequence patterns of these non-reference RIPs. We observed that the non-reference RIPs varied among chromosomes (Fig. 3d, e). Notably, we found that the two different subpopulations (from southern and northern China) had similar patterns of RIP distribution (r = 0.782, Fig. 3e and for details see Additional file 2: Figure S5). However, the distribution of non-reference RIPs was not obviously correlated with GC content, fixed RIPs, or SNPs of the same sample within 10M non-N bins (Additional file 2: Figure S6).

To further investigate the distribution of non-reference RIPs in the functional region, we annotated all the inserted loci (Fig. 3f). Greater than half of RIPs (4828/9342) were located in gene regions, and the majority of these were located in introns. Only 5/9342 RIPs were located in protein-coding regions, including three genes, C1orf66 (Alu-inserted), SNX31 (Alu-inserted) and APH1B (SVA-inserted), with low frequency (1/90) and two genes, ADORA3 (Alu-inserted) and Slco1b3 (L1-inserted), with higher frequency (44/90 and 12/90, respectively). In addition to gene regions, we also found that on average 9.78% and 4.93% RIPs were located in enhancer regions and promoter regions per sample, respectively (Fig. 3f).

Furthermore, we annotated the subfamily, orientation and sequence length of all detected inserted retrotransposons based on regional sequence assembly and remapping to the

retrotransposon library. The AluY sub-family constituted essentially all non-reference Alu insertions, in which AluYa5 and AluYb8 were mostly active (Additional file 1: Table S11), supporting conclusions from previous studies [26, 33, 34].

The orientation of one RIP is determined from the mapping orientation of contigs to a retrotransposon reference and the existence of poly-A or poly-T tails of the inserted sequence (Additional file 1: Table S11). Previous studies have reported that the gene-inserted RIP had a greater influence on gene expression if it was inserted on the same orientation as the target gene [2, 35]. However, we detected a comparable number of direct and reverse events (0.475 and 0.525, respectively), arguing against an obvious natural selection on the RIPs with consistent orientation with the inserted gene.

Along with subfamily and orientation annotation, we also calculated the length of each insertion sequence. We found that different types of TE insertions had different length distributions (Additional file 2: Figure S7). Greater than half of Alu elements (~70%) were full-length, whereas the length of the L1 was distributed more discretely. Most L1s (> 80%) were fractured during the process of retrotransposon, which is consistent with a previous study [13].

RIPs of a healthy population

The pure and comprehensive RIP dataset can be used as a baseline of healthy people for other disease-related research, especially single-gene diseases. The candidate disease-related retrotransposon insertions found in this dataset were filtered. We explicitly measured the overlap between our dataset and the disease-related retrotransposon insertion data in dbRIP (http://dbrip.org) [36]. None of the insertion sites existed in our dataset,

indicating the accuracy of the database. We also tested some cancer research data. We tested the dataset of candidate cancer-related somatic retrotransposon insertions that was strictly generated from data of The Cancer Genome Atlas (TCGA) Pan-Cancer Project for 11 tumor types. No overlapping RIPs were detected, whereas 43.36% germline retrotransposons were detected. According to the comparison of colon cancer-specific data [9], we identified two L1 insertions consistent with our dataset with frequency of 51/90 and 50/90. These two L1 insertions were germline retrotransposon insertions that were further validated by PCR validation in Solyom’s research. We also tested the candidate hepatocellular carcinoma-specific insertions [8] and identified one L1 insertion that was also present in our dataset with a frequency of 9/90. This site was finally validated as a germline insertion by PCR in that research. In conclusion, our data provide a reference panel to exclude false positive insertions related to cancer.

Population evolution analysis

To perform the population evolution analysis of RIPs, we first merged the non-reference RIP dataset with the “reference” retrotransposon insertions that were polymorphic in YH90 samples (Additional file 2: Figure S1) to obtain all RIPs from our samples. The retrotransposon insertions with a frequency equal to 1 were removed from our non-reference RIPs. The “reference” RIPs were defined as the reference genome-specific retrotransposon insertions compared with each individual of the YH90 group. These reference RIPs were selected from the dataset of YH90 deletions, and only the RIPs absent in chimpanzee were retained.

Allele frequency spectrum (AFS) was not only influenced by the natural selection but also by demographic history. For example, a low-frequency bias for the majority of mutations can

also be obtained if the population recently experienced a bottleneck [37].

To perform the neutral test more accurately, we took the demographic history into consideration (Additional file 2: Text S10). We simulated the following two different demographic scenarios: a two-epoch population with a recent contraction and a three-epoch bottleneck-shaped history containing a reduction of effective population size in the past followed by a recent phase of size recovery (Fig. 4a). We tested the different assumptions with the SNP dataset (Fig. 4b and Additional file 2: Table S12), which supported that the three-epoch model was the best model.

Next, we explored the possibility of using RIP information to perform population evolution analysis. Based on the genotyping result of the merged RIP dataset, we described the RIP AFS (Fig. 4c and Additional file 2: Text S11). The neutral model expectation can be calculated using the formula θ/i, where θ is the insertion diversity parameter and i (180) is the allele count in a fixed number of samples n (90) [37]. The spectrum was skewed toward low-allele frequency compared with the distribution of the expected neutral model, indicating possible negative selection pressure on retrotransposon insertions.

To investigate the influence of the demographic history on RIP AFS, we performed demographic correction and re-analyzed the RIP AFS under different selection models (Fig. 4d and Additional file 2: Figure S8-9). The classification of neutral with negative and positive selection indicates that a proportion of RIPs was neutral, and a proportion of RIPs was under negative selection. In addition, other RIPs were under positive selection (m1), neutral with negative selection (m2), neutral with positive selection (m3), negative selection (m4), positive selection (m5), and neutral selection (m6). We further calculated the selection coefficient (S')

under each best-fit model with the determination of an approximately neutral selection effect threshold (S' < 0.01%) [38]. Models m1 and m2 were the most fitted models with the observed RIP AFS (Additional file 2: Table S13). The best-fit result of model m1 demonstrated that approximately 75% RIPs were under negative selection with s = 0.0290%, which indicates that these RIPs are weakly deleterious. In addition, 10% were under positive selection, whereas 15% were neutral. Under model m2, the best-fit result demonstrated that 70% of RIPs were under negative selection with s = 0.0396%. In addition, 30% of RIPs were neutral. The selection coefficient was 0.0079% under the all negative selection model, indicating an approximately neutral selection effect.

The distribution of fitness effects of retrotransposon subfamilies (L1, SVA and Alu) was also estimated under the same demographic model. Assuming that all RIPs of different subfamilies were under negative selection (model m1), the selection coefficient models were various among three subfamilies of RIPs (S' = -0.0143%, S' = -0.0172%, S' = -0.0068% for L1, SVA and Alu, respectively), suggesting that there is more natural selection pressure on L1 and SVA (weakly negative selection) compared with Alu (nearly neutral selection).

Phylogenetic analysis

To investigate whether RIP information can be used to separate the Northern and Southern Chinese groups, we performed principal component analysis (PCA) using the RIPs detected from the YH90 dataset, which provided well-resolved Northern and Southern Chinese groups (Fig. 5a and Additional file 2: Text S12). Compared with the PCA result derived from the SNPs detected from the same dataset (Fig. 5b), there seemed to be more overlapping observations, indicating SNPs might be more informative in resolving the two distinctive populations. Next,

we determined whether it is possible to perform phylogenetic analysis using RIP information detected from the YH90 dataset. Two phylogenetic trees were constructed using RIPs and SNPs, separately (Fig. 5c and 5d; for details, see Additional file 2: Text S13). Similar to the PCA result, increased mixing between Northern and Southern Chinese individuals was observed for the phylogenetic tree derived from the RIP information. Interestingly, HG00534, an isolated Southern Chinese individual located in a northern cluster in the phylogenetic tree established using the SNP information, clustered largely with Southern Chinese individuals in the phylogenetic tree derived from the RIP information. Future studies are warranted to explore whether combining SNPs with RIP results in the construction of a more accurate phylogenetic tree.

Conclusions

In this paper, we developed the computer program SID to detect the non-reference RIPs of 90 healthy Han Chinese individuals using high-depth WGS. We described the landscape of RIP distribution on population genomes and annotated the subfamily, orientation, and length of RIPs. We demonstrated that the RIPs could be used as a normal baseline for retrotransposon-related disease research.

To our knowledge, this is the largest Han Chinese genomics dataset to date. Compared with 1000GP results from the same samples, approximately half (mean 48.05%; Additional file 2: Figure S2) of RIPs in our dataset were previously observed, suggesting that our deep-sequenced data exhibited increased detection sensitivity compared with low coverage data. For example, serum ACE levels were determined by the Alu insertion/deletion (I/D)

polymorphism in the following order: DD > ID > II [39]. The D allele of the ACE gene was associated with essential hypertension in different populations [40-43]. We found that the ACE gene harbored an Alu insertion in the 15th intron with a frequency of 81/90 in our 90 Chinese genomes compared with a considerably reduced frequency (7/63) in CEPH individuals [12], which was supported by a previous study [44]. To our surprise, no RIP ACEs were present in Han Chinese samples from the 1000GP dataset, which is a high-frequency inserted gene in our RIP data. ACE-specific PCR validation (Additional file 2: Figure S10) and a previous ACE study [45] indicated that our results were consistent with the real values. This finding suggests that adequate sequencing depth is important to investigate RIP frequency and that our data present a result that is consistent with the actual situation. The highly sensitive and accurate RIP dataset provided a perfect opportunity to perform RIP fitness analysis. This study evaluates the natural selection effect on retrotransposon insertions at the population level. As a type of long fragment insertion, RIPs are under approximately neutral selection. This finding is consistent with our result that retrotransposon insertions are mostly relatively inconsequential because the harbored genes are always relatively unimportant. Regarding different types of RIPs in addition to Alu, the longer insertion elements L1 and SVA exhibit weakly positive selection pressure.

This dataset can be compared with others to provide guidance in research of the disease-causing mechanisms in certain populations and to successfully determine the insertion time of a specific locus. This dataset can also be used as a standard for other RIP research and can serve as a baseline to filter irrelevant RIPs in disease-causing retrotransposon research. Genome-wide association studies (GWAS) have proven their utility

in identifying genomic variants associated with the risk for numerous diseases. Unlike SNPs and copy number variations (CNVs) that are widely used in GWAS, RIPs have generally been overlooked as a major contributor to human variation. Significantly, this dataset provides a valuable resource to perform GWAS and identify more markers related to complex diseases.

The high cost of WGS at high depth is still a major limitation, preventing it from being widely used in TE research. Furthermore, the large amount of data yielded by high-depth WGS makes it difficult to undertake bioinformatic analysis. With the development of biotechnology and IT, this situation should improve soon.

The next step is to research RIPs at the transcriptome level. The impact of RIPs on gene expression remains unclear. Combining the genome and transcriptome would provide a comprehensive picture about the regulation of RIPs. Thus, we can further expound the position of the retrotransposon in the course of human evolution.

Availability and requirements

- Project name: Specific Insertions Detector (SID)
- Project home page: https://github.com/Jonathanyu2014/SID
- Operating system(s): Linux
- Programming language: Perl
- Other requirements: Perl 5.14 or later, BLAST v2.2.25 or later, Samtools v1.0 or later
- License: Apache License 2.0
- Any restrictions to use by non-academics: None

Additional files

Additional file 1: Supplementary tables. Data description and the results of RIPs calling. (XLSX 1991 kb)

Additional file 2: Supplementary texts, figures and tables. (PDF 956 kb)

Abbreviations

CNV, copy number variation; ENA, European Nucleotide Archive; GWAS, genome-wide association study; LTR, long terminal repeat; L1, long interspersed nuclear element 1; NGS, next-generation sequencing; PCA, principal component analysis; RIP, retrotransposon insertion polymorphism; SID, specific insertions detector; SNP, single nucleotide polymorphism; TCGA, The Cancer Genome Atlas; TE, transposable element; TSD, target site duplication； WGS, whole-genome sequencing.

Acknowledgments

We are grateful for Zengli Yan, Nan Li, Na Li and Runze Jiang for optimizing and testing the SID program. We thank Haoxiang Lin and Wenjuan Zhu for providing technical assistance to us. We thank Liang Wu and Xulian Shi for polishing the manuscript. We acknowledge the support by the 1000 Genomes Project Consortium. This work was supported by the Shenzhen Municipal Government of China [JSGG20140702161347218] and [KQCX20150330171652450].

Availability of data and materials

The source code of SID is available from the GitHub and Zenodo repositories[46]. The human (Homo sapiens) reference genome sequence (HG19) and its annotation files were

downloaded from UCSC Genome Bioinformatics (http://genome.ucsc.edu/). The raw sequence data of YH_CL is available from the ENA repository (accession number ERA000005) [47]. All the YH90 raw sequences have been released to the ENA repository (bioproject number: PRJEB11005) and the processed data is also available from the *GigaScience* GigaDB repository [48]. Snapshots of the code, alignments, and results files are also hosted in GigaDB[49]. Protocols used for simulating reads for SNP Indel calling and detection of transportable element insertions are also hosted in the protocols.io repository[50, 51].

Authors' contributions

BL, SL and YH initiated this project and reviewed the manuscript. QY, XZ, YZ and XH drafted the manuscript. XH and JL edited the manuscript. QY, WZ, XZ and YW performed the data analysis and drew the pictures. YZ and YW designed and developed the SID program. NL, XZ and GL conducted the experiment for sequencing. LX designed the primers and performed PCR validation. YH, BL, SL, XZ, XG and XH provided fruitful discussions.

Competing interests

The authors declare that they have no competing interests.

Author details

[1] BGI Education Center, University of Chinese Academy of Sciences, Shenzhen 518083, China. [2] BGI-Shenzhen, Shenzhen 518083, China. [3] BGI College, Shenzhen 518083, China.

[4] Department of Biology, University of Copenhagen, Copenhagen 1599, Denmark.. [5] School of Biology and Biological Engineering, South China University of Technology, Guangzhou 510641, China. [6] BGI-Forensics, Shenzhen 518083, China.

Ethics, consent and permissions

This study was approved by BGI-IRB (NO. 16101).

Consent to publish

Both BGI-IRB and participants involved consented to publish this research.

References

1. Lander ES, Linton LM, Birren B, Nusbaum C, Zody MC, Baldwin J, et al. Initial sequencing and analysis of the human genome. Nature. 2001;409 6822:860-921.

2. Cordaux R and Batzer MA. The impact of retrotransposons on human genome evolution. Nature reviews Genetics. 2009;10 10:691-703. doi:10.1038/nrg2640.

3. Kidd JM, Graves T, Newman TL, Fulton R, Hayden HS, Malig M, et al. A human genome structural variation sequencing resource reveals insights into mutational mechanisms. Cell. 2010;143 5:837-47. doi:10.1016/j.cell.2010.10.027.

4. Brouha B, Schustak J, Badge RM, Lutz-Prigge S, Farley AH, Moran JV, et al. Hot L1s account for the bulk of retrotransposition in the human population. Proceedings of the National Academy of Sciences of the United States of America. 2003;100 9:5280-5. doi:10.1073/pnas.0831042100.

5. Xing J, Zhang Y, Han K, Salem AH, Sen SK, Huff CD, et al. Mobile elements create structural variation: analysis of a complete human genome. Genome research. 2009;19 9:1516-26. doi:10.1101/gr.091827.109.

6. Cordaux R, Hedges DJ, Herke SW and Batzer MA. Estimating the retrotransposition rate of human Alu elements. Gene. 2006;373:134-7. doi:10.1016/j.gene.2006.01.019.

7. Hancks DC and Kazazian HH, Jr. Active human retrotransposons: variation and disease. Curr Opin Genet Dev. 2012;22 3:191-203. doi:10.1016/j.gde.2012.02.006.

8. Shukla R, Upton KR, Munoz-Lopez M, Gerhardt DJ, Fisher ME, Nguyen T, et al. Endogenous retrotransposition activates oncogenic pathways in hepatocellular carcinoma. Cell. 2013;153 1:101-11. doi:10.1016/j.cell.2013.02.032.

9. Solyom S, Ewing AD, Rahrmann EP, Doucet T, Nelson HH, Burns MB, et al. Extensive somatic L1 retrotransposition in colorectal tumors. Genome research. 2012;22 12:2328-38. doi:10.1101/gr.145235.112.

10. Lee E, Iskow R, Yang L, Gokcumen O, Haseley P, Luquette LJ, 3rd, et al. Landscape of somatic retrotransposition in human cancers. Science. 2012;337 6097:967-71. doi:10.1126/science.1222077.

11. Keane TM, Wong K and Adams DJ. RetroSeq: transposable element discovery from next-generation sequencing data. Bioinformatics. 2013;29 3:389-90. doi:10.1093/bioinformatics/bts697.

12. Stewart C, Kural D, Stromberg MP, Walker JA, Konkel MK, Stutz AM, et al. A comprehensive map of mobile element insertion polymorphisms in humans. PLoS Genet. 2011;7 8:e1002236. doi:10.1371/journal.pgen.1002236.

13. Ewing AD and Kazazian HH, Jr. Whole-genome resequencing allows detection of many rare LINE-1 insertion alleles in humans. Genome research. 2011;21 6:985-90. doi:10.1101/gr.114777.110.

14. Sudmant PH, Rausch T, Gardner EJ, Handsaker RE, Abyzov A, Huddleston J, et al. An integrated map of structural variation in 2,504 human genomes. Nature. 2015;526 7571:75-81. doi:10.1038/nature15394.

15. Xing J, Witherspoon DJ and Jorde LB. Mobile element biology: new possibilities with high-throughput sequencing. Trends in genetics : TIG. 2013;29 5:280-9. doi:10.1016/j.tig.2012.12.002.

16. Lan, T; Lin, H; Asker Melchior Tellier, L, C; Zhu, W; Yang, M; Liu, X; Wang, J; Wang, J; Yang, H; Xu, X; Guo, X (2017): Deep whole-genome sequencing of 90 Han Chinese genomes. GigaScience. In Press.

17. Wang J, Wang W, Li R, Li Y, Tian G, Goodman L, et al. The diploid genome sequence of an Asian individual. Nature. 2008;456 7218:60-5. doi:10.1038/nature07484.

18. Li H and Durbin R. Fast and accurate short read alignment with Burrows-Wheeler transform. Bioinformatics. 2009;25 14:1754-60. doi:10.1093/bioinformatics/btp324.

19. McKenna A, Hanna M, Banks E, Sivachenko A, Cibulskis K, Kernytsky A, et al. The Genome Analysis Toolkit: a MapReduce framework for analyzing next-generation DNA sequencing data. Genome research. 2010;20 9:1297-303. doi:10.1101/gr.107524.110.

20. Jurka J, Kapitonov VV, Pavlicek A, Klonowski P, Kohany O and Walichiewicz J. Repbase Update, a database of eukaryotic repetitive elements. Cytogenet Genome Res. 2005;110 1-4:462-7. doi:10.1159/000084979.

21. Wang J, Song L, Grover D, Azrak S, Batzer MA and P L. dbRIP: a highly integrated database of retrotransposon insertion polymorphisms in humans. Hum Mutat. 2006;27 4:323-9.

22. Hormozdiari F, Hajirasouliha I, Dao P, Hach F, Yorukoglu D, Alkan C, et al. Next-generation VariationHunter: combinatorial algorithms for transposon insertion discovery. Bioinformatics. 2010;26 12:i350-7. doi:10.1093/bioinformatics/btq216.

23. Mount DW. Using the Basic Local Alignment Search Tool (BLAST). CSH Protoc. 2007;2007:pdb top17. doi:10.1101/pdb.top17.

24. Baillie JK, Barnett MW, Upton KR, Gerhardt DJ, Richmond TA, De Sapio F, et al. Somatic retrotransposition alters the genetic landscape of the human brain. Nature. 2011;479 7374:534-7. doi:10.1038/nature10531.

25. Boissinot S, Chevret P and AV F. L1 (LINE-1) retrotransposon evolution and amplification in recent human history. Mol Biol Evol. 2000;17 6:915-28.

26. Dombroski BA, Mathias SL, Nanthakumar E, Scott AF and Jr KH. Isolation of an active human transposable element. Science. 1991;254 5039:1805-8.

27. Ovchinnikov I, Rubin A and GD S. Tracing the LINEs of human evolution. Proceedings of the National Academy of Sciences of the United States of America. 2002;99 16:10522-7.

28. Ovchinnikov I, Troxel AB and GD S. Genomic characterization of recent human LINE-1 insertions: evidence supporting random insertion. Genome research. 2001;11 12:2050-8.

29. Huang X and Madan A. CAP3: A DNA sequence assembly program. Genome research. 1999;9 9:868-77.

30. Altschul SF, Gish W, Miller W, Myers EW and Lipman DJ. Basic local alignment search tool. Journal of molecular biology. 1990;215 3:403-10.

31. Hu X, Yuan J, Shi Y, Lu J, Liu B, Li Z, et al. pIRS: Profile-based Illumina pair-end reads simulator. Bioinformatics. 2012;28 11:1533-5.

32. Tarailo-Graovac M and Chen N. Using RepeatMasker to identify repetitive elements in genomic sequences. Curr Protoc Bioinformatics. 2009;Chapter 4:Unit 4 10. doi:10.1002/0471250953.bi0410s25.

33. Hormozdiari F, Alkan C, Ventura M, Hajirasouliha I, Malig M, Hach F, et al. Alu repeat discovery and characterization within human genomes. Genome research. 2011;21 6:840-9. doi:10.1101/gr.115956.110.

34. Batzer MA and Deininger PL. Alu repeats and human genomic diversity. Nature reviews Genetics. 2002;3 5:370-9. doi:10.1038/nrg798.

35. Burns KH and Boeke JD. Human transposon tectonics. Cell. 2012;149 4:740-52. doi:10.1016/j.cell.2012.04.019.

36. Wang J, Song L, Grover D, Azrak S, Batzer MA and Liang P. dbRIP: a highly integrated database of retrotransposon insertion polymorphisms in humans. Hum Mutat. 2006;27 4:323-9. doi:10.1002/humu.20307.

37. Tajima F. Statistical method for testing the neutral mutation hypothesis by DNA polymorphism. Genetics. 1989;123 3:585-95.

38. Boyko AR, Williamson SH, Indap AR, Degenhardt JD, Hernandez RD, Lohmueller KE, et al. Assessing the evolutionary impact of amino acid mutations in the human genome. PLoS Genet. 2008;4 5:e1000083. doi:10.1371/journal.pgen.1000083.

39. Rigat B, Hubert C, Alhenc-Gelas F, Cambien F, Corvol P and F S. An insertion/deletion polymorphism in the angiotensin I-converting enzyme gene accounting for half the variance of

serum enzyme levels. J Clin Invest. 1990;86 4:1343-6.

40. Jeng JR, Harn HJ, Jeng CY, Yueh KC and SM S. Angiotensin I converting enzyme gene polymorphism in Chinese patients with hypertension. Am J Hypertens. 1997;10 5Pt1:558-61.

41. Zee RY, Lou YK, Griffiths LR and BJ M. Association of a polymorphism of the angiotensin I-converting enzyme gene with essential hypertension. Biochem Biophys Res Commun. 1992;184 1:9-15.

42. Asamoah A, Yanamandra K, Thurmon TF, Richter R, Green R, Lakin T, et al. A deletion in the angiotensin converting enzyme (ACE) gene is common among African Americans with essential hypertension. Clin Chim Acta. 1996;254 1:41-6.

43. Duru K, Farrow S, Wang JM, Lockette W and T K. Frequency of a deletion polymorphism in the gene for angiotensin converting enzyme is increased in African-Americans with hypertension. Am J Hypertens. 1994;7 8:759-62.

44. Anand SS, Yusuf S, Vuksan V, Devanesen S, Teo KK, Montague PA, et al. Differences in risk factors, atherosclerosis, and cardiovascular disease between ethnic groups in Canada: the Study of Health Assessment and Risk in Ethnic groups (SHARE). Lancet. 2000;356 9226:279-84.

45. Batzer MA, Stoneking M, Alegria-Hartman M, Bazan H, Kass DH, Shaikh TH, et al. African origin of human-specific polymorphic Alu insertions. Proceedings of the National Academy of Sciences of the United States of America. 1994;91 25:12288-92.

46. Qichao Yu. (2016, September 1). Specific Insertions Detector. Zenodo. http://doi.org/10.5281/zenodo.212115

47. Zong C, Lu S, Chapman AR and Xie XS. Genome-wide detection of single-nucleotide and copy-number variations of a single human cell. Science. 2012;338 6114:1622-6.

doi:10.1126/science.1229164.

48. Lan, T; Lin, H; Asker Melchior Tellier, L, C; Zhu, W; Yang, M; Liu, X; Wang, J; Wang, J; Yang, H; Xu, X; Guo, X (2017): Supporting data for "Deep whole-genome sequencing of 90 Han Chinese genomes" GigaScience Database. http://dx.doi.org/10.5524/100302

49. Yu, Q; Zhang, W; Zeng, Y; Zhang, X; Wang, Y; Wang, Y; Xu, L; Huang, X; Li, N; Zhou, X; Lu, J; Guo, X; Li, G; Hou, Y; Liu, S; Li, B (2017): Supporting data for "Population-wide Sampling of Retrotransposon Insertion Polymorphisms Using Deep Sequencing and Efficient Detection" GigaScience Database. http://dx.doi.org/10.5524/100318

50 Haoxiang Lin: SNP INDEL calling. protocols.io dx.doi.org/10.17504/protocols.io.grkbv4w

51. GigaScience Database: Simulating reads for detection of transportable element insertions. protocols.io. 2017. dx.doi.org/10.17504/protocols.io.imrcc56

Figure legends

Fig. 1 The principle of retrotransposon insertion detection. (**a**) Schematic diagram of using SID for RIP detection in the genome. TSD: target site duplication. SID: Specific Insertions Detector. (**b**) An example of reads mapping for predicted homozygous insertions. (**c**) An example of reads mapping for predicted heterozygous insertions. In (**b**) and (**c**), the red bases indicate the mismatches, and the sequences with an orange background represent the clipped part of the reads. The clipped reads are derived from one allele with inserted retrotransposons, and the normal reads are derived from the other allele with the same reference. The three reads with asterisks indicate no clipped part but the presence of terminal mismatches, which can also

support the breakpoint and exhibit consistency with the clipped reads.

Fig. 2 Assessing the SID results. (**a**) Detecting accuracy and sensitivity estimation along cumulating sequencing depth of simulated data. (**b**) RIP genotyping of YH_CL. PCR validation results are marked. HEE: estimated heterozygous site. HOE: estimated homozygous site. HEV: validated heterozygous site. HOV: validated homozygous site. The dash line indicates the estimated boundary between heterozygous and heterozygous sites. Note that some of the validated RIPs are present in the same locus in the plot figure.

Fig. 3 Comprehensive landscape of non-reference RIPs of YH90. (**a**) Proportions of novel insertions identified for each type of retrotransposon. (**b**) Comparison of YH90 non-reference RIP results with dbRIP. Adjacent 100-bp regions of RIPs were taken into consideration. (**c**) TE distribution of each YH90 sample. (**d**) Box plots of non-reference RIP distribution among autosomes. (**e**) TE frequency distribution among YH90 samples. Rings from outer to inner indicate Alu insertions frequency, L1 insertion frequency, SVA insertion frequency, LTR insertion frequency and cytoband structure. The inside frequency of the rings indicates the insertion frequency for the Northern Chinese group, and the outside frequency represents that of the Southern Chinese group. (**f**) RIP distribution in different functional regions of the genome.

Fig. 4 Population genetics analysis based on YH90. (**a**) A two-epoch population with a recent contraction; a three-epoch bottleneck-shaped history, which contained a reduction of the effective population size in the past followed by a recent phase of size recovery. Details of the parameters for all models are provided in Additional file 2: Table S12. (**b**) The observed SNP

frequency spectra and expected neutral SNP frequency spectra under different demographic models. (**c**) Observed and expected RIP site frequency spectra before demographic correction of each subfamily. (**d**) Assessing the evolutionary impact of RIPs in the human genome. The allele frequency distribution of RIPs was compared among observed, neutral models and negative models after demographic correction.

Fig. 5 Phylogenetic analysis using RIPs and SNPs. (**a**) The detected RIPs were used for PCA. Each dot represents a sample from YH90 and is plotted as scatterplot using PC1 and PC2. Red indicates samples from individuals from northern China, and blue indicates individuals from southern China. (**b**) The detected SNPs were used for PCA. The plot layout and legend are the same as those presented in (**a**). (**c**) Phylogenetic tree constructed using the detected RIPs. HG19 (green) is used as a control. Red indicates samples from individuals from northern China, and blue indicates samples from individuals from southern China. (**d**) Phylogenetic tree constructed using the detected SNPs. HG19 (green) is used as a control. Plot layout and legend are same as that presented in (**c**).

Figure 1

Click here to download Figure Fig. 1.pdf

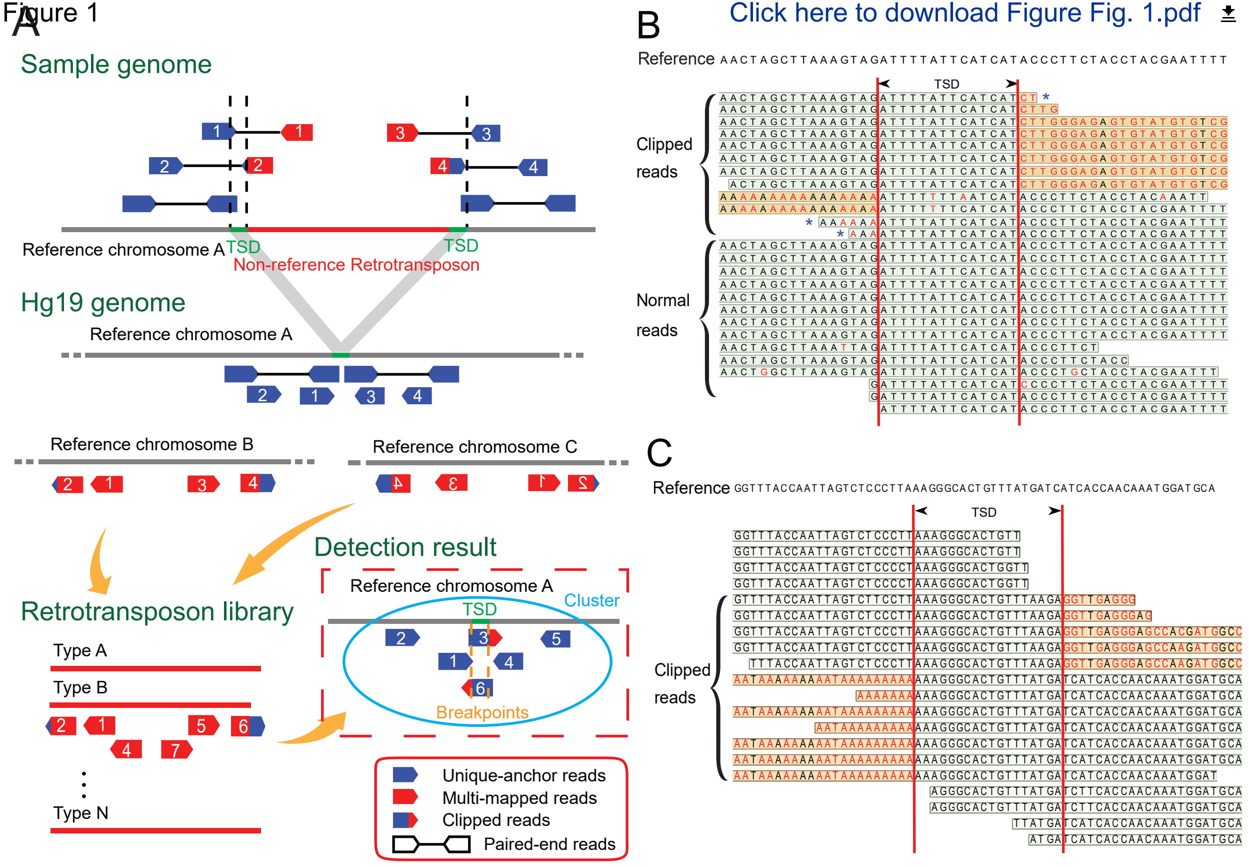

Figure 2

Click here to download Figure Fig. 2.pdf

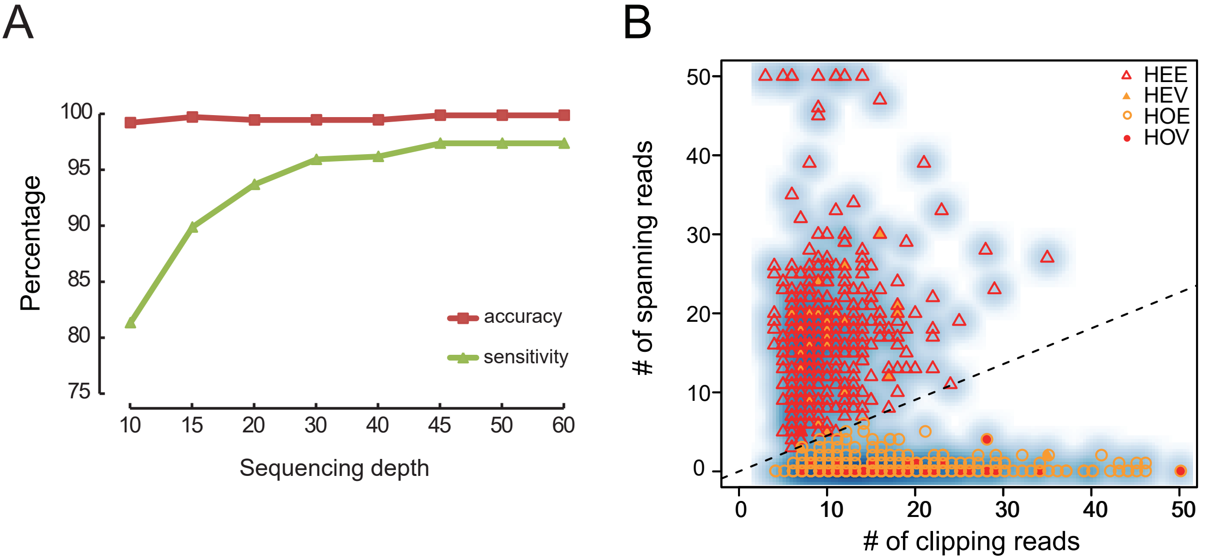

Figure 3

Click here to download Figure Fig. 3.pdf

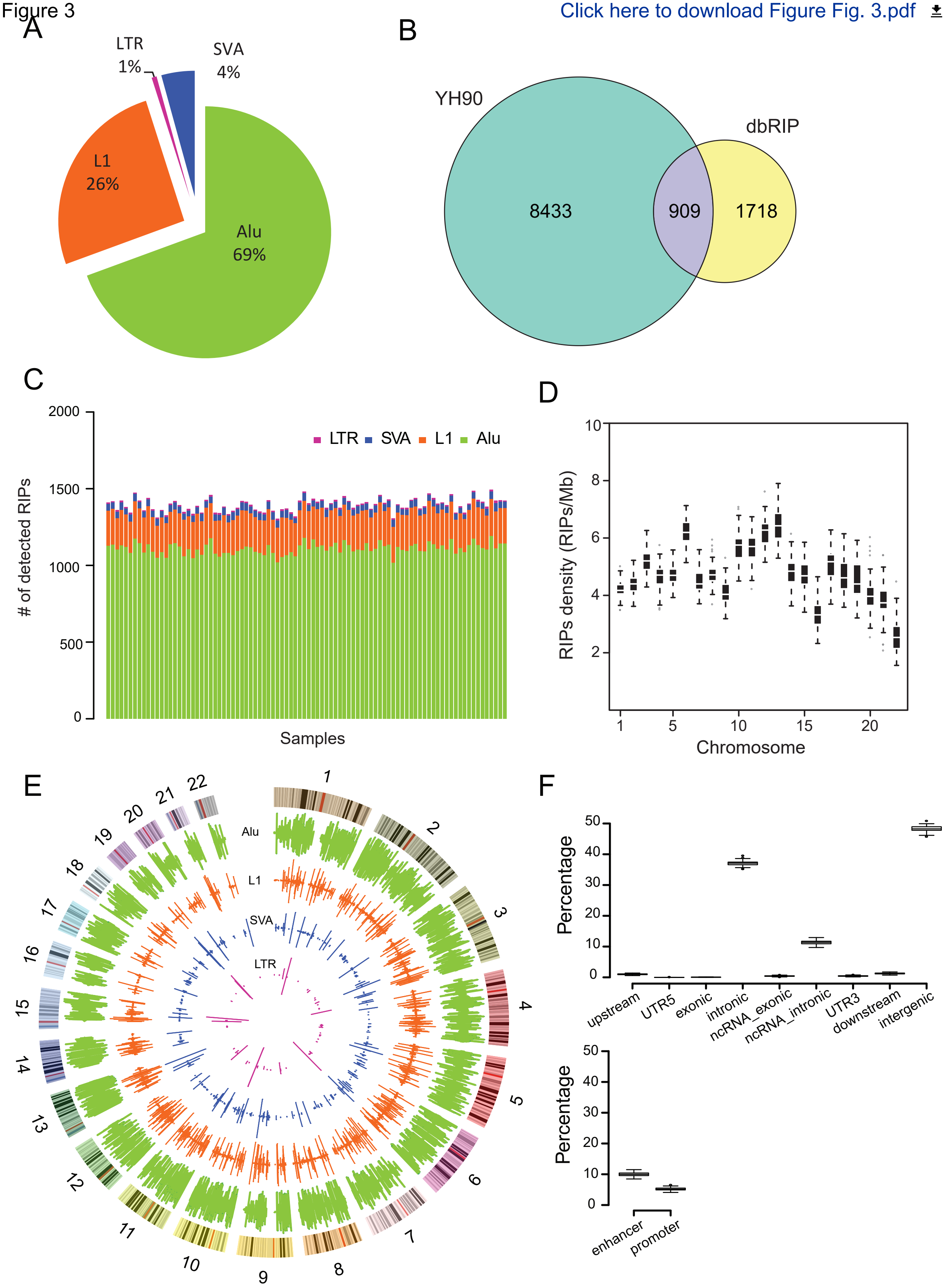

Figure 4

Click here to download Figure Fig. 4.pdf

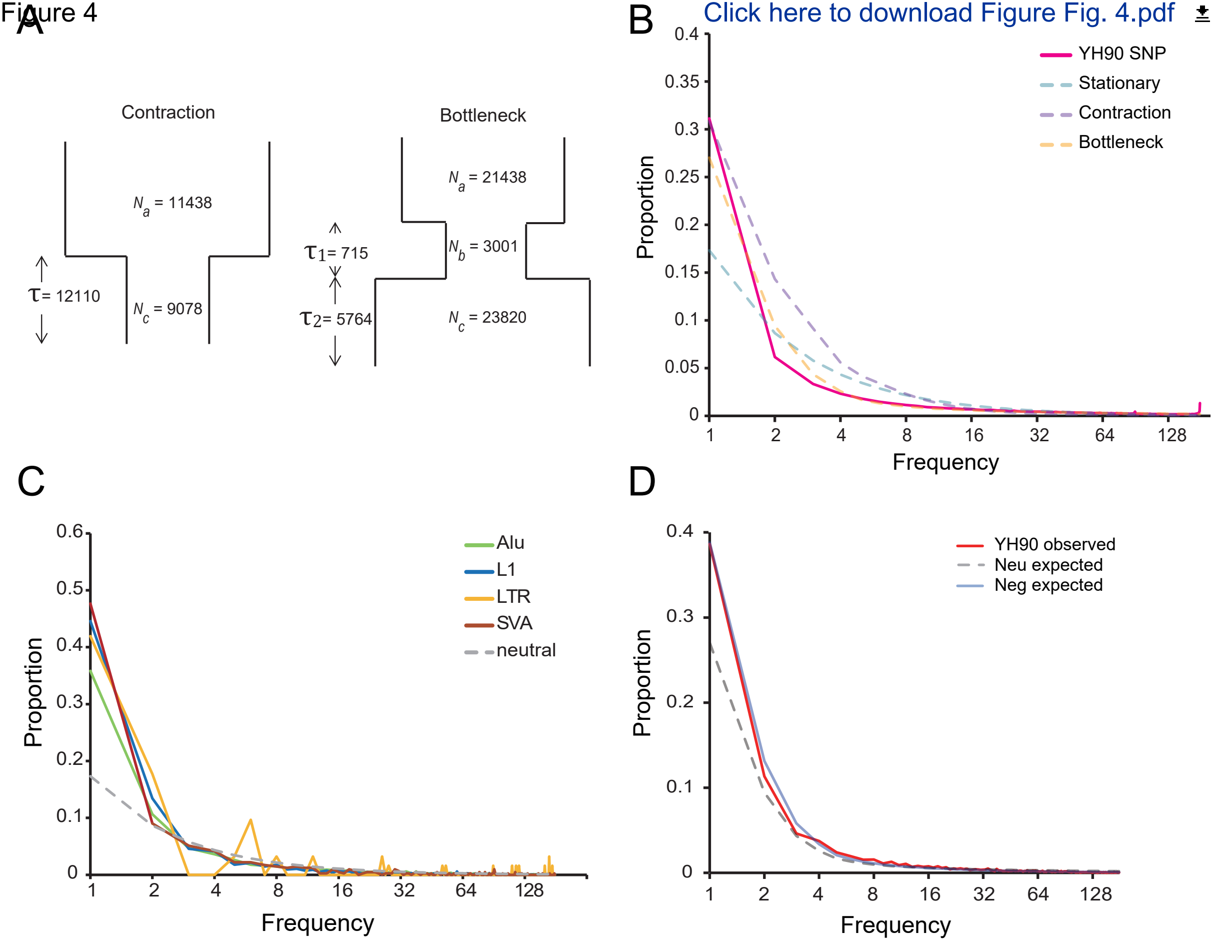

Figure 5

Click here to download Figure Fig. 5.pdf

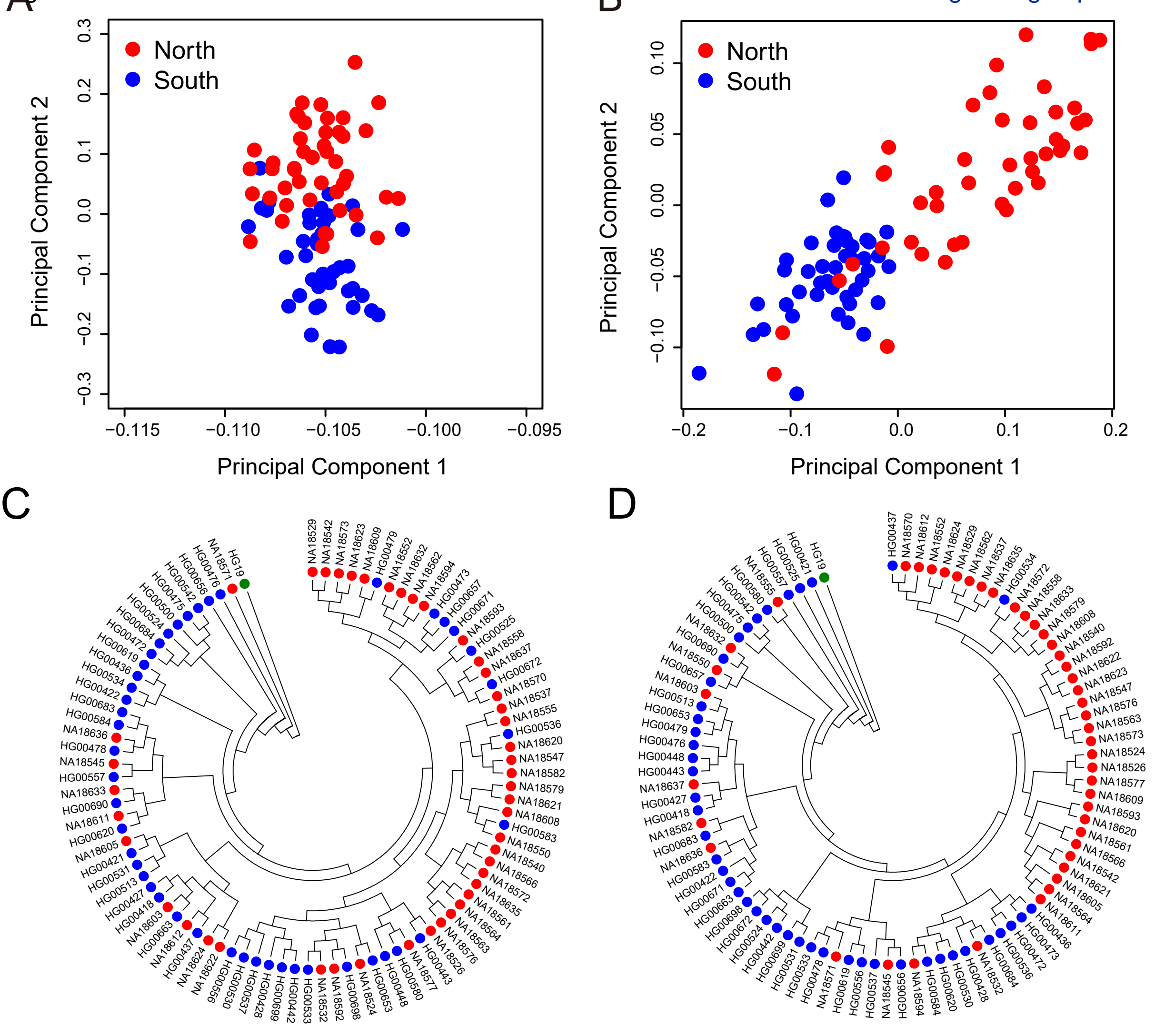

Additional file 1

Click here to access/download

Supplementary Material

Additional file 1.xlsx

Additional file 2

Click here to access/download
Supplementary Material
Additional file 2.pdf
