# Supplementary material for: Population-wide sampling of retrotransposon insertion polymorphisms using deep sequencing and efficient detection
Source: Gigascience. 2017 Jul 31;6(9):1–11. doi: 10.1093/gigascience/gix066 (PMC5603766; doi:10.1093/gigascience/gix066)
Supplement: Additional file 2: — Supplementary texts, figures, and tables (PDF 1120 kb). [file gix066_Additional_file_2.pdf]

Supplementary Materials

Supplementary Texts

Text S1: Alignment and SNP calling of the high-depth WGS data

The raw paired end 100bp (PE100) sequences in FASTQ files were aligned using BWA aln (v0.6.1)[1] with parameters '-n 3 -o 1 -e 50'. The reference genome was HG19 downloaded from UCSC genome browser. After conversion into BAM files, same sample files were merged using Samtools merge (v0.1.18)[2]. Whereafter, we marked duplicates using Picard MarkDuplicates.jar with default parameters. Then the BAM files were ordered using Picard (v1.61) ReorderSam.jar with default parameters. GenomeAnalysisTK version 2.0-36 [3] was used for local realignement under consideration of the known indels specified in the files 1000G_phase1.indels.HG19.sites.vcf and Mills_and_1000G_gold_standard.indels.HG19.sites.vcf (downloaded from Broad Institute website) with default configurations. We used GATK (version 2.0-36) to perform target realigning with parameters '-T RealignerTargetCreator' and -T IndelRealigner -model USE_SW -LOD 0.4. At last, we used GATK to perform base sore recalibration with parameters '-T CountCovariates -rf BadCigar' and '-T TableRecalibration'. All the BAM files were indexed using Samtools index (v0.1.18) with default parameter. Gene-based annotation for INDELs and SNPs were also performed using ANNOVAR (VersionDate 2014-11-12) [4] HG19 assembly. The resulting VCF files were used for subsequent analysis. The annotation for genes was Ensembl GRCh37.75 (HG19, downloaded from http://www.ensembl.org).

Text S2: RIP candidate determination

In general, detecting breakpoints of TE insertions is implemented after reads clustering, which is extremely time-consuming. To improve the efficiency of the algorithm, asynchronous scanning changes the order of this process. In a nutshell, unlike traditional algorithm that initiates detection of TE insertions after reads clustering is completed, asynchronous scanning algorithm begins the scanning for the breakpoints as soon as a putative TE insertion is identified. Hence, the detection of breakpoint will start earlier. Meanwhile, reads clustering is ongoing uninterruptedly. Therefore, the process of scanning potential breakpoints of for different TE insertions is asynchronous. As a consequence, asynchronous scanning algorithm considerably reduces the time for accurate detection of TE insertions.

The reliability of RIP candidate depends on several factors. We weighted these factors and implemented a score criterion to evaluate the confidence of detection result. The final score is composed of two parts, the score of the clustering and the score of the breakpoint detecting. The clustering has less weight than the breakpoint detecting due to mapping error. The total score of each candidate can be calculated as following:

$$S_{total} = S_C + S_B$$

in which,

$$S_C = \frac{\sum N_{main\_type} - V_{upper}}{W} + V_{upper}$$

$$S_B = \begin{cases} [S_{read} + S_{base} - (D - D_{threshold})/10]M, & D > D_{threshold} \\ (S_{read} + S_{base})M, & D \le D_{threshold} \end{cases}$$

in which,

$$S_{base} = \frac{\sum L_{clipped}}{10S_{read}}$$

where S_C indicates the score of clustering and S_B indicates the score of breakpoints detecting. N_{main_type} represents the reads number supporting the main type of retrotransposon supported by more than half of all reads. To reduce the impact of reads mapping depth on the S_{tatal}, especially for some regions with abnormal depth such as repeat regions, we set a theoretic adjusting value V_{upper} (in this study, $V_{upper} = 20$), and a weight value W (in this study, $W = 5$ for double-side clusters and $W = 10$ for single-side clusters) to weigh the reads number polarized V_{upper}. N_{bp} represents the number of reads which support the breakpoints and S_{base} stands for a weighted value measuring the clipped bases. $L_{clipped}$ stands for the length of each clipped read. In order to reduce the impact of ultra-depth impact on the S_B, we set a spanning breakpoint reads threshold $D_{threshold}$ (if the reads supporting the breakpoints depth D is more than $D_{threshold}$, the exceeding part would be corrected). M is also a weight value. For double-sites cluster, $M = 2.4$ when the reads number spanning the whole TSD sequence is less than 10 and the reads number that supports the insertion is more than 8 and $M = 1.2$ when the reads number spanning the whole TSD sequence is equal to or more than 10, or the reads number supporting the insertion is equal to or less than 8. For single-site clusters, $M = 2$ when the reads number spanning the whole TSD sequence is less than 10 and the reads number supporting the insertion is more than 5 and $M = 1$ when the reads number spanning the whole TSD sequence is equal to or more than 10, or the reads number supporting the insertion is equal to or less than 5.

When all these calculations are completed, we can get a score for each insertion and we filter out TE insertions with a score less than 15 to get the high confident insertions.

Text S3: Annotation for the TE insertions

If the orientation of the paired-end reads was different in one TE insertion, the TE insertion was judged to inversion and the orientation of insertion was judged by the orientation which more reads' orientation were the same with it. Insertions were annotated as having a poly-A tail if supporting reads had five or more consecutive 'A' bases among the six bases at the tail, and a poly-T annotation was assigned if supporting reads had five or more consecutive 'T' bases among the six bases at the head. In such conditions, the TE insertion was annotated as neither poly-A nor poly-T: 1) the insertion had reads supporting poly-A and reads supporting poly-T simultaneously, and 2) the insertion's orientation was different from that of the poly-A or poly-T, for example, the orientation of insertion was positive but the poly information was poly-T. The insert size was estimated by mapping all the supporting reads to a TE consensus sequence.
To ensure the complete diagnostic nucleotides in each subfamily sequence, we used Ns to fill the gaps of each subfamily sequence that did not harbor the diagnostic nucleotides sites. We determined the maximum similarity score (S_{MS}) for each subfamily based on a simple penalty algorithm as following:

$$S_{MS} = \sum S_i$$

where S_i indicates the score of the specific diagnostic nucleotide i. $S_i = 1$ when the query genotype was same as the diagnostic nucleotide i of this subfamily; $S_i = -0.5$ when the position of query contigs contained a gap while the diagnostic nucleotide i was not 'N', or the query contigs were mismatched against the diagnostic nucleotide i.

We also determined divergence value (V_D) for each subfamily as following:

$$V_D = \frac{N_{mis} + N_{gap}}{L_{map}}$$

where N_{mis} and N_{gap} indicated the number of mismatched base and the number of gaps of query contigs, respectively. The L_{map} stood for the mapped length of the certain subfamily. Subfamily with the maximum similarity based on the genotype of diagnostic nucleotide would be reported. If two or more subfamilies harbored the same maximum similarity, the subfamily with the smallest divergence value would be reported.

We treated the retrotransposon subfamily classification in dbRIP as ‘golden control’, and compared the classification result of 909 overlapped RIPs from our result with the golden control, to evaluate the accuracy of the subfamily classification. The assembled contigs of both 5’ and 3’ ends of insertions had the same orientation with HG19 sequence, which we defined as ‘positive orientation’. If the mapping orientations of the contigs were different, the orientation of TE insertion was judged from the mapping orientation supported by most contigs. Also, the poly-A tail of retrotransposon would be annotated if the TE insertion was ‘positive’ and there were more than four ‘A’ bases in the first 6 bases at 3’ end of the contigs. And the poly-T tail of the retransposon would be annotaed if the insertion orientation was ‘negative’ and there were more than four ‘T’ bases in the first 6 bases at 5’ end of the contigs.

Text S4: The assembly of junction sequences

After filtering the raw results of SID, the reads that supported certain TE insertions were extracted to FASTA files and used as the input for CAP3 (VersionDate: 02/10/15)[5]. We ran CAP3 with parameters ‘-i 21 -j 31 -o 16 -s 251 -p 70’. Then we merged all the contigs of the same sample to a FASTA file which was used as the query sequence of BLAST (v2.2.25)[6]. We ran formatdb (v2.2.25) with default parameters to build the database sequence for BLAST and then ran BLAST with parameters ‘-p blastn -F f -m 8’ to detect which part of the TE sequence was inserted into the genome. The results of CAP3 were uploaded to the website: https://github.com/Jonathanyu2014/SID.

Text S5: Calculation of accuracy

According to the results of PCR (Additional file 1: Table S8), among the 103 PCR experiments, there were 93 positive, 3 negative and 7 unknown samples. The lowest accuracy (A_L) was defined as:

$$A_L = \frac{N_P}{N_T}$$

Of which, $N_T = N_P + N_N + N_F$. N_P and N_N were the number of positive, negative validation results respectively. N_F and N_T were the failed and total number of PCR experiments respectively.
And the highest accuracy (A_H, also regarded as positive insertions rate R_P) was

$$A_H = \frac{N_P}{N_P + N_N}$$

Text S6: Prediction of Heterozygosity

To determine the genotype feature of each RIP, we considered two factors: one is the soft-clipped read that support insertions (N_C), the other is the spanned read that does not support insertions (N_S). We developed a simple Bayesian expression to estimate the posterior probability of the heterozygosity feature for each RIP. The posterior probability is:

$$P(Heter|N_C, N_S) = \frac{B(N_S, N_C + N_S, P_{HE})}{B(N_S, N_C + N_S, P_{HE}) + B(N_S, N_C + N_S, P_{HO})}$$

Where $B(k, N, P)$ represent the probability density function of binomial distribution. P_{HE} and P_{HO} represent the expected probability for heterozygosity and homozygosity respectively ($P_{HE} = 0.6$ and $P_{HO} = 0.1$).

MSL(Mean Structure Length) for each RIP was calculated as following:

$$MSL = -log_2\left(1 - P(Heter|N_C, N_S)\right)$$

And we determined the boundary line for heterozygosity and homozygosity as $MSL = 1$.

For each sample, we can estimate the hybrid rate:

$$R = \frac{N(MSL \geq 1)}{N(Total)}$$

The accuracy of this genotyping method was assessed according to the PCR validation genotypes of YH cell (Figure 2B and Additional file 1: Table S8) based on the number of bands. FDR was defined as the difference of heterozygosity (or/and homozygosity) estimated by MSL arithmetic and PCR validation divided by the total number of PCR experiments. The following table shows the FDR of this method in 2 samples.

Sample	Heteo(PCR)	Homo(PCR)	Heteo(MSL)	Homo(MSL)	FDR
YH_CL	13	36	11	38	4.08%
NA18571	24	14	23	15	2.63%

Heteo: heterozygous insertion. Homo: homozygous insertion. FDR: false discovery rate.

Text S7: Comparing with TEA and RetroSeq

We ran Retroseq (v1.41)[7] with parameters '-discover -refTEs ref_types.tab -eref probes.tab -align' and '-call filter ref_types.tab -reads 10 -depth 400' using the same BAM files with SID as input. The resulting VCF file was split into 2 files according to the type of TEs (Alu/L1). Then we filtered out the known hits using a BED file downloaded from the UCSC Genome Browser website. Hence, the result was used to compare to SID's

TEA [8] was downloaded from https://github.com/hastj7373/TEA. It used the "repeat anchored mate" (RAM) reads, which were uniquely mapped on the reference genome with paired mates mapped to TE sequences. To get the higher confident transposon insertions, we ran TEA's first two steps with default parameters to get the raw clusters and breakpoints, and then performed a rigorous filtering: a) the distance between two clipping sites was within [-15,30]bp and the ratio of clipped reads having the same clipping sites to the number of all clipped reads observed at an insertion site was above 0.5 and b) if the area between two clipping sites of one transposon insertion had overlaps with another insertion's, we considered them to be the same transposon insertion and combined them into one. The softwares that used in TEA were Samtools v0.1.18, BWA v0.6.1, BLAST v2.2.25 and CAP3 (VersionDate: 02/10/15). We removed the known hits and those in allosomes, then compared to the results of SID.

Text S8: Filtering and combination of the results of SID

For higher accuracy, we filtered out the records of SID which we thought were unauthentic if it met one of the following conditions:

(A) There was no specific TE block.

(B) The TSD was single-ended and the heterozygosity score estimated by SID was greater than 10.

(C) The confidence score was less than 15.

(D) The sequence that spanned TSD has poly-A/T at both 5' and 3'ends.

(E) There were no supporting clipped reads at 5' or 3' end of the inserted TE sequence or the TSD was single-side, but the total supporting reads at 5' or 3' end was greater than 100.

(F) There were supporting clipped reads at 5' or 3' end of the inserted TE sequence or the TSD was not single-ended, but the total supporting reads at 5' or 3' end was greater than 150.

(G) The length of poly-A/T sequence in TSD was longer than 15bp and the percentage of poly-A/T sequence accounted for 70% or more of the TSD sequence.

(H) The length of TSD sequence was longer than 20bp and the percentage of poly-A and poly-T sequence accounted for 70% and 80% or more of the TSD sequence respectively.

(I) The heterozygosity score was more than 10 and the number of total supporting reads was more than 80. Besides, the supporting clipped reads at 3' end accounted for less than 25% of total supporting reads but no supporting clipped reads at 5' end or the supporting clipped reads at 5' end accounted for less than 25% of total supporting reads but no supporting clipped reads at 3' end.

(J) All the reads that spanning certain TSD had more than 100 mismatches in total while there was no poly-A/T sequence in TSD.

We treated the TE insertions among different samples as the same one if it met the following two conditions simultaneously. (a) The inserted sequences were annotated as the same type of TE and (b) the regions of TSD were overlap at least 1 bp. Of note, we also listed the TE insertions that only meet condition (b) in Additional file 1: Table S11. These samples began with '|' for distinguishment. Then, we assembled the TSD sequences among YH90 samples that supported the same TE insertion and calculated the length of the combined TSD. The annotation of subfamily, the information of poly-A/T and the estimated insertion size were listed separately corresponding to the order of sample (Additional file 1: Table S11).

Hence, the start and end positions of a certain TSD were a result of assembly if it was a combination of more than one TE insertions. Low coverage around TSD region probably resulted in the different length of TSD sequences. Consequently, the TSD of a specific sample might not be the same with that in Additional file 1: Table S11.

Text S9: Allele frequency between YH90 and 1000GP SV dataset

The SV dataset of 1000GP phase 3 was downloaded from the website (http://www.internationalgenome.org/). We extracted the allele frequency of RIPs from all the samples and the five populations (EAS, EUR, AMR, AFR and SAS)[9]. The 3246 TE insertions shared by YH90 and 1000GP had exactly the same insertion sites and the TE types (Alu/SVA/L1). We calculated the Pearson correlation coefficient and significance of these 3246 TE insertions between YH90 and 1000GP using R command 'cor' and 'cor.test' with default parameters. The p-value between YH90 and each of the other population (EAS, EUR, AMR, AFR, SAS and the total 26 populations) was less than 2.2×10^{-16}.

Text S10: Population-based RIPs type correction

Because one site randomly inserted by retrotransposons twice is an extremely small probability event, we treated the same retrotransposons insertion of different samples as an inherent event. Hence, we re-corrected the inserted retrotransposons types based on the population scale result for each site in which several different retrotransposon types were annotated among positive samples. When the type with the most samples supporting was considered as correct type, the average match rate (the proportion of sites of which the type in accordance with the correct type of total sites) for all samples can reach up to 98.92% (the range from 97.52% to 99.49%). In fact, the primary type was supported by more than 80% of all positive samples for 71.05% of sites of divergence. Only 6.89% of the sites had the same percentage between two types and low insertion frequency ranging from 2.22% to 8.89%. Therefore, we adapted a strategy regarding the proportion of types in positive samples as weight to correct the inserted retrotransposons types. As mentioned above, the score criterion was the credibility of detection. Thus, we judged by the type with the maximum value, which was equal to multiply the sum of score by the proportion of supporters for each type.

Text S11: Inference of Fitness Effects of Recent RIPs

We estimated RIPs allele frequency spectra (AFS) based on the genotyping of each RIP (Fig 4c). Because the AFS was both influenced by the natural selection and the demographic history, we first inferred the demographic history to

eliminate the impact before the fitness test. Treating the selection on intergenic SNPs as neutral, we constructed the AFS of these SNPs of YH90. Using the AFS we inferred the best-fit history of instantaneous population size changes following the method of Williamson et al[10, 11], which determined the best-fit demographic model based on maximum likelihood evaluation. Then the distribution of fitness effects among non-reference RIPs was inferred under this best-fit demographic model as following:

$$\gamma = 2N_eS'$$

where γ indicates the fitness on the query RIPs, and N_e is the effective population size. s is the value of selective disadvantage (S' and $2S'$ are the selective disadvantage of heterozygotes and homozygous insertions). Then we can obtain the proportion of mutations that are strongly deleterious ($|S'| > 1\%$), moderately deleterious ($0.1\% < |S'| < 1\%$), weakly deleterious ($0.01\% < |S'| < 0.1\%$), and nearly neutral ($|S'| < 0.01\%$)[11]. We set several optional distributions (neutral selection + positive selection + negative selection, neutral selection + negative selection, and negative selection), and the fitness effect with the greatest likelihood value of each candidate selective model was calculated and was compared to the neutral model (ΔLL, the log-likelihood difference, Additional file 2：Figure S9 and Table S12).

Text S12: PCA analysis

After SNP calling, we used vcftools (v0.1.14)[12] to transform the genotype data to PLINK PED format with parameters '--vcf --plink'. Then we created a new PLINK 1 binary fileset using plink (v1.07)[13] with parameters '--noweb --make-bed' and calculated the genetic relationship matrix (GRM) from all the autosomal SNPs using GCTA (v1.25.2) [14] with parameters '--bfile --make-grm --autosome'. At last, we ran 'gcta64 --grm --pca 3' to obtain the top 3 principal components and drew the plot using R (v3.0.0). Of note, HG00418 and HG00427 were significantly deviated from the major population, so that the PCA plot did not show these two samples.

After the results of SID were filtered, we calculated the correlation coefficient of 9342 TEs between 90 samples. Then we used 'eigen' command in R program to compute eigenvalues and eigenvectors using these correlation coefficients. The first two vectors were used to perform PCA.

Text S13: phylogenetic analysis

We merged the SNPs of YH90 using vcftools (v0.1.14). The resulting VCF file was converted into FASTA file. Then we ran MEGA (v7.0.21) [15] on Microsoft Windows 10 OS. 'Compute Pairwise Distance' was used to obtain genetic distance. After that, we constructed phylogenetic tree using 'Construct/Test Neighbor-joining tree'. The phylogenetic analysis of RIPs was done using the similar method. At last, we ran 'Flip subtree' and 'Swab subtree' to display the tree more legibly.

Supplementary Figures

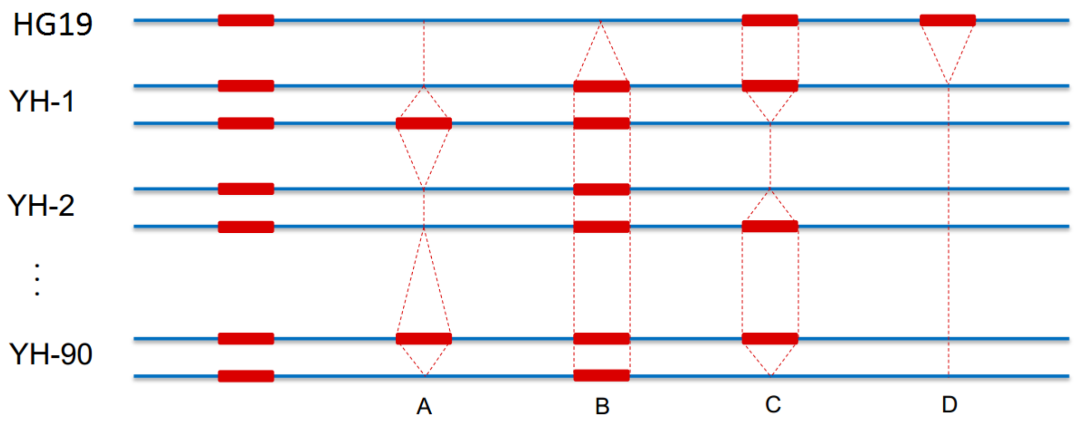

Figure S1. The polymorphism of retrotransposon insertions in HG19 and YH90. The blue lines stand for different alleles of samples. The red blocks stand for retrotransposon insertion elements. A and B are non-reference RIPs detected by SID. We named C and D as “reference RIPs”. We defined the YH90 polymorphism sites as A and C for the fitness analysis.

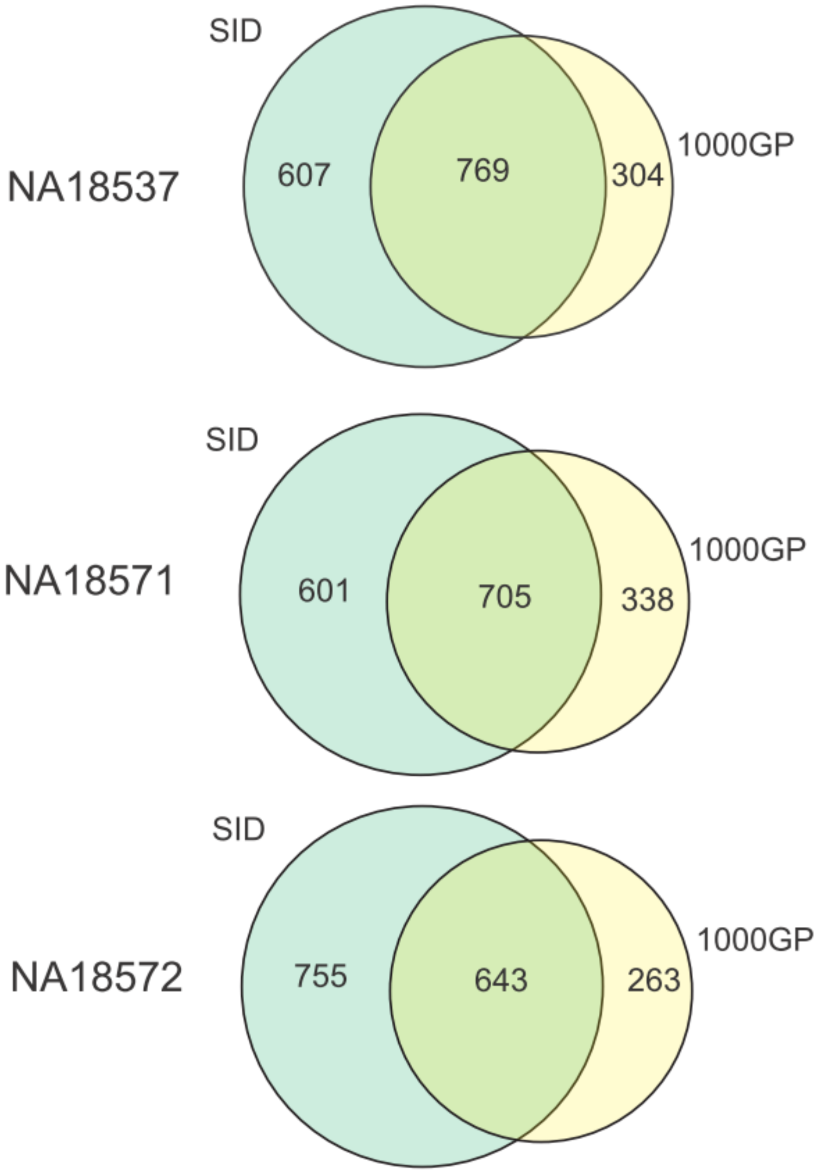

Figure S2. Comparison of our non-reference RIPs detection results (SID) with the 1000 Genomes Project Consortium's data. We used the 1000GP phase 3 release data[16].

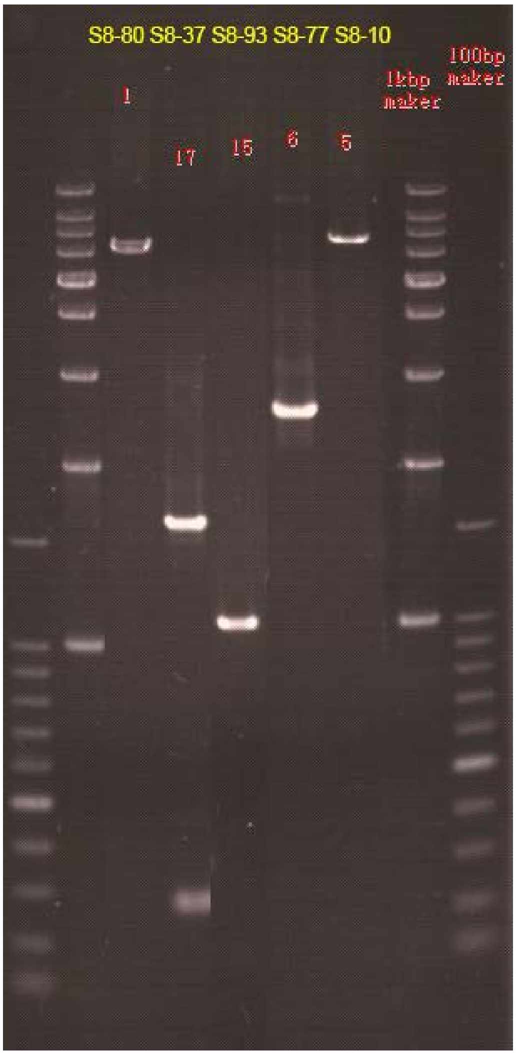
S8-80 S8-37 S8-93 S8-77 S8-10
100bp maker
1kbp maker
1
17
15
6
5

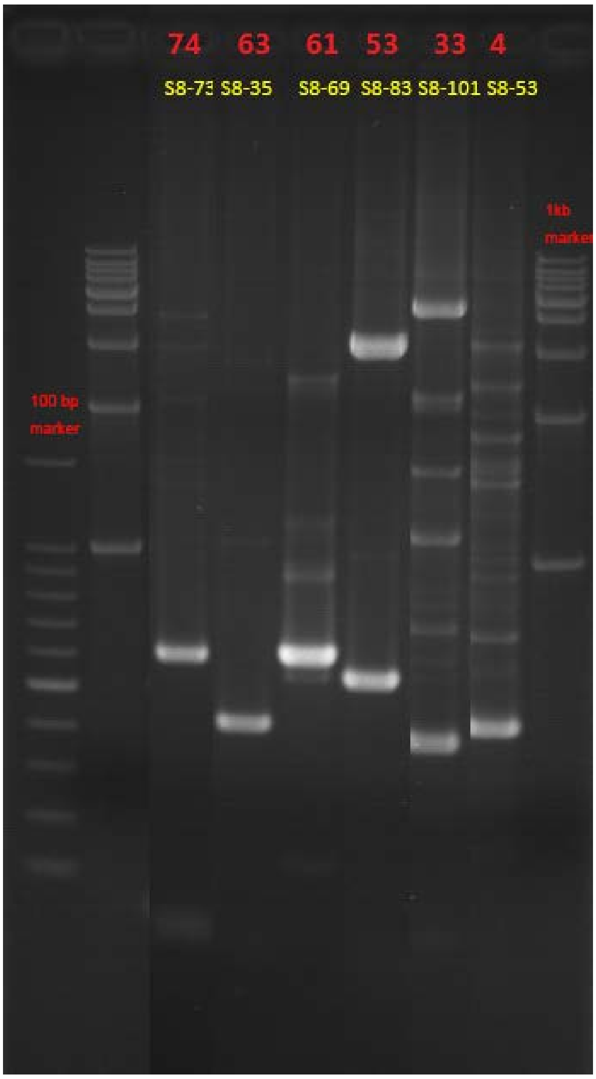
74 63 61 53 33 4
S8-73 S8-35 S8-69 S8-83 S8-101 S8-53
1kb marker
100 bp marker

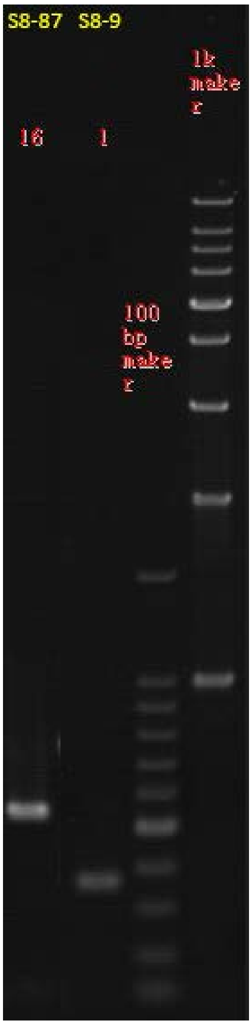
S8-87 S8-9
1k maker
16
1
100 bp maker

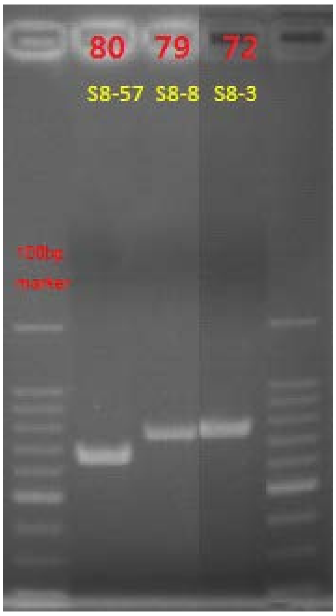
80 79 72
S8-57 S8-8 S8-3
100bp marker

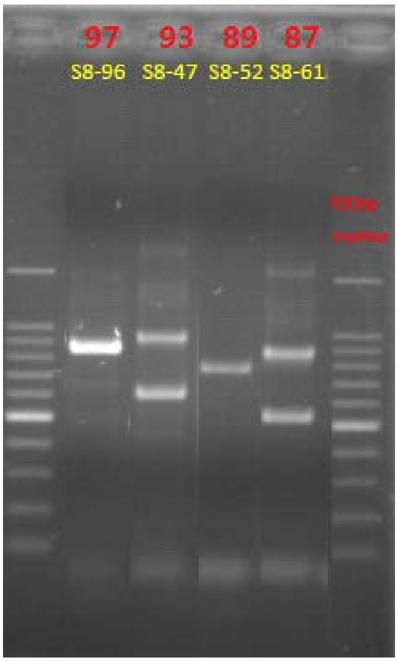
97 93 89 87
S8-96 S8-47 S8-52 S8-61
100bp marker

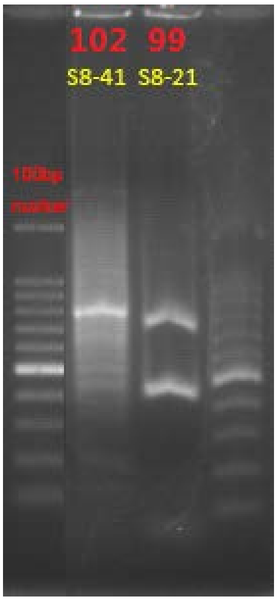
102 99
S8-41 S8-21
100bp marker

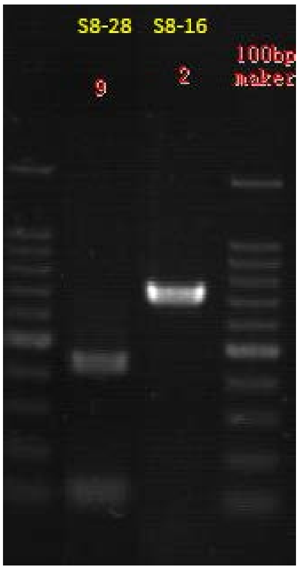
S8-28 S8-16
100bp maker
9
2

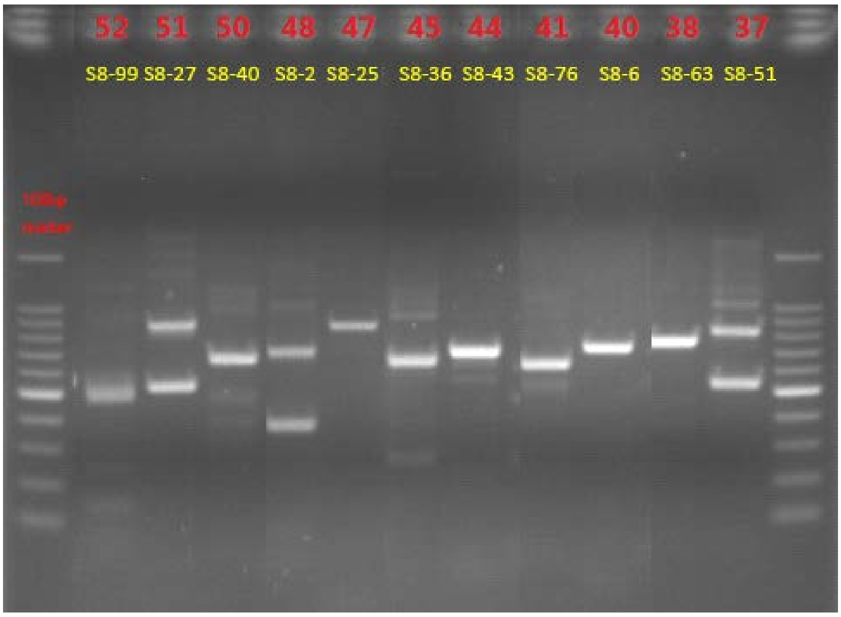
52 51 50 48 47 45 44 41 40 38 37
S8-99 S8-27 S8-40 S8-2 S8-25 S8-36 S8-43 S8-76 S8-6 S8-63 S8-51
100bp marker

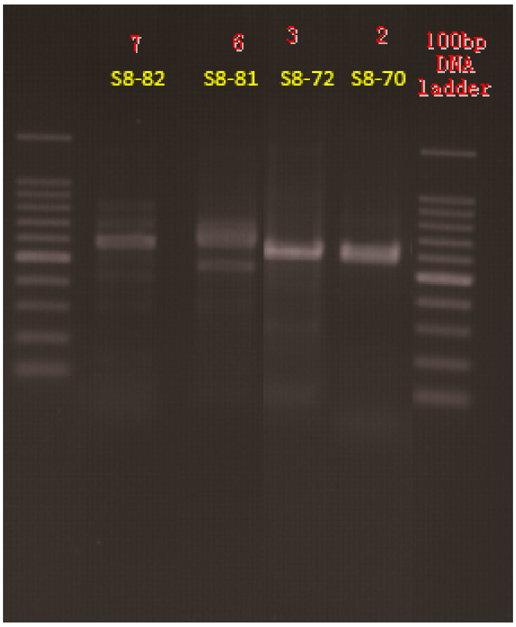
7 6 3 2
S8-82 S8-81 S8-72 S8-70
100bp DNA ladder

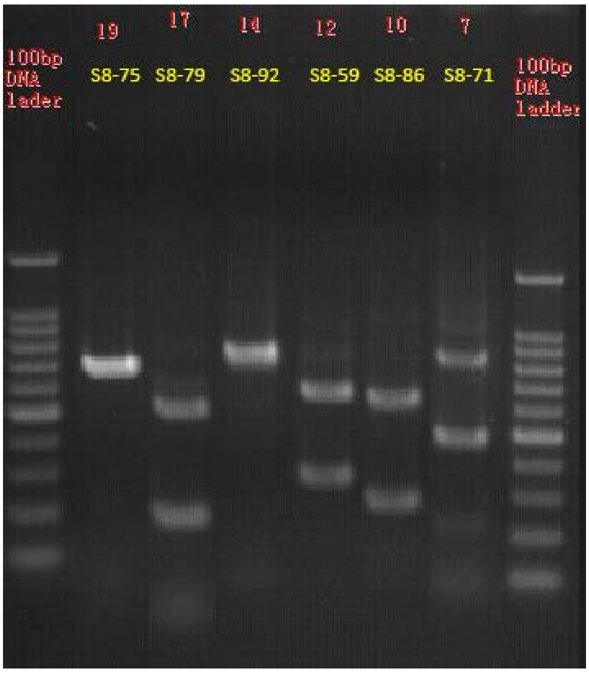
100bp DNA lader
19 17 14 12 10 7
S8-75 S8-79 S8-92 S8-59 S8-86 S8-71
100bp DNA ladder

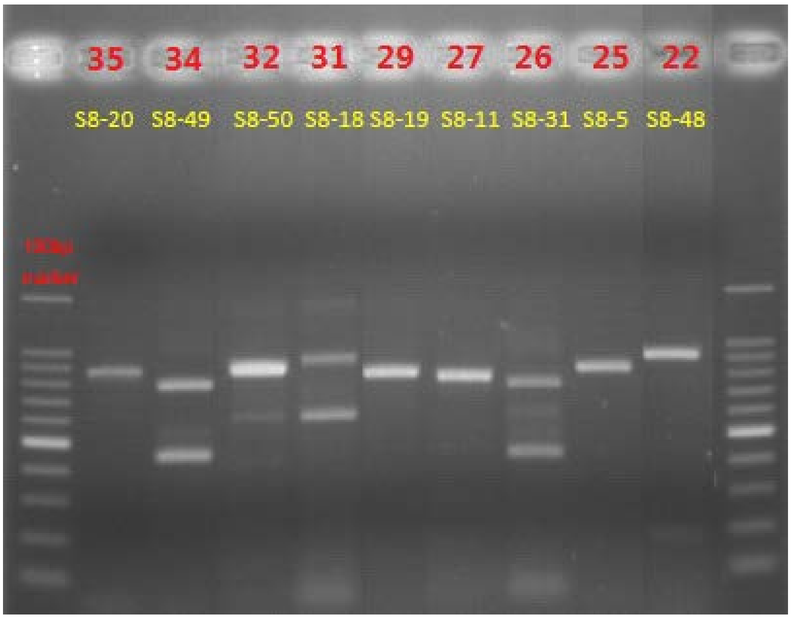
35 34 32 31 29 27 26 25 22
S8-20 S8-49 S8-50 S8-18 S8-19 S8-11 S8-31 S8-5 S8-48
100bp marker

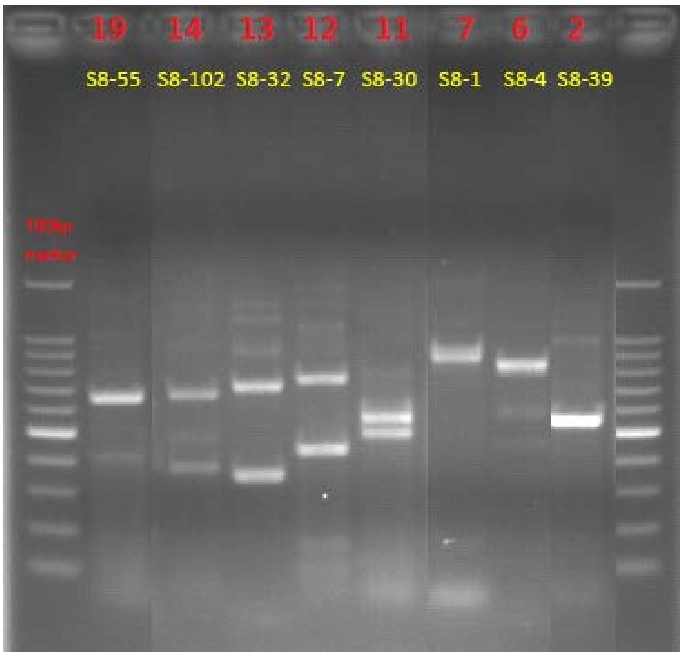
19 14 13 12 11 7 6 2
S8-55 S8-102 S8-32 S8-7 S8-30 S8-1 S8-4 S8-39
100bp marker

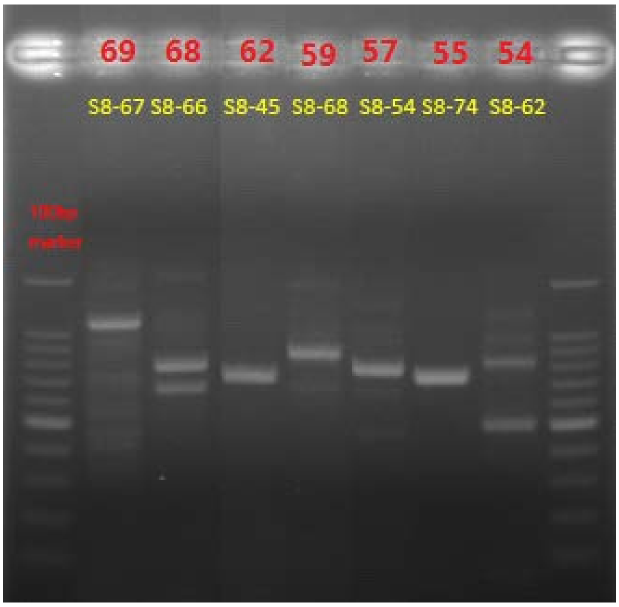
69 68 62 59 57 55 54
S8-67 S8-66 S8-45 S8-68 S8-54 S8-74 S8-62
100bp marker

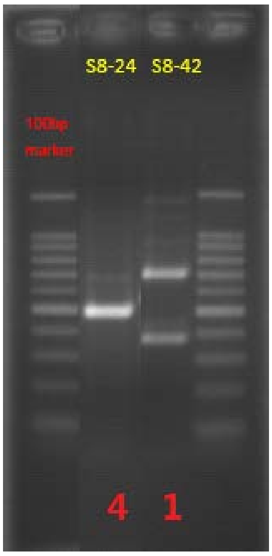
S8-24 S8-42
100bp marker
4 1

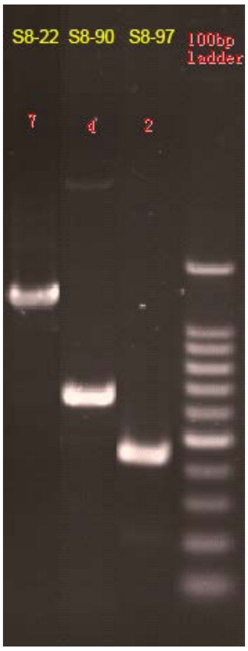
S8-22 S8-90 S8-97 100bp ladder
7 4 2

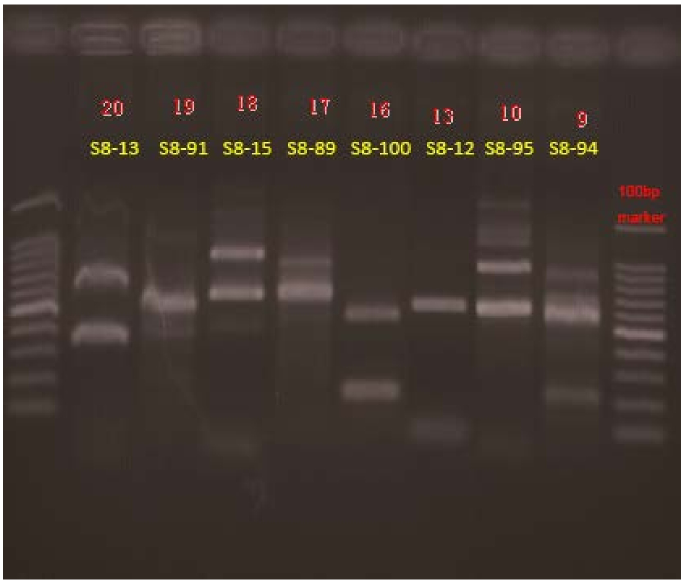
20 19 18 17 16 13 10 9
S8-13 S8-91 S8-15 S8-89 S8-100 S8-12 S8-95 S8-94
100bp marker

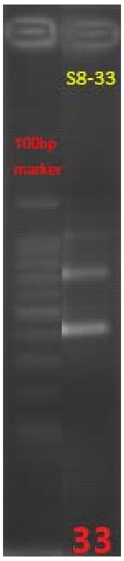
S8-33
100bp marker
33

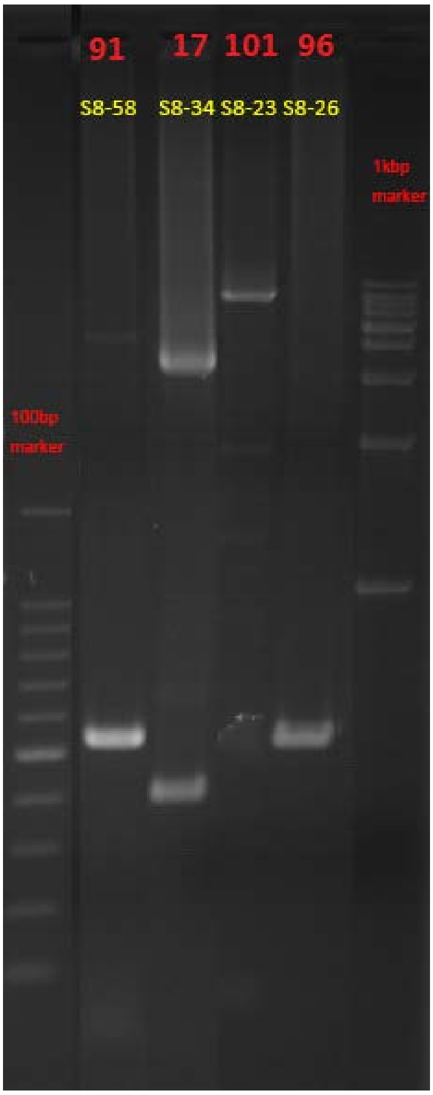
91 17 101 96
S8-58 S8-34 S8-23 S8-26
1kbp marker
100bp marker

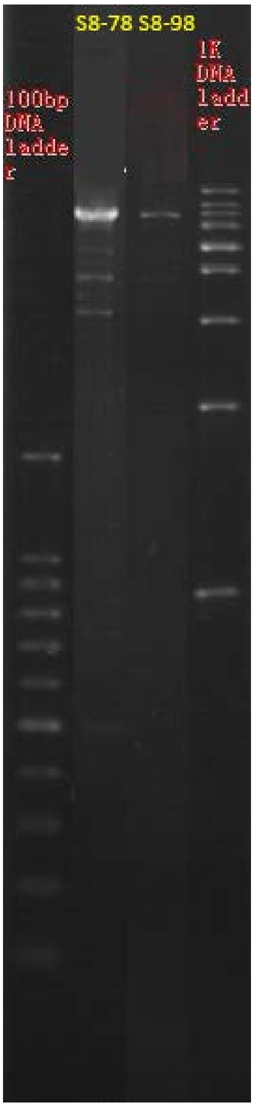
S8-78 S8-98
100bp DNA ladder
1K DNA ladder

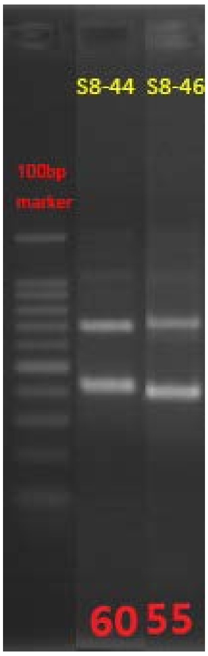
S8-44 S8-46
100bp marker
60 55

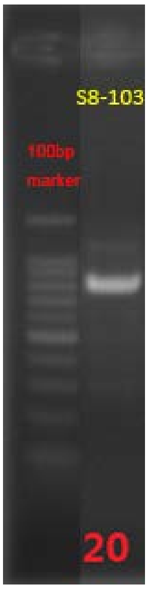
S8-103
100bp marker
20

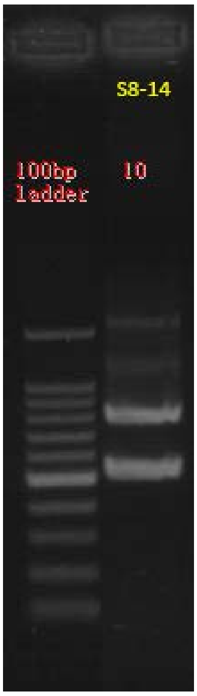
S8-14
100bp ladder
10

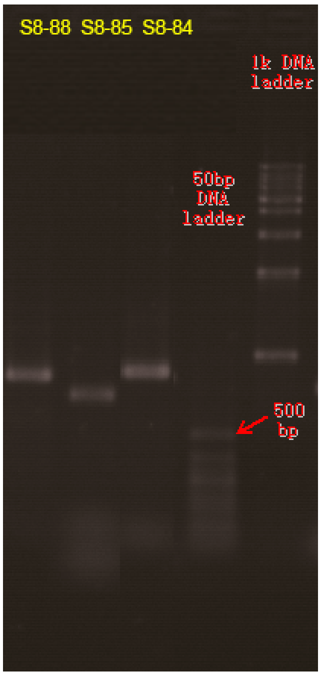

Figure S3. PCR validation of L1, Alu, LTR and SVA for YH_CL. The yellow words in the pictures are corresponding to Additional file 1: Table S8.

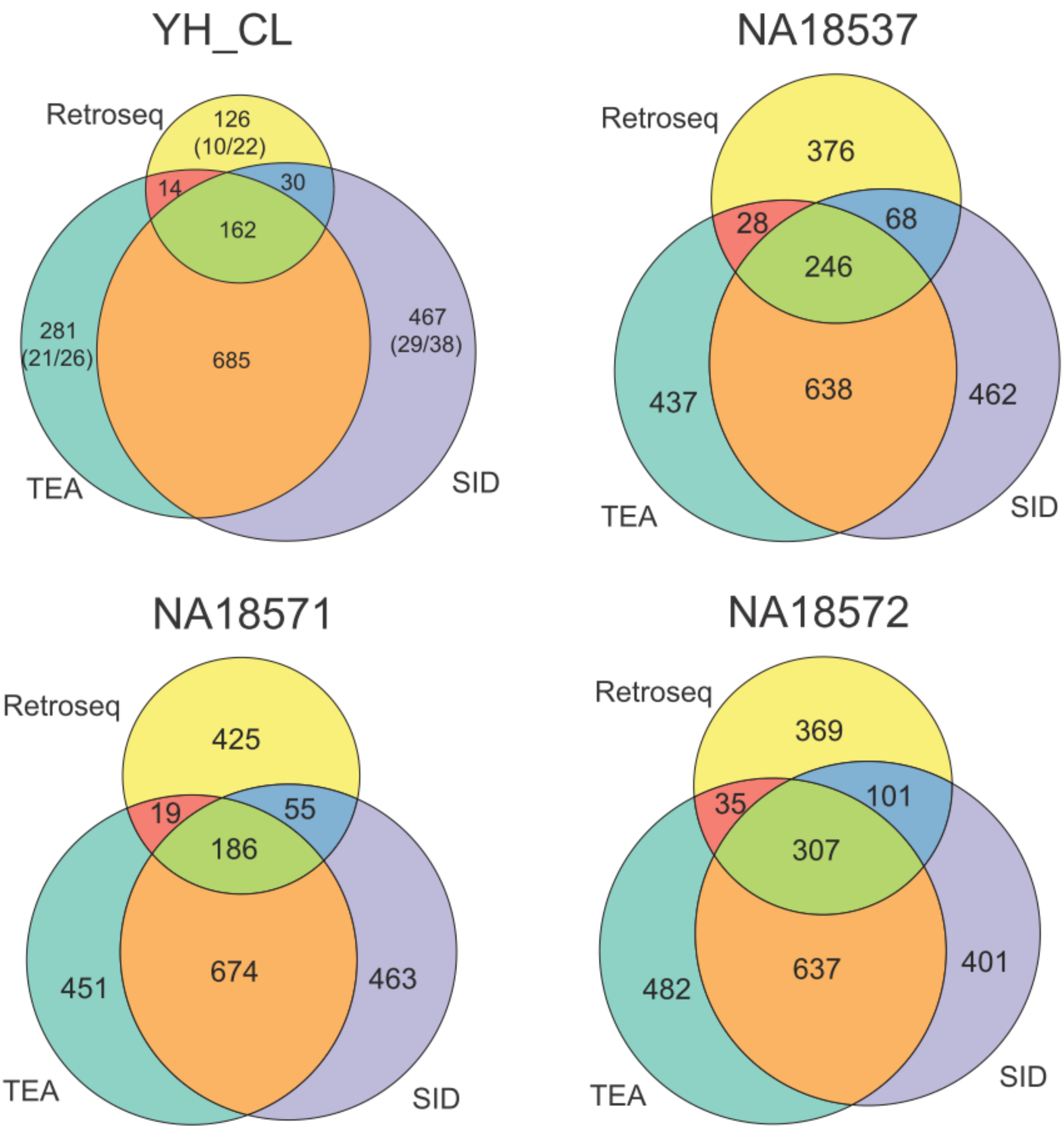

Figure S4. Comparison of non-reference RIPs detection results (SID) with two detection programs (TEA and Retroseq). We performed PCR validation for each unique TE insertion for YH_CL (Additional file 1: Table S10).

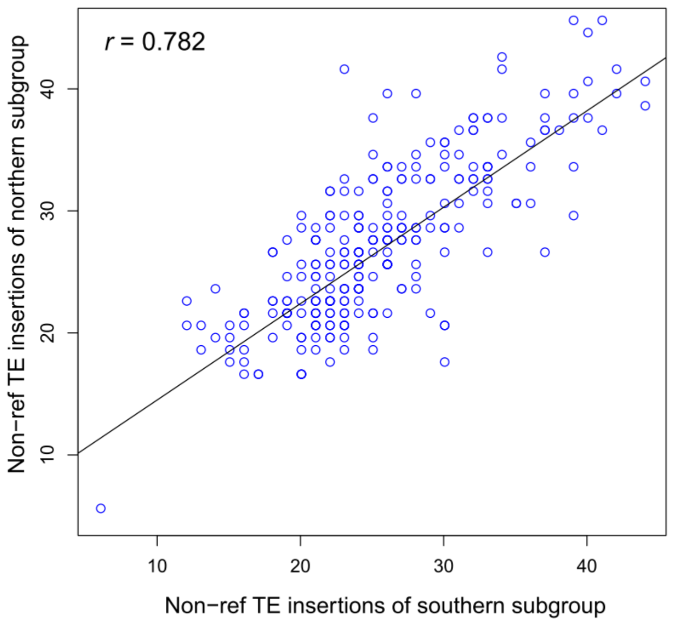

Figure S5. The correlation of numbers of non-reference RIPs between south and north subpopulations within 10M non-N windows.

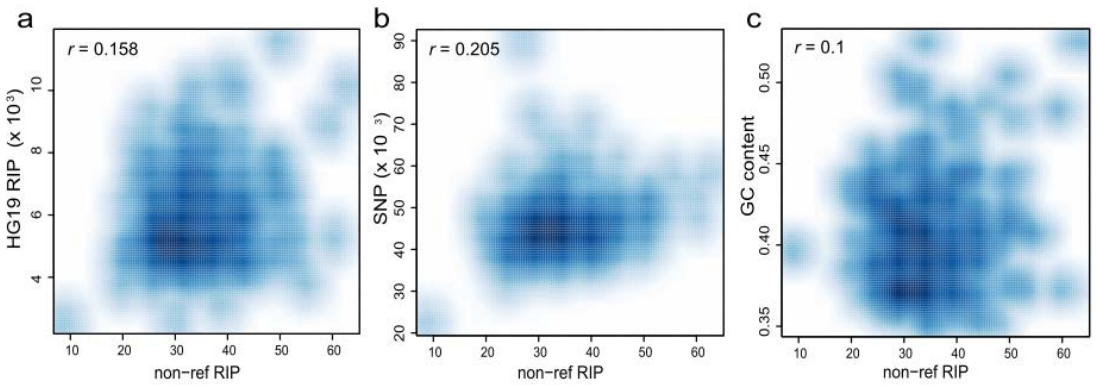

Figure S6. The correlation of number of RIPs sites within 10M non-N bins. (a) The correlation between HG19 RIPs that is not present in YH90 and non-reference RIPs we detected (P = 0.01). (b) The correlation between SNPs and non-reference RIPs (P = 0.001). (c) The correlation between GC content and non-reference RIPs (P = 0.11).

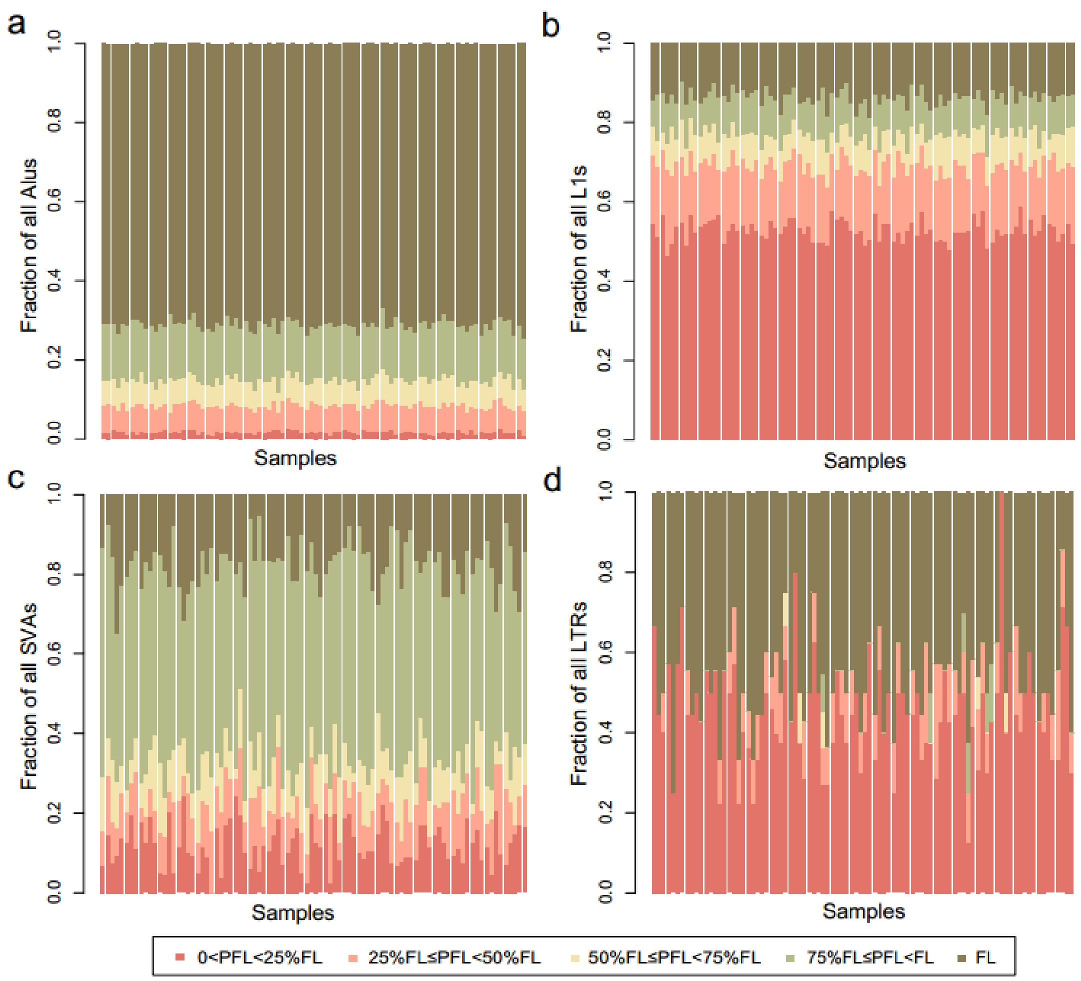

Figure S7. The length distribution of non-reference RIPs. (a) Length distribution of Alu insertions. (b) Length distribution of L1 insertions. (c) Length distribution of SVA insertions. (d) Length distribution of LTR insertions. PFL: percentage of full length of each retrotransposons subfamily. FL: full length of each retrotransposons subfamily.

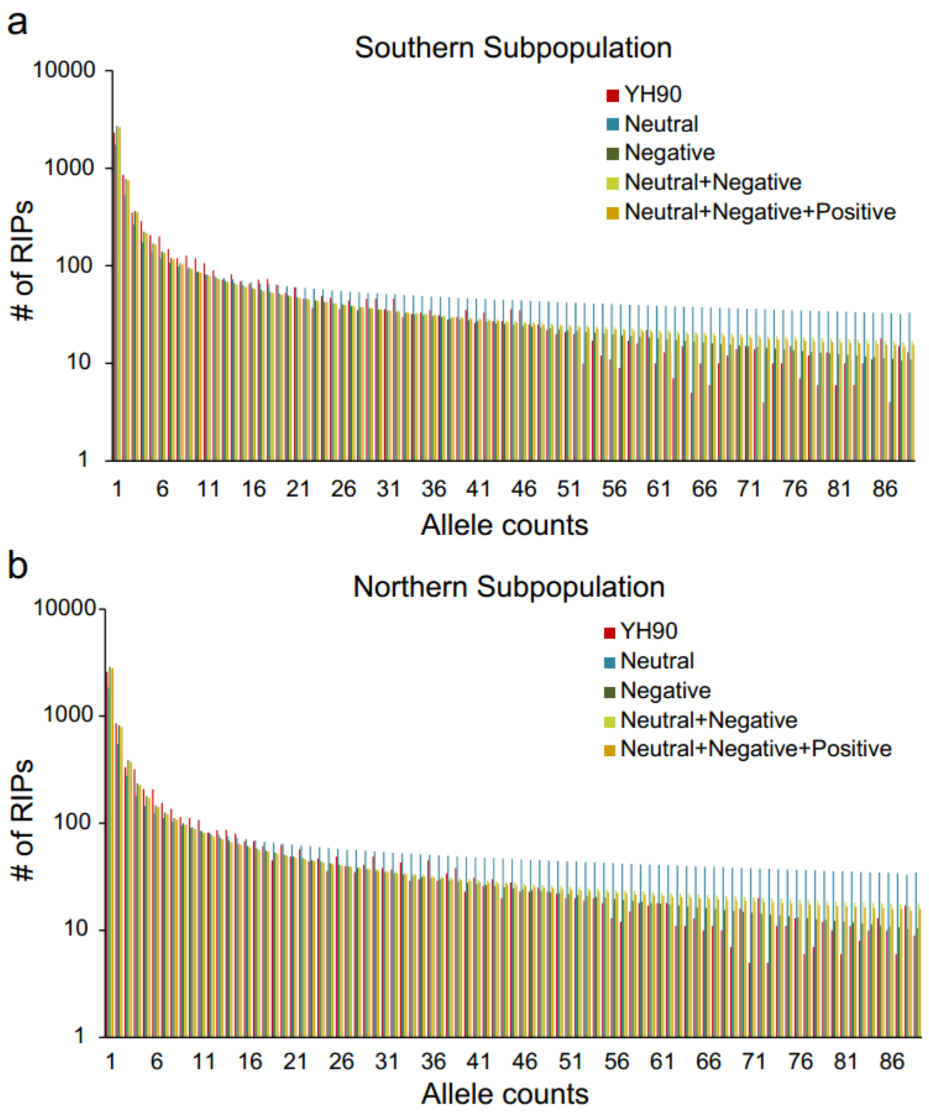

Figure S8. Observed and expected RIPs frequency spectra after demographic correction in each subpopulation. The expected RIPs frequency spectra under best-fit selection model after demographic correction. Note the logarithmic scale of the y-axis. (a) Southern subpopulation RIPs versus expectation under neutrality, fixed negative selective effects, weighted neutral and negative fitness effects and weighted neutral and negative and positive fitness effects. (b) Northern subpopulation RIPs versus expectation under neutrality, fixed negative selective effects, weighted neutral and negative fitness effects and weighted neutral and negative and positive fitness effects.

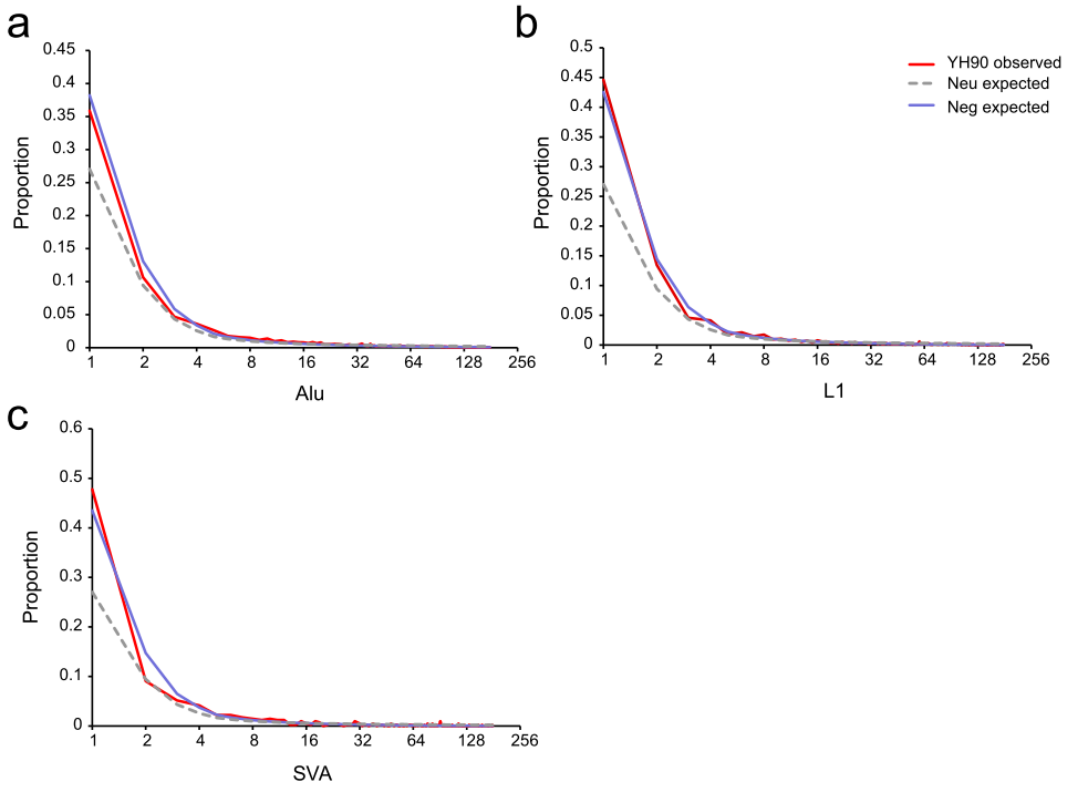

Figure S9. Observed and expected RIPs frequency spectra after demographic correction in each subfamily. The expected RIPs frequency spectra under the best-fit selection model after demographic correction. (a) Alu insertions versus expectation under neutrality and fixed negative selective effects. (b) L1 insertions versus expectation under neutrality and fixed negative selective effects. (c) SVA insertions versus expectation under neutrality and fixed negative selective effects.

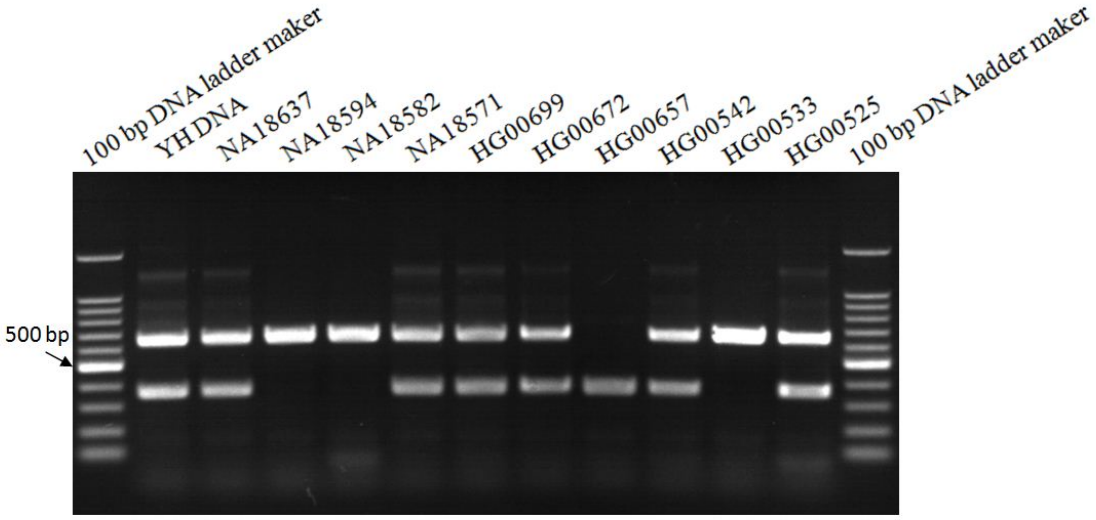

Figure S10. PCR validation of RIPs located in ACE for 11 individuals.

Supplementary Tables

Table S9. Run time for three different RIPs-detection programs.

Sample	Data size (GB)	Run time (h)		
		SID	TEA	RetroSeq
YH_CL	85	16.4	36.9	31.2
NA18571	127	19.9	90.6	110.3
NA18537	124	21.9	95.2	94.8
NA18572	117	31.9	82	99.8

Table S12. Maximum likelihood estimates of selection models.

Population	Model	ΔLL	*df*	Distribution	MLE
YH90	Neu	--	0	$P(\gamma = 0) = 1$	
YH90	Neu + Neg + Pos	928	4	$P(\gamma = 0) = p_0,$ $P(\gamma = k_1 < 0) = p_1,$ $P(\gamma = k_2 > 0) = 1 - p_0 - p_1$	$p_0 = 0.1,\ p_1 = 0.75,$ $k_1 = -13.8,\ k_2 = 0.001$
YH90	Neu + Neg	903	2	$P(\gamma = 0) = p_0,$ $P(\gamma = k < 0) = 1 - p_0$	$p_0 = 0.3,\ k = -18.87$
YH90	Neg	973	1	$P(\gamma = k < 0) = 1$	$k = -3.8$
Southern 45	Neg	697	1	$P(\gamma = k < 0) = 1$	$k = -3.65$
Northern 45	Neg	817	1	$P(\gamma = k < 0) = 1$	$k = -3.98$
YH90 (Alu)	Neg	420	1	$P(\gamma = k < 0) = 1$	$k_1 = -3.25$
YH90 (L1)	Neg	511	1	$P(\gamma = k < 0) = 1$	$k_1 = -6.8$
YH90 (SVA)	Neg	78	1	$P(\gamma = k < 0) = 1$	$k_1 = -8.2$

Note: MLE: maximum likelihood estimates, Neu: neutral, Neg: negative, Pos: positive.

Maximum likelihood estimates under each model computed after applying bottleneck demographic correction. Distributions are in terms of the scaled selection coefficient, $\gamma = 2N_{curr} * s$, where N_{curr} is 23832 in YH90 and two subpopulations. ΔLL is the log-likelihood difference between the neutral model and the overall best-fit model for the corresponding population[10, 11].

Table S13. Summary of best-fit demographic models.

Model	*df*	ΔLL	Population sizes	Expansion timings	Bottleneck duration
Stationary	0	396259	$N_e = 9710$	--	--
Contraction	2	368506	$N_{anc} = 11438,\ N_{curr} = 9078$	--	12110 gen
Bottleneck	4	--	$N_{anc} = 21438, N_{btl} = 3001,\ N_{curr} = 23820$	5764 gen ago	715 gen

ΔLL is the likelihood difference between the model and the overall best-fit model for the YH90. N_{anc} is the size of the effective ancestral population. N_{btl} is the size of effective population during the bottleneck. N_{curr} is the size of current effective population. τ is the time back to population size change, and it translates to generations with the formula(generations=2 $* N_{curr} *$ τ).

Reference

1. Li H and Durbin R. Fast and accurate short read alignment with Burrows-Wheeler transform. Bioinformatics. 2009;25 14:1754-60. doi:10.1093/bioinformatics/btp324.
2. Li H, Handsaker B, Wysoker A, Fennell T, Ruan J, Homer N, et al. The Sequence Alignment/Map format and SAMtools. Bioinformatics. 2009;25 16:2078-9. doi:10.1093/bioinformatics/btp352.
3. McKenna A, Hanna M, Banks E, Sivachenko A, Cibulskis K, Kernytsky A, et al. The Genome Analysis Toolkit: a MapReduce framework for analyzing next-generation DNA sequencing data. Genome research. 2010;20 9:1297-303. doi:10.1101/gr.107524.110.
4. Wang K, Li M and Hakonarson H. ANNOVAR: functional annotation of genetic variants from high-throughput sequencing data. Nucleic acids research. 2010;38 16:e164. doi:10.1093/nar/gkq603.
5. Huang X. CAP3: A DNA Sequence Assembly Program. Genome research. 1999;9 9:868-77. doi:10.1101/gr.9.9.868.
6. Mount DW. Using the Basic Local Alignment Search Tool (BLAST). CSH Protoc. 2007;2007:pdb top17. doi:10.1101/pdb.top17.
7. Keane TM, Wong K and Adams DJ. RetroSeq: transposable element discovery from next-generation sequencing data. Bioinformatics. 2013;29 3:389-90. doi:10.1093/bioinformatics/bts697.
8. Lee E, Iskow R, Yang L, Gokcumen O, Haseley P, Luquette LJ, 3rd, et al. Landscape of somatic retrotransposition in human cancers. Science. 2012;337 6097:967-71. doi:10.1126/science.1222077.
9. Sudmant PH, Rausch T, Gardner EJ, Handsaker RE, Abyzov A, Huddleston J, et al. An integrated map of structural variation in 2,504 human genomes. Nature. 2015;526 7571:75-81. doi:10.1038/nature15394.
10. Williamson SH, Hernandez R, Fledel-Alon A, Zhu L, Nielsen R and Bustamante CD. Simultaneous inference of selection and population growth from patterns of variation in the human genome. Proceedings of the National Academy of Sciences of the United States of America. 2005;102 22:7882-7. doi:10.1073/pnas.0502300102.
11. Boyko AR, Williamson SH, Indap AR, Degenhardt JD, Hernandez RD, Lohmueller KE, et al. Assessing the evolutionary impact of amino acid mutations in the human genome. PLoS Genet. 2008;4 5:e1000083. doi:10.1371/journal.pgen.1000083.
12. Danecek P, Auton A, Abecasis G, Albers CA, Banks E, DePristo MA, et al. The variant call format and VCFtools. Bioinformatics. 2011;27 15:2156-8. doi:10.1093/bioinformatics/btr330.
13. Chang CC, Chow CC, Tellier LC, Vattikuti S, Purcell SM and Lee JJ. Second-generation PLINK: rising to the challenge of larger and richer datasets. GigaScience. 2015;4:7. doi:10.1186/s13742-015-0047-8.
14. Yang J, Lee SH, Goddard ME and Visscher PM. GCTA: a tool for genome-wide complex trait analysis. Am J Hum Genet. 2011;88 1:76-82. doi:10.1016/j.ajhg.2010.11.011.
15. Kumar S, Stecher G and Tamura K. MEGA7: Molecular Evolutionary Genetics Analysis Version 7.0 for Bigger Datasets. Mol Biol Evol. 2016;33 7:1870-4. doi:10.1093/molbev/msw054.
16. Kang H, Zhu D, Lin R, Opiyo SO, Jiang N, Shiu SH, et al. A novel method for identifying polymorphic transposable elements via scanning of high-throughput short reads. DNA Res. 2016;23 3:241-51. doi:10.1093/dnares/dsw011.
