# Supplementary material for: Population-wide sampling of retrotransposon insertion polymorphisms using deep sequencing and efficient detection
Source: Gigascience. 2017 Jul 31;6(9):1–11. doi: 10.1093/gigascience/gix066 (PMC5603766; doi:10.1093/gigascience/gix066)
Supplement: Response-to-Reviewer-Comments_Original-Submission.pdf [file gix066_Response-to-Reviewer-Comments_Original-Submission.pdf]

Author's Response To Reviewer Comments

Dear editor,

We revised the manuscript and supplementary files carefully in terms of the referees' advice. To increase the usability of our software (SID), we fixed some bugs and uploaded necessary materials to the website of SID (https://github.com/Jonathanyu2014/SID) which included the instruction for the usage of SID, the data for testing, the explanation of the output and the reference TE sequences used in our study. The required softwares and packages as well as some helpful tips were also provided. All of these measures would make it easier to run SID.

To make our study reproducible, we provided a series of Supplementary Texts to explain how we accomplished our research in Additional file 2 in detail. Furthermore, we added ample Supplementary Tables and Figures to display our results.

The softwares (TEA/RetroSeq) that we used for comparison were the latest version when this study finished. Although TEA and RetroSeq are not the latest softwares for detection of mobile element insertion (MEI), they have been the most cited and influential tools for MEI calling by now. MELT, which a referee mentioned, was used by the 1000 Genomes Project Consortium (1000GP). We have updated our results on the comparison between SID and the 1000GP SV phase 3 dataset, which was also the latest version of 1000GP's SV dataset. The point-to-point reply is as follows.

Reviewer #1
1. Appropriate use of the 1000GP dataset and comparisons to previous software

Answer
We have updated to the latest version of the 1000GP SV dataset in our study. In total, we found 3264 TE insertions shared by YH90 and 1000GP dataset. The Pearson correlation coefficient is 0.7998 between YH90 and all the 26 populations in 1000GP phase 3 SV database. The Pearson correlation coefficient is 0.8856 between YH90 and EAS (East Asian) population in 1000GP dataset and 0.7662, 0.5741, 0.7025, 0.7627 for AMR, AFR, EUR and SAS respectively. These contents have been added to the main text line 243-249 and Additional file 2: Text S7.
YH90 and 1000GP dataset (phase 3) have some shared samples. We updated our results of comparison between SID and MELT using 3 common samples: NA18537, NA18571, NA18572, which were shown in Additional file 2: Figure S2.We also updated our citation to include the most recent paper from the consortium (Sudmant et al. 2015).
The description on page 3, lines 73-75 might be exaggerated. I have deleted these inexact statements.

2. Software parameters and reproducability

Answer
First, BWA aln is suitable for aligning paired-end 100 bp reads from Illumina sequencers. SID needs a series of specific tags (XT/X1/RG/MD) in BAM file that BWA aln could generate, while BWA mem could not generally. Besides, according to our comparison, there was no significant

difference of mapping rate between these two algorithms (n=3, depth = 60X. Paired-end 100 bp reads were simulated by pirs version 1.1. The mapping rate of both algorithms were >99.95%). We added the detailed pipeline and software parameters for SID/GATK/Samtools/Tea/Retroseq/Picard to Additional file 2: Text S1 and S7.
The detailed information of 761 TEs used for simulation were listed in Additional file 1: Table S6. Of note, whether a TE insertion could be detected by SID was decided not only by the number of reads that supporting the insertion, but also by the quality of supporting reads and some other minor factors.

3. Evaluation of accurate insertion detection

Answer
We attached the actual numbers respectively followed by the percentages. The definition of accuracy and positive insertion rate were provided in Additional file 2: Text S5.

The detailed command-line arguments were provided in Additional file 2: Text S1. The raw PCR validation results were also added to Additional file 2: Figure S3. The parameters used in our analysis were either recommended by the software or frequently-used arguments. For improving the overlap, impertinent parameterization might improve FDR instead of accuracy.

We have uploaded the junction sequences to the website of SID (https://github.com/Jonathanyu2014/SID) and described how we detected them in Additional file 2: Text S4.

4 Remarks concerning the software

Answer
The input BAM file for SID should be the output of BWA aln. According to our test, the BAM file should at least have XT, X1 and MD tags. If there was more than one BAM file as input, the RG tag was also necessary. Therefore, provided the BAM file met the above conditions, SID could run smoothly. We ran SID using Perl 5.14 and tested it using Perl 5.18. Hence, SID did not depend on Perl v5.22 or more advanced versions. Furthermore, we found that the error at line 403 was due to the older version of Samtools. We recommended users to use Samtools v1.0 or later.

There is an example on the website to show the detailed usage and explain the output of SID: https://github.com/Jonathanyu2014/SID.

5. Other remarks:

Answer
We compared the TEs (L1, SVA, Alu and LTR) in RepBase version 22.01 with our reference. The result (Table below) showed that our reference and the latest version of RebBase had many overlapping TEs. However, our reference had much more unique TEs than RepBase. Hence, our reference might detect much more specific sub-families of TE insertions than RepBase. Besides, the reference sequences used were uploaded to the website of SID:

https://github.com/Jonathanyu2014/SID.

Table 1. The number of 4 types of TEs between RepBase and our reference.

TE	RepBase*	SID**	#_overlapped_TEs
L1	117	619	114
Alu	58	2035	31
LTR	221	224	221
SVA	8	77	4

*The total number of certain type of TEs in RepBase version 22.01. Notably, we filtered the LTR whose ID did not begin with LTR. The data was extracted from humsub.ref and humrep.ref.
** The reference we used contains RepBase version 17.07, Hot L1s and dbRIP.

For the samples shared by YH90 and 1000GP, the sequencing depth was much deeper in YH90 dataset. Hence, there is no need for us to run SID using the data from 1000GP dataset. We believe that the differences of sequencing depth might influence the detection of ACE insertion.

The detailed methods with which we detected and analyzed SNPs were added to Additional file 2: Text S1, S12and S13. We did not compare SNPs with GC content.
The gene annotation version was Ensembl GRCh37.75, which we downloaded from ensembl.org. This information has been added to Additional file 2.
We deleted the incorrect description in Page 13, line 293.And we have substituted "human" with "Han Chinese" on line 252.

The sentence starting on line 193 was moved to Additional file 2: Text S3. We modified this sentence as follows “And the poly-T tail of the retransposon would be annotated if the insertion orientation was ‘negative’ and there were more than four ‘T’ bases in the first 6 bases at 5’ end of the contigs”.

Reviewer #2

1. I think the authors should either retailor the manuscript to discuss more of the algorithm, ie, how exactly they are improving accuracy (describe the maximal valid clusters algorithm and the Asynchronism [sic] Scanning algorithm"), or, provide more in-depth analyses of population genetics and breakpoints.

Answer
We have added necessary contents to Additional files and the website of SID, which made our analysis possible to be reproduced. Moreover, we performed more analyses on population genetics and phylogeny. Meanwhile, we simplified the description of the algorithm.

2. It's also unclear to me if this algorithm can perform genotyping of MEIs? It seems not from my read through of the text, but, this is not clear. This seems to be a functionality that is a necessity for any new algorithm in this field.

Answer
SID could perform genotyping of MEIs. It detects the insertion site of TEs at single-base resolution. The detailed information could be found on the website of SID (https://github.com/Jonathanyu2014/SID).

3.As a further validation of the utility of RIP, it would be good to show that the identified polymorphisms are useful markers of ancestry through a PCA or a tree.

Answer
We performed both PCA and phylogenetic analysis in the revised version of the paper (for details see main text section “Phylogenetic analysis”). Through a comparison with the results of SNPs, we proposed that RIPs could serve as markers of ancestry to some extent.

4. and 5. While to some extent both of the aforementioned issues are true, I believe they are overstated.There are English and grammatical errors throughout, and the manuscript will need to be carefully proofread.

Answer
We have revised our statements to make them clearer and more precise. We have proofread our manuscript and corrected grammatical errors carefully to make this paper easier to read and understand.

Best,
Shiping
