# Supplementary material for: Population-wide sampling of retrotransposon insertion polymorphisms using deep sequencing and efficient detection
Source: Gigascience. 2017 Jul 31;6(9):1–11. doi: 10.1093/gigascience/gix066 (PMC5603766; doi:10.1093/gigascience/gix066)
Supplement: Response-to-Reviewer-Comments_Revision-1.pdf [file gix066_Response-to-Reviewer-Comments_Revision-1.pdf]

Author's Response To Reviewer Comments

Dear reviewer and editor,

Thanks for reviewing our paper. We have carefully revised our paper according to your advice. The detailed information is as below.

1. When specific algorithms are mentioned e.g. "Maximal Valid Clusters" (line 117-8), and "Asynchronism Scanning" (line 136), these are not cited or clearly defined. It would be helpful to include either a reference if they were developed previous to this work, or a reference to where in the supplemental material these specific algorithms are described (I could not locate specific headings for these).

Answer：
Thanks. I added a reference (ref.21), which provides detailed description of Maximal Valid Clusters algorithm, in the main text (line 118).
For “Asynchronism Scanning”, we renamed it as “Asynchronous Scanning” for precisely meaning, which is not previously described. So, we explained this algorithm in Supplementary Materials (the first paragraph of Additional file 2: Text S2) briefly.

The newly added content in Additional file 2: Text S2 is as below.

In general, detecting breakpoints of TE insertions is implemented after reads clustering, which is extremely time-consuming. To improve the efficiency of the algorithm, asynchronous scanning changes the order of this process. In a nutshell, unlike traditional algorithm that initiates detection of TE insertions after reads clustering is completed, asynchronous scanning algorithm begins the scanning for the breakpoints as soon as a putative TE insertion is identified. Hence, the detection of breakpoint will start earlier. Meanwhile, reads clustering is ongoing uninterruptedly. Therefore, the process of scanning potential breakpoints of for different TE insertions is asynchronous. As a consequence, asynchronous scanning algorithm considerably reduces the time for accurate detection of TE insertions.

2. Define "AFS" where it is first used (line 314).

Answer:
Thanks. This error was corrected in line 316 and 328 in the updated manuscript.

3. I'm not sure that the claim, "This study is the first to evaluate the natural selection effect on retrotransposon insertions at the population level" is justified. For instance, Stewart et al (PLoS Genetics 2011) evaluate the allele frequency spectra of different populations using a similar approach.

Answer:
Thanks. Actually, the paper mentioned was cited in the main text (the 12th citation). We rewrote this sentence as “This study evaluates the natural selection effect on retrotransposon insertions at

the population level” (line 393-394).
