# Supplementary material for: Population-wide sampling of retrotransposon insertion polymorphisms using deep sequencing and efficient detection
Source: Gigascience. 2017 Jul 31;6(9):1–11. doi: 10.1093/gigascience/gix066 (PMC5603766; doi:10.1093/gigascience/gix066)
Supplement: Reviewer-1-Report-(Original-Submission).pdf [file gix066_Reviewer-1-Report-(Original-Submission).pdf]

Reviewer Report

Title: Population-wide Sampling of Retrotransposon Insertion Polymorphisms Using Deep Sequencing and Efficient Detection

Version: Original Submission **Date:** 1/28/2017

Reviewer name: Adam Ewing

Reviewer Comments to Author:

Qichao Yu and colleagues present a novel piece of software ("Specific Insertions Detection" or "SID") aimed at detection of transposable element insertions from whole-genome sequencing data. They use this tool to carry out an analysis of transposable element insertion polymorphisms among 90 Han Chinese individuals (45 Southern and 45 Northern) sourced from the Coriell repository. This is a welcome contribution to the available data on transposable element polymorphisms, and, moreover, appreciated as a contribution of control WGS datasets from Han Chinese populations. I have a number of concerns about the manuscript as it stands. Provided these can be addressed, especially the software usability issues, I feel that this work could be suitable for publication in GigaScience. I also think the manuscript could be substantially improved in terms of readability, and could use a thorough round of editing from a native English speaker as it was difficult to follow in spots due to language.1. Appropriate use of the 1000GP dataset and comparisons to previous softwareOn page 3, lines ~ 64-72, the 1000 Genomes Project (1000GP) is criticised for pooled low coverage sequencing leading to a bias towards rare RIP alleles. The authors seem to be in a position to test this assertion through comparison to their YH90 dataset: is there a correlation between RIP allele frequency in the YH90 set and concordance with the 1000GP set? Also, please use the latest version of the 1000GP SV dataset (Sudmant et al. 2015) when comparing against 1000GP (e.g. Fig S2), I think this might also improve concordance with previous results.Furthermore, regarding 1000GP, the most recent iteration of the SV dataset from this consortium was not cited (Sudmant et al. 2015), and the tool used to detect TE insertions was not tested (MELT: http://melt.igs.umaryland.edu/). In our hands, MELT is a very useable and capable tool for TE insertion detection and it would be good to see a comparison in the present manuscript, especially if the 1000GP dataset is to be used as a basis for comparison.Also on page 3, lines 73-75, it is stated that current tools "are challenged to deal with deep whole genome sequencing data…". It is perhaps worth pointing out that a number of tools including Tea (Lee et al. 2012), TraFiC (Tubio et al. 2014), and TranspoSeq (Helman et al. 2014), have been used to analyse hundreds of tumour/normal pairs from TCGA and/or ICGC, which is a dataset of comparable if not greater size than the YH90 dataset presented here. That said, it may be the case that SID is more computationally efficient at this scale, but that has not been shown (at least not convincingly) in the present manuscript.2. Software parameters and reproducabilityUnder the section "Processing of WGS data" (Page 4, starting line 97), the parameters "-n 3 -o 1 -e 50" seem to indicate that bwa aln was used and not bwa mem. It would be helpful to specify this explicitly as bwa aln and bwa mem are different aligners included in the same tool set. Moreover, if "bwa aln" was used, why? The more recent "bwa mem" is much more capable of detecting clipped reads which is critical for accurate structural variant detection from Illumina reads (including

transposable element detection). Using bwa mem might help alleviate the problem described on page 5-6, lines 126-139. Furthermore, please give explicit command line arguments for all tools including GATK, samtools, Tea, Retroseq, SID, etc. as well as version numbers, so that the analysis is reproducible. As it stands, little of the analysis in this manuscript would be considered reproducible due to lack of information.Also It would be helpful if the RIP simulation method was described in more detail: which 761 TEs were selected, where were they inserted, which ones were detectable at which depth. As it stands, the simulation is not reproducible.3. Evaluation of accurate insertion detectionI found it difficult to follow the various figures given for the accuracy/sensitivity of transposable element insertions reported by SID. Several different rates are given on pages 9 through 11: 95% accuracy for > 30x coverage in simulation versus 80.3% to 96.3% sensitivity depending on the TE family for the CEPH NA12891/92/78 trio, versus 90.29% - 96.88% accuracy via PCR validation on "selected sites". It would be helpful to know how accuracy and sensitivity were calculated: please state the actual numbers going into the respective calculation.These figures taken at face value might sound OK, but I am concerned about the accuracy of the given false positive rates because of the following:The 16.87% concordance rate between SID, Tea, and RetroSeq is quite low. Again, it would maybe be helpful to see exactly what command-line arguments were used for these tools. It is stated that "We also validated the uniquely detected RIPs by PCR" (lines 248-249) but I can't find the data on which RIPs these are nor is are the raw the PCR validation results included in the paper. Also, given this low concordance rate, the authors should explore why this is the case: are there parameterisations that would improve the overlap?The numbers of RIPs presented on page 12, line 259-268, seem quite high compared to previous work in this area and the information given in Table S7 is not sufficient to evaluate individual insertions. I have included a number of suggestions for Table S7 below.Table S7 (Polymorphic TE Insertions). This is an important table to understanding and evaluating the results of the YH90 analysis, and I feel that it is incomplete without, at a minimum, information on the junction sequences detected by SID: For any given insertion, I want to be able to align the junction sequences used to pinpoint the insertion to the reference (e.g. using BLAT) to evaluate whether the hallmarks of target-primed reverse transcription are present. These hallmarks include target site duplications (Maybe called "Breakpoint Sequences" in Table S7?), polyadenylation, endonuclease cleavage site, and the overall quality of the alignment versus the reference.4 Remarks concerning the software (SID, available at https://github.com/Jonathanyu2014/SID):As the software tool is a significant deliverable included in this manuscript, we sought to install and use the software to detect transposable elements in a small segment of a BAM file with known insertions, following the instructions included in "Usage.txt". It would be very helpful if a "test" or a small example BAM with the requisite files were available along with the software to ensure that we were attempting to use it properly. The issues encountered running the software included the following:There seems to be a requirement for bwa aln 0.6.1, as bwa mem generated BAMs and BAMs generated using later versions of bwa did not work, please state this in the documentation if it is the case.Please specify a version of BLAST to use, as "blastall" is only present in legacy versions of BLASTThere seems to be a version requirement for Perl, at least 5.22? Please include this information in the documentation.There are some hard-coded element names e.g. in the function at https://github.com/Jonathanyu2014/SID/blob/master/02cluster.pl#L233-L250 which suggests a specific input file format is required for the reference elements, but this is not clear from the usage information. Please include an example or explicit instructions on how to create the transposable element reference

file.We found we had to include a '-r' option to the system calls generated by the lines "print OUT "samtools merge $outDir/$Name.unique.bam"; (e.g. https://github.com/Jonathanyu2014/SID/blob/master/01discordant_v2.pl#L403) to avoid a samtools error.Given these issues and the unavailability of known good test data for SID we were unable to properly evaluate the software at this time, but with a little more work cleaning up some of the issues described above, and especially, providing a test dataset, we feel this has the potential to be a useful tool.5. Other remarks:Paragraph on page 7, lines 153-163: The various sources cited strike me as odd sources of information for consensus transposable element subfamilies: why not just obtain sequences from RepBase / Repeatmasker? Also it would be helpful to describe which subfamilies were represented (e.g. L1Ta and L1-preTa, SVA A-F, AluYa5, etc, etc). Also, in this paragraph, I think by "diagnosis position" you are referring to "diagnostic nucleotide" i.e. specific characters that define a subfamily. This is an example where the text needs to be clarified.Regarding the apparently high allele frequency ACE insertion found in YH90 but not in 1000GP, have the authors applied their tool to that region of 1000GP individuals to see if it is more apt to detect this particular insertion?Page 12, line 275, it was not clear how SNPs were being analysed, were these being compared to GC content as well? Also, how were SNPs detected?Page 13, Line 279, Please specify which gene annotation version was used to assess whether insertions were in genes.Page 13, line 293, Ref 28 (Boissinot et al, 2000) is not really an appropriate reference for the claim that most non-reference insertions are pre-Ta. In fact, most non-reference insertions should be from the more recent L1Ta subfamily.The section title on line 252 "A comprehensive RIPs landscape of human population" should probably refer to just the Han Chinese populations as all individuals in the YH90 dataset are from this population.The sentence starting on line 193 is a fragment.In the conclusions, the authors discuss using the RIPs detected from the YH90 dataset as a filter to eliminate germline insertions from papers regarding somatic insertions (e.g. in tumours). While this is true, larger and more comprehensive (in a population coverage sense) lists of RIPs exist: YH90 should be combined with 1000GP, dbRIP, euL1db, etc. to create as comprehensive a filter as possible for screening germline insertions. YH90 used by itself only covers specific populations.

Level of Interest

Please indicate how interesting you found the manuscript: An article whose findings are important to those with closely related research interests

Quality of Written English

Please indicate the quality of language in the manuscript: Not suitable for publication unless extensively edited

Declaration of Competing Interests

Please complete a declaration of competing interests, considering the following questions:

- Have you in the past five years received reimbursements, fees, funding, or salary from an organisation that may in any way gain or lose financially from the publication of this manuscript, either now or in the future?
- Do you hold any stocks or shares in an organisation that may in any way gain or lose financially from the publication of this manuscript, either now or in the future?
- Do you hold or are you currently applying for any patents relating to the content of the manuscript?
- Have you received reimbursements, fees, funding, or salary from an organization that holds or has applied for patents relating to the content of the manuscript?
- Do you have any other financial competing interests?
- Do you have any non-financial competing interests in relation to this paper?

If you can answer no to all of the above, write 'I declare that I have no competing interests' below. If your reply is yes to any, please give details below.

6. non-financial competing interests: I am developing software to detect transposable element insertions which would compete with the software presented in this manuscript, but feel that I can provide an unbiased review.

I agree to the open peer review policy of the journal. I understand that my name will be included on my report to the authors and, if the manuscript is accepted for publication, my named report including any attachments I upload will be posted on the website along with the authors' responses. I agree for my report to be made available under an Open Access Creative Commons CC-BY license (http://creativecommons.org/licenses/by/4.0/). I understand that any comments which I do not wish to be included in my named report can be included as confidential comments to the editors, which will not be published.

I agree to the open peer review policy of the journal
