# Supplementary material for: Population-wide sampling of retrotransposon insertion polymorphisms using deep sequencing and efficient detection
Source: Gigascience. 2017 Jul 31;6(9):1–11. doi: 10.1093/gigascience/gix066 (PMC5603766; doi:10.1093/gigascience/gix066)
Supplement: Reviewer-1-Report-(Revision-1).pdf [file gix066_Reviewer-1-Report-(Revision-1).pdf]

Reviewer Report

Title: Population-wide Sampling of Retrotransposon Insertion Polymorphisms Using Deep Sequencing and Efficient Detection

Version: Revision 1 **Date:** 6/5/2017

Reviewer name: Adam Ewing

Reviewer Comments to Author:

This manuscript is much improved, thank you for careful consideration and incorporation of my questions and suggestions at last review. I am satisfied that this can be published following a few minor issues being resolved, largely around improving clarity:1. When specific algorithms are mentioned e.g. "Maximal Valid Clusters" (line 117-8), and "Asynchronism Scanning" (line 136), these are not cited or clearly defined. It would be helpful to include either a reference if they were developed previous to this work, or a reference to where in the supplemental material these specific algorithms are described (I could not locate specific headings for these).2. Define "AFS" where it is first used (line 314).3. I'm not sure that the claim, "This study is the first to evaluate the natural selection effect on retrotransposon insertions at the population level" is justified. For instance, Stewart et al (PLoS Genetics 2011) evaluate the allele frequency spectra of different populations using a similar approach.

Level of Interest

Please indicate how interesting you found the manuscript: An article whose findings are important to those with closely related research interests

Quality of Written English

Please indicate the quality of language in the manuscript: Acceptable

Declaration of Competing Interests

Please complete a declaration of competing interests, considering the following questions:

- Have you in the past five years received reimbursements, fees, funding, or salary from an organisation that may in any way gain or lose financially from the publication of this manuscript, either now or in the future?
- Do you hold any stocks or shares in an organisation that may in any way gain or lose financially from the publication of this manuscript, either now or in the future?
- Do you hold or are you currently applying for any patents relating to the content of the manuscript?
- Have you received reimbursements, fees, funding, or salary from an organization that holds or has applied for patents relating to the content of the manuscript?

- Do you have any other financial competing interests?
- Do you have any non-financial competing interests in relation to this paper?

If you can answer no to all of the above, write 'I declare that I have no competing interests' below. If your reply is yes to any, please give details below.

n/a

I agree to the open peer review policy of the journal. I understand that my name will be included on my report to the authors and, if the manuscript is accepted for publication, my named report including any attachments I upload will be posted on the website along with the authors' responses. I agree for my report to be made available under an Open Access Creative Commons CC-BY license (http://creativecommons.org/licenses/by/4.0/). I understand that any comments which I do not wish to be included in my named report can be included as confidential comments to the editors, which will not be published.

I agree to the open peer review policy of the journal
