# Supplementary material for: Population-wide sampling of retrotransposon insertion polymorphisms using deep sequencing and efficient detection
Source: Gigascience. 2017 Jul 31;6(9):1–11. doi: 10.1093/gigascience/gix066 (PMC5603766; doi:10.1093/gigascience/gix066)
Supplement: Reviewer-2-Report-(Original-Submission).pdf [file gix066_Reviewer-2-Report-(Original-Submission).pdf]

Reviewer Report

Title: Population-wide Sampling of Retrotransposon Insertion Polymorphisms Using Deep Sequencing and Efficient Detection

Version: Original Submission **Date:** 2/7/2017

Reviewer name: Peter Sudmant

Reviewer Comments to Author:

In this manuscript Yu and colleagues present a new tool to identify mobile element insertions (referred to in this paper as retrotransposon insertion polymorphisms , RIPs). The authors employ their algorithm on 90 high-coverage sequenced Han Chinese individuals. The authors demonstrate that their algorithm is sufficiently faster (~3-fold) than previous algorithms, and more accurate, and present some very simple observations about MEIs in their dataset.In general, I think this algorithm is a valuable contribution and will be an excellent tool for individuals interested in MEIs. The manuscript lacks much detail however and the analyses provide little insights on population genetics of MEIs or the breakpoints / mechanisms etc. I think the authors should either retailor the manuscript to discuss more of the algorithm, ie, how exactly they are improving accuracy (describe the maximal valid clusters algorithm and the Asynchronism [sic] Scanning algorithm"), or, provide more in-depth analyses of population genetics and breakpoints.It's also unclear to me if this algorithm can perform genotyping of MEIs? It seems not from my read through of the text, but, this is not clear. This seems to be a functionality that is a necessity for any new algorithm in this field.The authors identify MEIs in individuals from different reginos of China. As a further validation of the utility of RIP, it would be good to show that the identified polymorphisms are useful markers of ancestry through a PCA or a tree. (Genotyping would be important here)If the authors are interested in further population genetics analyses, they might consider analyzing Simons dataset, 300 high coverage genomes from across the world: Nature. 2016 Oct 13;538(7624):201-206. doi: 10.1038/nature18964. Epub 2016 Sep 21. The Simons Genome Diversity Project: 300 genomes from 142 diverse populations. Mallick et alThe authors claim that efforts to identify MEI polymorphisms "have not been undertaken sufficiently" and the reasons for this are that efforts have only been undertaken in low-coverage samples such as the 1000 genomes and additionally software has not been fast enough to process high-coverage data. While to some extent both of the aforementioned issues are true, I believe they are overstated.There are English and grammatical errors throughout, and the manuscript will need to be carefully proofread

Level of Interest

Please indicate how interesting you found the manuscript: An article whose findings are important to those with closely related research interests

Quality of Written English

Please indicate the quality of language in the manuscript: Needs some language corrections before being published

Declaration of Competing Interests

Please complete a declaration of competing interests, considering the following questions:

- Have you in the past five years received reimbursements, fees, funding, or salary from an organisation that may in any way gain or lose financially from the publication of this manuscript, either now or in the future?
- Do you hold any stocks or shares in an organisation that may in any way gain or lose financially from the publication of this manuscript, either now or in the future?
- Do you hold or are you currently applying for any patents relating to the content of the manuscript?
- Have you received reimbursements, fees, funding, or salary from an organization that holds or has applied for patents relating to the content of the manuscript?
- Do you have any other financial competing interests?
- Do you have any non-financial competing interests in relation to this paper?

If you can answer no to all of the above, write 'I declare that I have no competing interests' below. If your reply is yes to any, please give details below.

I declare that I have no competing interests

I agree to the open peer review policy of the journal. I understand that my name will be included on my report to the authors and, if the manuscript is accepted for publication, my named report including any attachments I upload will be posted on the website along with the authors' responses. I agree for my report to be made available under an Open Access Creative Commons CC-BY license (http://creativecommons.org/licenses/by/4.0/). I understand that any comments which I do not wish to be included in my named report can be included as confidential comments to the editors, which will not be published.

I agree to the open peer review policy of the journal
